# De novo design of highly selective miniprotein inhibitors of integrins αvβ6 and αvβ8

**DOI:** 10.1038/s41467-023-41272-z

**Published:** 2023-09-13

**Authors:** Anindya Roy, Lei Shi, Ashley Chang, Xianchi Dong, Andres Fernandez, John C. Kraft, Jing Li, Viet Q. Le, Rebecca Viazzo Winegar, Gerald Maxwell Cherf, Dean Slocum, P. Daniel Poulson, Garrett E. Casper, Mary L. Vallecillo-Zúniga, Jonard Corpuz Valdoz, Marcos C. Miranda, Hua Bai, Yakov Kipnis, Audrey Olshefsky, Tanu Priya, Lauren Carter, Rashmi Ravichandran, Cameron M. Chow, Max R. Johnson, Suna Cheng, McKaela Smith, Catherine Overed-Sayer, Donna K. Finch, David Lowe, Asim K. Bera, Gustavo Matute-Bello, Timothy P. Birkland, Frank DiMaio, Ganesh Raghu, Jennifer R. Cochran, Lance J. Stewart, Melody G. Campbell, Pam M. Van Ry, Timothy Springer, David Baker

**Affiliations:** 1 Department of Biochemistry and Institute for Protein Design, University of Washington, Seattle, WA 98195 USA; 2 Department of Chemistry and Biochemistry, Brigham Young University, Provo, UT 84602 USA; 3 Program in Cellular and Molecular Medicine, Children’s Hospital Boston, and Departments of Biological Chemistry and Molecular Pharmacology and of Medicine, Harvard Medical School, Boston, MA USA; 4 Division of Basic Sciences, Fred Hutchinson Cancer Center, Seattle, WA 98109 USA; 5 Department of Bioengineering, Stanford University, Stanford, CA 94305 USA; 6 Howard Hughes Medical Institute, University of Washington, Seattle, WA 98195 USA; 7 Department of Bioengineering, University of Washington, Seattle, WA 98195 USA; 8 Department of Materials Science and Engineering, University of Washington, Seattle, WA 98195 USA; 9 Research and Early Development, Respiratory and Immunology, BioPharmaceuticals R&D, AstraZeneca, Cambridge, UK; 10 Center for Lung Biology, Division of Pulmonary, Critical Care and Sleep Medicine, University of Washington, Seattle, USA; 11 Division of Pulmonary, Critical Care and Sleep Medicine, Department of Medicine, University of Washington, Seattle, WA USA; 12 Dept of Medicine, University of Washington, Seattle, WA USA; 13 Present Address: Encodia Inc, 5785 Oberlin Drive, San Diego, CA 92121 USA; 14 Present Address: State Key Laboratory of Pharmaceutical Biotechnology, School of Life Sciences, Nanjing University, Nanjing, China; 15 Present Address: Engineering Research Center of Protein and Peptide Medicine, Ministry of Education, Nanjing, China; 16 Present Address: Denali Therapeutics, South San Francisco, CA USA; 17 Present Address: Department of Medicine Solna, Division of Immunology and Allergy, Karolinska Institutet and Karolinska University Hospital, Stockholm, Sweden; 18 Present Address: Department of Pharmacology, Northwestern University Feinberg School of Medicine, Chicago, IL 60611 USA; 19 Present Address: Bioscience COPD/IPF, Research and Early Development, Respiratory and Immunology, BioPharmaceuticals R&D, AstraZeneca, Cambridge, UK; 20 Present Address: Alchemab Therapeutics Ltd, Cambridge, UK; 21 Present Address: Evox Therapeutics Limited, Oxford Science Park, Medawar Centre, East Building, Robert Robinson Avenue, Oxford, OX4 4HG England

**Keywords:** Protein design, Integrins, Cryoelectron microscopy

## Abstract

The RGD (Arg-Gly-Asp)-binding integrins αvβ6 and αvβ8 are clinically validated cancer and fibrosis targets of considerable therapeutic importance. Compounds that can discriminate between homologous αvβ6 and αvβ8 and other RGD integrins, stabilize specific conformational states, and have high thermal stability could have considerable therapeutic utility. Existing small molecule and antibody inhibitors do not have all these properties, and hence new approaches are needed. Here we describe a generalized method for computationally designing RGD-containing miniproteins selective for a single RGD integrin heterodimer and conformational state. We design hyperstable, selective αvβ6 and αvβ8 inhibitors that bind with picomolar affinity. CryoEM structures of the designed inhibitor-integrin complexes are very close to the computational design models, and show that the inhibitors stabilize specific conformational states of the αvβ6 and the αvβ8 integrins. In a lung fibrosis mouse model, the αvβ6 inhibitor potently reduced fibrotic burden and improved overall lung mechanics, demonstrating the therapeutic potential of de novo designed integrin binding proteins with high selectivity.

## Abstract (web summary)

Roy et al. describe a generalized method for computationally designing miniproteins selective for a single integrin heterodimer and conformational state. The designed αvβ6 inhibitor remains monomeric and maintains biological activity following aerosolization and shows excellent efficacy in bleomycin induced lung fibrosis.

## Introduction

The highly homologous integrins αvβ6 and αvβ8 bind to latent transforming growth factor-β1 and β3 (L-TGF-β1 and L-TGF-β3) leading to release of active TGF-β1 and -β3^1–3^. Upregulation of αvβ6- and/or αvβ8-mediated TGF-β activation is a driver of multiple diseases, including idiopathic pulmonary fibrosis (IPF)^4–6^, primary sclerosing cholangitis (PSC)^7^, and several solid tumors^8–10^, but deconvoluting the contribution of αvβ6 and αvβ8 to the etiology of these diseases has been challenging due to limitations in current interventions. Selective antibodies targeting RGD integrins have been generated by immunizing mice^11–13^, but this approach lacks precise control over the target binding site on the integrin. Control over the target site is important because differential modulation of αvβ6 integrin conformations (bent-closed, extended-closed, and extended-open) by orthosteric and allosteric inhibitors has dramatically different outcomes on receptor internalization^11,14,15^ and has been linked to safety outcomes in preclinical and clinical studies^16^. For example, the mAb BG00011 and small molecule MORF-720 both target the non-internalized, bent-closed αvβ6 conformation^11,15^ and have on-target/αvβ6-mediated toxicity^17–19^, while the small molecules PLN-74809^20^ and GSK3008348^21^ stabilize the extended-open αvβ6 conformation that induces αvβ6 internalization, and have not shown any drug-related serious adverse events in clinical trials^22^. Since eight integrin heterodimers, including αvβ6 and αvβ8, share the conserved RGD binding sequence, it has not been possible to generate selective RGD-mimetic small molecules for individual integrins, making it challenging to dissect the role a single integrin plays in a particular disease^23^. Therefore, there is a need for a new integrin therapeutic modality with (i) high selectivity for a single RGD integrin heterodimer, (ii) atomic-level control over the precise location of the target binding site and the protein-protein interaction interfaces to control the evoked integrin conformation, (iii) hyperstability to enable tissue restricted administration (inhaled and oral), and (iv) a smaller hydrodynamic size than IgG antibodies to enable better tissue penetration.

Here, we describe a method for computationally designing hyperstable RGD-containing miniproteins that are highly selective for a single RGD integrin heterodimer and conformational state, and use this strategy to design inhibitors of αvβ6 and αvβ8 with high selectivity. In a mouse model of bleomycin-induced lung fibrosis, the αvβ6 inhibitor potently reduced fibrotic burden and improved overall lung mechanics when delivered via oropharyngeal administration mimicking inhalation, demonstrating the therapeutic potential of highly selective de novo designed integrin binding proteins.

## Results

### Computational design strategy

**Fig. 1:**
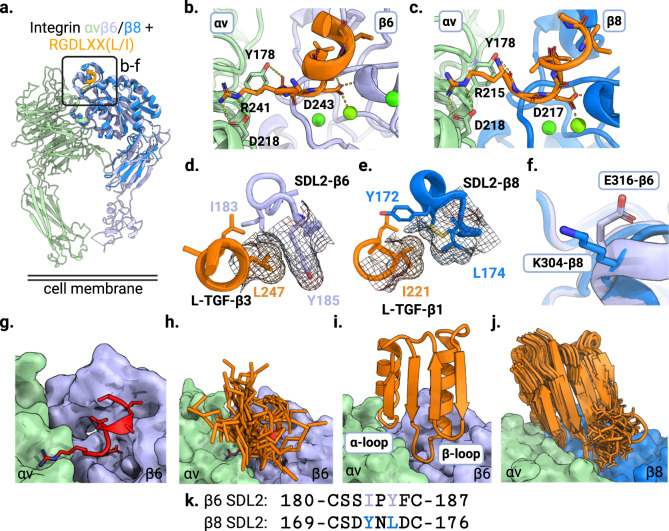
Computational design of αvβ6 and αvβ8 selective minibinders. **a** Crystal structure of β8 (PDB ID 6OM2) overlaid on the structure of αvβ6 integrin in complex with the L-TGF-β3 peptide RGDLXX(L/I) (PDB ID 4UM9). Inset highlights the zoomed-in regions shown in panels **b**–**f**. The αv subunit is shown in green, β6 is in lavender, β8 is in blue, and the RGDLXX(L/I) peptide is in orange. **b**, **c** Shared polar interactions between the RGD motif and (**b**) αvβ6 and (**c**) αvβ8 integrin (L-TGF-β3/αvβ6 complex PDB ID 4UM9, L-TGF-β1/αvβ8 PDB ID 6OM2). **d** Hydrophobic packing of LXX(L/I) motif of L-TGF-β3 peptide with SDL2 of αvβ6 (PDB ID 4UM9). Leu247 packs optimally against Y185 from SDL2 of β6. **e** Hydrophobic packing of LXX(L/I) of L-TGF-β1 peptide with SDL2 of αvβ8 (PDB ID 6OM2). I221 of L-TGF-β1 peptide packs less tightly against L174 on SDL2 of β8 compared to the homologous interactions in panel **d**. **f** Charge reversal on β subunit: β8 contains K304 whereas the equivalent position on β6 is E316. **g** Surface structure of the αvβ6 integrin in complex with L-TGF-β3 peptide (red cartoon representation, PDB ID 4UM9). **h** Low RMSD matches to the L-TGF-β3 peptide bound to αvβ6 were harvested from the PDB database (orange stick representations). **i** Non-clashing fragments with αvβ6 were then incorporated in the α/β ferredoxin folds (orange ribbon representation) using Rosetta. **j** Loop extension strategy to design an αvβ8 selective minibinder: to make more extensive contacts to the β8 subunit the β-loop was resampled by one residue insertion (blue surface representation for β8 subunit, PDB ID 6OM2). In addition to the loop extension, the LXX(L/I) motif was allowed to be redesigned using Rosetta. **k** Partial sequence alignment of SDL2 of the β6/β8 subunits is shown highlighting two key positions packing against the LXX(L/I) motif of the L-TGF-β ligand (I183 and Y185 in SLD2-β6, and Y172 and L174 in SDL2-β8).

We set out to overcome the limitations of integrin-targeted small molecules and antibodies by developing a computational approach that generates small (<75 amino acids) hyperstable de novo integrin binding proteins that have high integrin selectivity and specific receptor binding interfaces optimal for treating disease. Integrin αvβ6 and αvβ8 both bind to a RGDLXX(L/I) motif in the pro-domains of L-TGF-β1 and β3 with low nM affinity (Fig. 1a)^1,24^. As in other structures of RGD-containing peptides bound to integrins, the arginine and aspartate side chains make multiple hydrogen bond and salt-bridge interactions to residues at the interface between the integrin alpha and beta subunits (Fig. 1b, c). For both αvβ6 and αvβ8, C-terminal to the RGD, the peptide adopts an alpha-helix-like turn with two leucines (or Ile for β8) fitting into a hydrophobic pocket formed by a β6/β8 subunit specificity determining loop 2 (SDL2, Fig. 1d, e)^1,2,24^. In the unliganded state, SDL2 of αvβ6 is ordered with multiple backbone hydrogen bonds (PDB ID 4UM8), whereas SDL2 of unliganded αvβ8 is more flexible^1,2,24^. To engineer selectivity, we focused on two main areas on the β subunit that differ between the two targets: the region that contacts the LXX(L/I) motif in the L-TGF-β3 peptide (Fig. 1d, e) and a charge reversal on the β subunit (Fig. 1f). There are several key differences in the hydrophobic packing pattern of LXX(L/I) motif and SDL2 of β6 compared to β8 (Fig. 1d, e, k). Y185 from SDL2 of αvβ6 packs optimally with Leu (LXX(**L**/I), L247) of the L-TGF-β3 peptide (PDB ID 4UM9, Fig. 1d), while the equivalent position on the SDL2 of αvβ8 (L174) packs much less tightly with Ile (LXX(L/**I)**, I221) of L-TGF-β1 (PDB ID 6OM2, Fig. 1e). There is also a key charge reversal on the β subunit; β8 at position 304 has a lysine (K304) whereas the equivalent position on β6 is an glutamate (E316) (Fig. 1f). We hypothesized that minibinders interacting with the Y185/L174 and E316/K304 regions of αvβ6 and αvβ8 might be able to achieve selectivity between the two proteins.

To implement this design strategy, we sought to generate small proteins that incorporate the central RGD affinity loop, make favorable contacts with both α and β subunits, and interact closely with the two structurally diverging regions described above. We started from the crystal structure of human αvβ6 in complex with an RGD-containing L-TGF-β3 peptide (PDB ID 4UM9)^1^, and screened the PDB database in silico for topologies and structure segments capable of hosting the 8 residue extended turn conformation of the peptide (RGDLGALA, Fig. 1g). Low RMSD matches to the peptide backbone conformation, along with the five flanking residues on both the N- and C-termini, were superimposed on the bound peptide conformation in the complex structure, and those making backbone level clashes with the integrin were discarded (Fig. 1h). We found small α/β ferredoxin folds (Fig. 1i) were able to scaffold the RGDLXX(L/I) binding loop without clashing with the integrin while making additional contacts with both α and β subunit (α- and β-loop respectively, Fig. 1i, Supplementary Fig. 1). Structures were assembled from fragments following rules for constructing ideal proteins^25^, sampling different alpha helix, beta sheet, and loop lengths, while constraining torsion angles in the region corresponding to the RGD peptide to those observed in the co-crystal structure using Rosetta (Fig. 1g). Following two rounds of design and optimization (see supplementary info for details, Supplementary Figs. 2–4), two high affinity variants were selected for further characterization: B6B8_BP (av6_3_E13T) and B6_BP (av6_3_A39KG64R) (Supplementary Figs. 5 and 6). Affinity maturation identified a substitution to a lysine (A39K) making a salt-bridge interaction with E316 of the β6 subunit (Supplementary Fig. 5f) while the LXX(L/I) motif remained fixed confirming the importance of this motif for selectivity towards αvβ6 (Supplementary Fig. 5b).

**Fig. 2:**
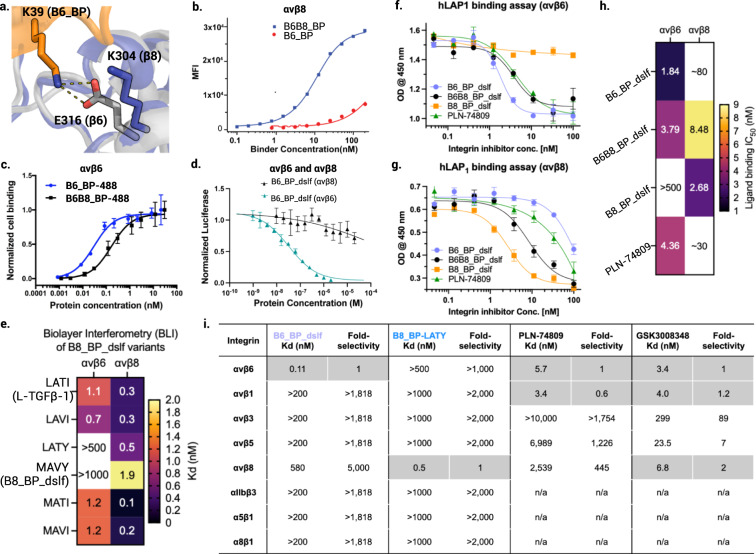
Selectivity of designed binders for αvβ6 and αvβ8. **a** The A39K mutation confers selectivity towards αvβ6 compared to αvβ8 where there is a charge reversal (Glu316 for β6 shown as a gray stick, Lys304 for β8 shown as a blue stick). **b** Cell surface titration of B6B8_BP and B6_BP against K562 cells stably transfected with αvβ8. B6B8_BP lacking the A39K mutation binds to αvβ8 with a Kd of ~7.3 nM whereas B6_BP containing the A39K mutation binds to αvβ8 > 500 nM. **c** Cell surface titration of AlexaFluor-488-labeled B6B8_BP and B6_BP using αvβ6 (+) human epidermoid A431 carcinoma cells. B6_BP binds to A431 cells with higher potency than B6B8_BP (30 (±0.004) pM vs 167 (±0.028) pM). Data are presented as mean values +/− SD (*n* = 3 independent experiments). **d** B6_BP_dslf selectively inhibits αvβ6-mediated TGF-β1 activation. αvβ6 and αvβ8 transfectants were co-incubated with CAGA-reporter cells and GARP/TGF-β1 transfectants and inhibitors. B6_BP_dslf inhibits TGF-β activation with an IC50 of 32.8 (±3.4) nM. Data are presented as mean values +/− SD. **e** Binding affinities (Kd) of B8_BP_dslf (MAVY) point mutants to integrins αvβ6 and αvβ8, determined by BLI. Each experiment was repeated at least twice (*n* = 2). The LATI motif is in native L-TGF-β1. **f**, **g** Competitive inhibition of h-LAP_1_ binding to (**f**) αvβ6 and (**g**) αvβ8 by designed inhibitors and control small molecule PLN-74809^20^. Each experiment was performed at least three times (*n* = 3). **h** Heatmap of IC_50_ values for h-LAP_1_ binding assays in **f** and **g**. **i** Binding affinities (Kd) and fold-selectivity values of B6_BP_dslf and B8_BP-LATY to all eight RGD integrins compared to small molecules PLN-74809^20^ and GSK3008348^21^. Binding data for PLN-74809 and GSK3008348 are taken from Decaris et al. 2021^20^. Rows shaded in gray indicate the RGD integrin(s) for which each molecule is selective (i.e B6_BP_dslf and B8_BP-LATY are both mono-selective whereas PLN-74809 and GSK3008348 are dual- and tri-selective, respectively). n/a not available.

To achieve selectivity for the β8 subunit, we redesigned the β-loop to take advantage of the K304 charge reversal on the β subunit (Fig. 1f). We generated 200 models with different lengths and conformations of the β-loop using RosettaRemodel^26^ and aligned them to the L-TGF-β1 / αvβ8 complex structure (PDB ID 6OM2) by superposition on the RGD peptide (Fig. 1j). As packing of the L-TGF-β1 LXX(L/I) motif with SDL2 of αvβ8 integrin is suboptimal (Fig. 1e), we hypothesized that a minibinder mimicking this interaction would be able to accommodate bulkier residues at these positions, giving additional selectivity. Both the loop extension and LXX(L/I) structural motif sequences were redesigned using Rosetta and the 9 designs with lowest predicted binding energy following structure prediction using AlphaFold^27^ were selected. Four out of 9 designs showed preferential binding to αvβ8 integrin with B8_BP_dslf showing the highest affinity and selectivity towards αvβ8 (Supplementary Fig. 7). Residues are critical for selectivity against αvβ8 vs. αvβ6. B8_BP_dslf is a monomeric and hyperstable protein when expressed in *E. coli* and binds to human αvβ8 with 1.9 nM affinity, with no appreciable binding to human αvβ6 up to 1 µM (Supplementary Fig. 6b. For the LXX(L/I) motif, B8_BP_dslf has the sequence MAVY which packs against SDL2 of αvβ8; in L-TGF-β1 the corresponding sequence is LATI. Replacement of MAVY with LATI (avb8_#12) on the yeast cell surface completely abrogated selectivity towards αvβ8 (Supplementary Fig. 7b), indicating these residues are critical for selectivity against αvβ8 vs. αvβ6. Purified avb8_12 with the reversion mutations loses selectivity towards αvβ8 and binds to αvβ6 with a Kd of 1.13 nM (Supplementary Table 1, Supplementary Fig. 6), confirming the importance of the LXX(L/I) motif for selectivity. We systematically varied each position within the LATI motif to determine which residue plays a critical role in determining selectivity (Fig. 2e). We found that B8_BP-LATY with a single substitution from I to Y bound to αvβ8 with an affinity of 500 pM with no appreciable binding to αvβ6 at 500 nM concentration (Fig. 2e, Supplementary Fig. 6b and Table 1).

### Selectivity profiles of αvβ6 and αvβ8 binders towards other RGD binding integrins

B6_BP and B8_BP_dslf are highly selective to αvβ6 and αvβ8, respectively. We investigated the selectivity of the designed binders against seven other RGD-binding integrins. B6B8_BP and B6_BP do not cross-react with RGD-binding integrins αvβ1, αvβ3, αvβ5, α5β1, α8β1, and αiibβ3 at concentrations up to 200 nM in cell surface binding experiments using K562 cells stably transfected with different RGD-binding integrins, corresponding to >1000-fold selectivity (Supplementary Fig. 8a, c). In B6_BP, the β-loop is positioned to confer selectivity between the two integrins, where residue K39 faces E316 on the β6 subunit and K304 on β8 (Fig. 2a). As intended, B6_BP is more selective for αvβ6 than B6B8_BP. B6_BP binds αvβ6 with a higher affinity than αvβ8 on the surface of K562 cells, with a *K*_*d*_ of 0.11 (±0.09) and 580 (±40) nM, respectively (Fig. 2b, Supplementary Fig. 8b). B6B8_BP, which has an alanine at this position (A39), is less selective for αvβ6, and binds to αvβ6 and αvβ8 with a *K*_*d*_ of 1.7 (±0.2) and 7.3 (±1.2) nM, respectively (Fig. 2b, Supplementary Fig. 8b). We generated fluorescently labeled B6B8_BP and B6_BP by conjugating AlexaFluor-488 to an engineered C-terminal cysteine via maleimide chemistry. The fluorescently labeled proteins were titrated against αvβ6 (+) human epidermoid carcinoma A431 cells. B6B8_BP and B6_BP bind to A431 cells with *K*_*d*_ values of 167 (±0.028) pM and 30 (±0.004) pM, respectively (Fig. 2c).

For the ease of aerosol formulation, we sought to increase the stability of the engineered inhibitors^28,29^. Four variants with additional disulfide bonds stapling the N and C terminus had considerably increased thermostability (Supplementary Fig. 9), and bound to αvβ6 with subnanomolar affinity; mutation of the RGD to KGE abrogated binding to αvβ6 confirming that the RGD loop is necessary for binding (Supplementary Fig. 6a). We further characterized one variant, B6_BP_dslf, and found that it selectively inhibited αvβ6-mediated TGF-β activation (IC50 32.8 ± 3.4 nM) using CAGA reporter cells^30^ and GARP/TGF-β1 transfectants, and had marginal effect on αvβ8-mediated TGF-β activation in the tested concentration range, confirming the selectivity towards αvβ6 (Fig. 2d, Supplementary Fig. 10d, e). αvβ8-selective B8_BP_dslf does not bind to any other RGD binding integrins as confirmed by BLI (Supplementary Fig. 6b).

We compared the potency and selectivity of our designed αvβ6 and αvβ8 minibinders to the small-molecule dual αvβ6/αvβ1 inhibitor (PLN-74809, Supplemental Data 6) currently in clinical trials as an oral IPF therapy^20^, by assessing their ability to outcompete binding of hLAP_1_, the endogenous ligand of αvβ6 and αvβ8. For αvβ6 integrin, B6_BP_dslf had the lowest IC_50_ (1.84 nM), followed by B6B8_BP_dslf (3.79 nM), PLN-74809 (4.36 nM); B8_BP_dslf had no detectable binding consistent with its very high selectivity (Fig. 2f, h). For αvβ8, B8_BP_dslf outcompeted hLAP_1_ with the lowest IC_50_ (2.68 nM), followed in order of potency by B6B8_BP_dslf (8.48 nM), PLN-74809, and B6_BP_dslf (Fig. 2g, h). Taken together, these data confirm that B6_BP_dslf and B8_BP_dslf have exquisite selectivity and affinity for their respective integrin targets, with considerably greater RGD integrin selectivity than the small molecules PLN-74809 and GSK3008348 (Fig. 2i).

### Negative stain EM reveals B6_BP_dslf stabilizes the αvβ6 open headpiece conformation

**Fig. 3:**
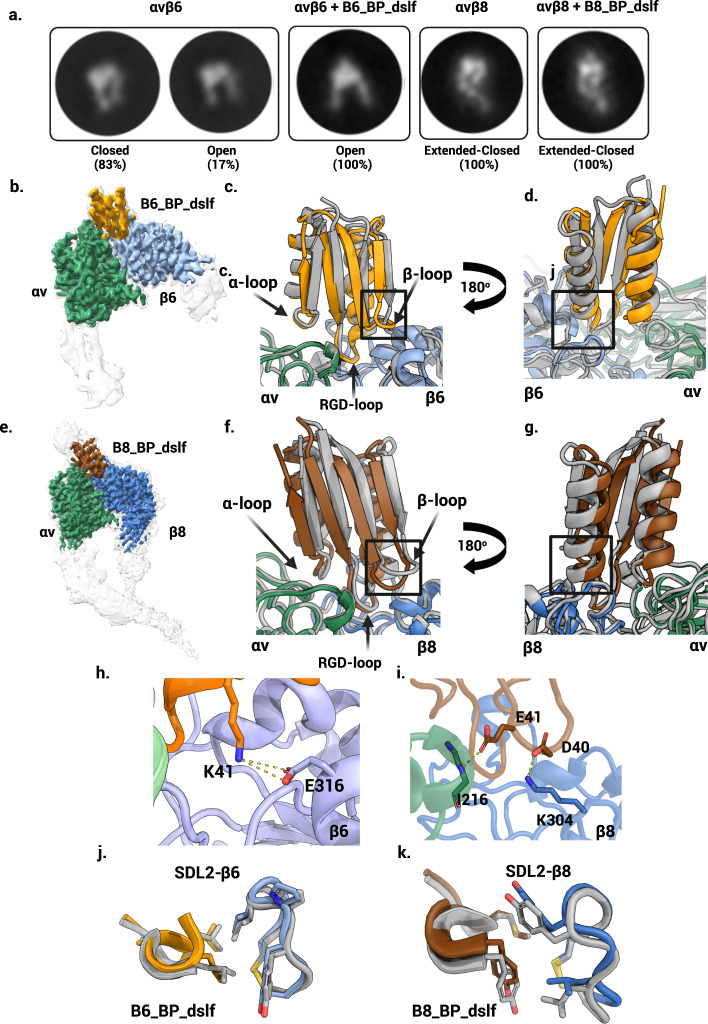
Structural characterization. **a** Representative 2D class averages of integrin with and without minibinder. For αvβ6, both closed and open headpiece conformations are present in the unbound state, but in the presence of the minibinder the open conformation is dominant. For αvβ8, no open headpieces were observed, with or without minibinder. **b** CryoEM density map of αvβ6 bound to minibinder B6_BP_dslf. B6_BP_dslf (goldenrod) binds the integrin ligand binding cleft between the αv (green) and β6 (light blue) subunits and induces or stabilizes the open conformation. The sharpened, locally refined cryoEM map is shown in color, superimposed with the unsharpened map showing all domains of the αvβ6 headpiece in semi-transparent white. **c**, **d** Overlay of the designed αvβ6 + B6_BP_dslf model (gray) and the experimentally determined cryoEM model (colors). Although the overall angle of the minibinder is shifted, the RGD loop positioning is as predicted. Insets in **c** and **d** are magnified in panels **h** and **i**, respectively. **e** CryoEM density map of αvβ8 bound to minibinder B8_BP_dslf. Similar to B6_BP_dslf, B8_BP_dslf (brown) binds the integrin ligand binding cleft between the αv (green) and β8 (blue) subunits, however the conformation of αvβ8 remains in the closed headpiece conformation. The sharpened, locally refined cryoEM map is shown in color, superimposed with the unsharpened map showing all domains in the αvβ8 ectodomain construct in semi-transparent white. **f**, **g** An overlay of the designed αvβ8 + B8_BP_dslf model (gray) and the experimentally determined model. Although the overall angle of the minibinder is shifted, the RGD loop positioning is as predicted. Insets in **f** and **g** are magnified in panels **i** and **k**, respectively. **h**, **i** Key designed interactions between β-loop and β6/β8 subunit are observed in the cryoEM structure: K41 from B6_BP_dslf forms a salt bridge with E316 from the β6 subunit (panel **h**). E41 from β-loop makes backbone level hydrogen bond with I216 from β8 subunit and D40 makes salt bridge interaction with K304 from β8 subunit (panel **i**). **j** Experimental vs designed (gray) packing pattern of the LXXL motif and SDL2 of αvβ6. **k** Experimental vs designed (gray) packing pattern of the MAVY motif and SDL2 of αvβ8.

Integrin αvβ6 adopts the well-characterized range of integrin conformations including bent-closed, extended-closed, and extended-open, which have been linked to activation and binding site accessibility^3^. However, αvβ8 has not been observed in this range of conformations and instead has been shown to bind and activate L-TGF-β while exclusively occupying the extended-closed conformation^2^. To investigate the effects of the binders on the αvβ6 conformational ensemble, we used single-particle negative stain electron microscopy to image complexes of minibinder binding on glycosylated soluble construct of the αvβ6 headpiece. All minibinder-integrin complexes were formed in a buffer containing excess Mn^2+^ ions to push the conformational equilibrium towards extended-open and to ensure the availability of the MIDAS cation, which is known to be crucial for ligand binding. As expected, the 2D class averages showed that both B6_BP_dslf and B8_BP_dslf bind at the canonical ligand binding site at the alpha/beta subunit cleft in αvβ6 and αvβ8, respectively (Fig. 3a). B6_BP_dslf induces αvβ6 headpiece opening whereas B8_BP_dslf does not have an effect on the global conformation of αvβ8 and the headpiece remains closed (Fig. 3a, Supplementary Fig. 18).

### cryoEM structure characterization

To investigate the accuracy of our designed structures, we used single-particle cryoelectron microscopy (cryoEM) to determine structures of human αvβ6 ectodomain bound to B6_BP_dslf and human αvβ8 ectodomain bound to B8_BP_dslf. Using focused refinement, the nominal overall resolution is 3.4 Å for the αvβ6 - B6_BP_dslf complex and 2.9 Å for the αvβ8 - B8_BP_dslf complex, although the resolution varies considerably due to the intrinsic flexibility of both integrins (Supplementary Data 6, Supplementary Fig. 19)^2^. Both integrin-minibinder complexes have extensive binding interfaces (Supplementary Data 1). The structure of the αvβ6 - B6_BP_dslf complex identifies several glycosylation sites that had not previously been observed structurally. Despite extensive 3D classification of the αvβ6 - B6_BP_dslf complex (Supplementary Fig. 19), we did not observe any subclasses of the integrin-minibinder complex in a closed headpiece conformation. As expected based on the negative stain class averages, the αvβ8 - B8_BP_dslf was found to be exclusively in the extended-closed conformation.

The secondary structural elements of the designed B6_BP_dslf minibinder model are in close agreement with the cryoEM map (complex RMSD 0.6 Å vs design, Fig. 3b–d) and the three minibinder loops make contact with the alpha, the beta, or both subunits of integrin, although some interactions vary slightly from the initial design (Supplementary Table 3). B6_BP_dslf was designed using a closed integrin headpiece (PDB ID 4UM9)^1^, however, our cryoEM map revealed that, when bound to minibinder, the βI domain of the β6 subunit rearranges to the same open conformation as when bound to ligand (Fig. 3b). In the cryoEM map, the overall orientation of the minibinder is shifted relative to initial design, but the RGD loop positioning is as predicted (Fig. 3c, d). As expected, the RGD loop spans the subunit binding interface with RGD-Arg10 forming a hydrogen bond with D218 of the αv-subunit and RGD-Asp12 of the minibinder in a position to coordinate with the MIDAS cation. The engineered positively charged, affinity-enhancing point mutation in B6_BP_dslf, A41K (A39K in B6_BP), interacts with negatively charged E316 of the β2 subunit to form a salt bridge (Fig. 3h). Although the second charge reversal mutation (G64R in B6_BP, G66R in B6_BP_dslf) does not form the anticipated salt bridge with D148 in our structure, we note that this binding surface has a strong negative charge and speculate that this stabilizes the positively charged Arg. As predicted, we observe hydrophobic packing of LXX(**L**/I) L16 with Y185 of the β6 subunit (Fig. 3j). Of the 13 interacting pairs of residues present in the cryoEM model, 11 are present in the computational design model, including all three salt bridges. The two unanticipated interactions were backbone interactions with integrin: minibinder R10 and αv A21 and minibinder RGD-Asp12 and β6 S127 (Supplementary Table 3).

The cryoEM model of αvβ8 - B8_BP_dslf complex is also very close to the computational design model (Fig. 3e–g, complex RMSD 0.7 Å). In the cryoEM model of the αvβ8 - B8_BP_dslf complex, there are 12 interacting pairs of residues between integrin and the minibinder (Supplementary Table 3); as in the design model, the α-loop interacts with αv, β-loop with β8, and the RGD loop spans the two subunits (Fig. 3f). Y172 of the β8-SDL2 loops bends inward to form a hydrophobic patch similar to the conformation in L-TGF-β-bound structures (Fig. 3k)^2^. The cryoEM structure reveals the molecular basis for Y in the fourth position of the LXX(L/I) motif (Fig. 2e): we find that Y16 forms stabilizing interactions with A115 of the β8 subunit and interacts with the less bulky L174 in the β8-SDL2 loop (Fig. 3k). The equivalent position in the β6-SDL2 loop, Y185, is more bulky and we hypothesize that the steric clash would interfere with binding.

### In vivo tumor targeting using fluorescently labeled B6_BP

**Fig. 4:**
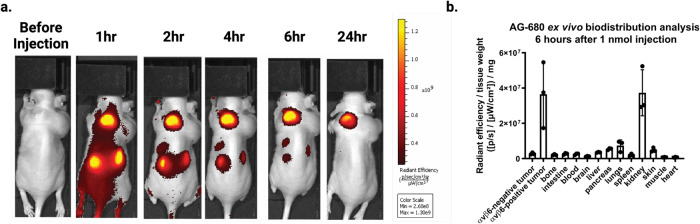
In vivo imaging of αvβ6 (+) A431 tumors using fluorescently labeled B6_BP. **a** Athymic nude mice were injected with αvβ6 (+) A431 cells on the left shoulder and αvβ6 (−) HEK293T cells on the right shoulder. AlexaFluor-680-labeled B6_BP (AF680-B6_BP) was injected via the tail vein to image the tumors over time as indicated (see Supplementary for additional images, *n* = 5). **b** Semiquantitative ex vivo biodistribution assay of AF-680-B6_BP at 6 h post-tail vein injection. B6_BP selectively accumulates in αvβ6 (+) tumors and primarily clears via glomerular filtration in the kidneys. Data are presented as mean values +/− SD, *n* = 5 mice.

As B6_BP binds to A431 cells with higher affinity and is more selective than B6B8_BP for αvβ6, we selected B6_BP for further in vivo experiments. We prepared tumor bearing rodents by injecting 6–8 week old female athymic nude mice with A431 cells (αvβ6 (+)) and HEK 293T (αvβ6 (−)) into the left and right shoulders, respectively. When the tumors reached 5–10 mm in diameter, mice were injected via the tail vein with 1.5 nmols of AlexaFluor-680 labeled B6_BP (AF680-B6_BP). AF680-B6_BP rapidly accumulated in the αvβ6 positive tumors and reached a high tumor-to-muscle fluorescence contrast ratio within 3 h post-injection (Fig. 4a, Supplementary Fig. 11). There was no detectable fluorescence at the αvβ6 negative HEK-293T tumors (Fig. 4a). We also performed a semiquantitative ex vivo biodistribution analysis of AF680-B6_BP at 6 h post-tail vein injection. Analysis of fluorescence intensities of different tissues revealed accumulation of AF680-B6_BP to αvβ6 positive tumors and kidney (tumor-to-kidney ratio 1:1.04) with no significant off-target binding including αvβ6 negative tumors (Fig. 4b). Quantification of whole body imaging data for AF680-B6_BP (Fig. 4b) suggests glomerular filtration through the kidneys into the urine is the primary route of elimination^31^. We also characterized the pharmacokinetics of B6_BP_dslf in the lungs and serum of healthy male C57BL/6 mice following a single dose via different routes of administration. B6_BP_dslf was rapidly cleared from the blood following IV and IP administration with a half-life of ~10 min, and following inhaled administration, B6_BP_dslf had a half-life in the lungs of ~1 h (Supplementary Fig. 12).

### In vivo efficacy of B6_BP_dslf in bleomycin-induced IPF

We investigated the therapeutic efficacy of B6_BP_dslf using the bleomycin-induced pulmonary fibrosis (PF) mouse model. 12-week-old C57BL/6 male mice were administered bleomycin intratracheally at 1 U/kg body weight to induce fibrosis in a “mild” and “severe” manner (see Methods for details) to mirror the progressive stages of PF. Longitudinal High-Resolution micro-Computed Tomography (HR-µCT) indicated fibrotic development at day 7 (Supplementary Figs. 13c and 15a). As proof-of-principle, we administered B6_BP_dslf intraperitoneally at 100 µg/kg in the mild bleomycin model and at 1 mg/kg in the severe bleomycin model starting on day 7 after bleomycin instillation and found significant improvement in lung health and function through HR-µCT and lung function measurements (Supplementary Figs. 13–15). For both doses, B6_BP_dslf halted fibrotic progression as evident by the reduced Ashcroft scores and improved respiratory mechanics such as static compliance and forced vital capacity (FVC) (Supplementary Figs. 13–15).

**Fig. 5:**
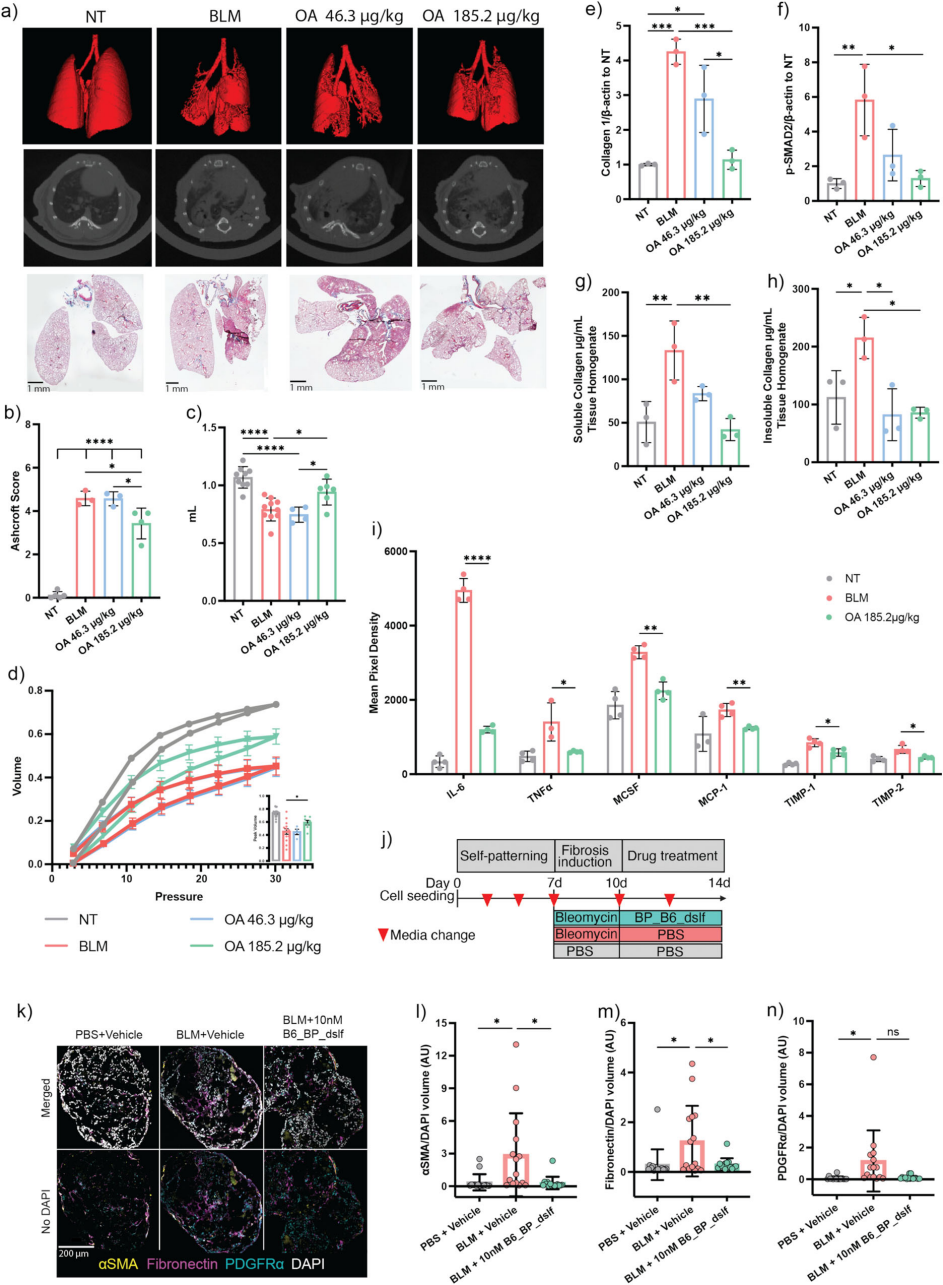
In vivo efficacy of OA-administered B6_BP_dslf in bleomycin-induced IPF. **a** Three dimensional renderings of HR-uCT scans (top panel), representative HR-uCT scans (middle panel), and representative Masson-trichrome images for nontreated (NT), bleomycin treated (BLM) and inhaled B6_BP_dslf groups. **b** Average Ashcroft Scoring of Masson-trichrome images (data represented as mean ± SEM, Tukey’s t-test, NT vs BLM *P* value = <0.0001, NT vs B6_BP_dslf 46.3 ug/kg *P* value = <0.0001, NT vs B6_BP_dslf 185.2 ug/kg *P* value = <0.0001, BLM vs B6_BP_dslf 185.2 ug/kg *P* value = 0.0214, B6_BP_dslf 46.3 ug/kg vs B6_BP_dslf 185.2 ug/kg *P* value = 0.0232). **c** Forced vital capacity as measured by SCIREQ flexiVent FX (data represented as mean ± SEM, Tukey’s t-test, NT vs BLM *P* value = <0.0001, NT vs B6_BP_dslf 46.3 ug/kg *P* value = <0.0001, BLM vs B6_BP_dslf 185.2 ug/kg *P* value = 0.034, B6_BP_dslf 46.3ug/kg vs B6_BP_dslf 185.2 ug/kg *P* value = 0.0386). **d** Pressure-Volume curves measured by SCIREQ flexiVent with peak volumes in the inset graph (data represented as mean ± SEM, Tukey’s t-test, BLM vs B6_BP_dslf 185.2 ug/kg *P* value = 0.0407). **e** Whole lung tissue homogenate western blot analysis of Collagen1 (data represented as mean ± SD, Tukey’s t-test, NT vs BLM *P* value = 0.0003, NT vs B6_BP_dslf 46.3 ug/kg *P* value = 0.0109, BLM vs B6_BP_dslf 185.2 ug/kg *P* value = 0.0005, B6_BP_dslf 46.3 ug/kg vs B6_BP_dslf 185.2 ug/kg *P* value = 0.0165) and **f** p-SMAD2 (data represented as mean ± SD, Tukey’s t-test, NT vs BLM *P* value = 0.0082, BLM vs B6_BP_dslf 185.2 ug/kg *P* value = 0.0117) show a dose-dependent reduction of these pro-fibrotic markers following B6_BP_dslf OA treatment. **g** Soluble (data represented as mean ± SD, Tukey’s t-test, NT vs BLM *P* value = 0.0078, BLM vs B6_BP_dslf 185.2 ug/kg *P* value = 0.0043) and **h** Insoluble (data represented as mean ± SD, Tukey’s t-test, NT vs BLM *P* value = 0.0382, BLM vs B6_BP_dslf 46.3 ug/kg *P* value = 0.01, BLM vs B6_BP_dslf 185.2 ug/kg *P* value = 0.0117) collagen levels are lower following B6_BP_dslf OA treatment. **i** Cytokine array analysis of common cytokines implicated in inflammation and IPF (all data represented as mean ± SD, all NT vs. BLM are significant, *p*-value < 0.05). **j** Time course of hFLO organoid growth, bleomycin induction, and 10 nM B6_BP_dslf treatment. **k** Fluorescent confocal microscopy imaging of hFLO sections immunostained with pro-fibrotic markers αSMA, fibronectin, and PDGFRα. (**l**–**n** are presented as bar graphs with mean ± SD, *N* = 15 cell aggregates were used per treatment, *P < 0.05 determined using two-tailed Welch’s t-test, box and whiskers show the mean and the maximum and minimum values) Volumetric analysis of (**l**) αSMA (PBS vs BLM *P* value = 0.0238, BLM vs BLM + 10 nM B6_BP_dslf *P* value = 0.0205) (**m**) fibronectin (PBS vs BLM *P* value = 0.0285, BLM vs BLM + 10 nM B6_BP_dslf *P* value = 0.0242, and **n** PDGFRα (PBS vs BLM *P* value = 0.0451) normalized to DAPI signal. *p-value < 0.05, **p-value < 0.01, ***p-value < 0.001, ****p-value < 0.0001.

A clinical trial involving an antibody targeting αvβ6 (BG00011) from Biogen has been discontinued because it exacerbated disease at higher doses among other serious adverse effects (SAE) including mortality^17^. The SAEs were attributed to increased alveolar inflammation, increased MMP12 generation, and emphysema due to the long half-life of BG00011^32,33^. An inhaled, tissue-restricted therapy delivered directly to the site of action of fibrosis in the lung could result in a considerably safer and more effective option than systemic inhibition of αvβ6-mediated TGF-β activation; therefore, we pursued a respiratory system delivery. To mimic inhalation^34^, mice were administered B6_BP_dslf via oropharyngeal administration (OA, 43.6 and 185.2 µg/kg) every other day starting at day 7 post-bleomycin instillation (using the severe bleomycin application method), ending on day 19, for a total of 7 treatments. Non-treated mice were given neither bleomycin nor B6_BP_dslf and serve as a healthy lung control to identify B6_BP_dslf efficacy. Three-dimensional renders and representative slices of the HR-µCT scans show increased healthy lung tissue available for segmentation in the 185.2 µg/kg B6_BP_dslf group compared to bleomycin controls (Fig. 5a, upper and middle panels). Quantification of HR-µCT scans shows a significant rescue of healthy lung volume following 185.2 µg/kg OA treatment and a shift away from fibrotic intensities (Supplementary Fig. 16a, b). Ashcroft scoring of Masson-trichrome stained lung sections by a blinded veterinary pathologist was significantly reduced in the 185.2 µg/kg B6_BP-disulf treatment group compared to the BLM and 43.62 µg/kg B6_BP-disulf treatment group (Fig. 5b). Western blot analysis of the whole lung homogenate lysates shows a reduction in TGF-β mechanistic biomarkers: collagen 1, pSMAD2, and fibronectin (Fig. 4e, f; Supplementary Fig. 16e, f). Further analysis of fibrosis using the Sircol™ Collagen Assay shows the 185.2 µg/kg OA treatment significantly attenuates both soluble and insoluble collagen deposition, indicative of newly synthesized collagen and more mature crosslinked collagen, respectively (Fig. 4g, h). FVC (Fig. 5c) and static compliance (Supplementary Fig. 16c) were significantly improved with 185.2 µg/kg OA treatment, and respiratory mechanics show a less restrictive nature (Fig. 5d). We investigated the 185.2 µg/kg dose further through recovered bronchoalveolar lavage fluid (BALF) using cytokine array analysis, histological immunofluorescence and Sirius Red staining. Commonly implicated cytokines in the progression and severity of PF noted in patients and upregulated in the bleomycin model of PF including IL-6, TNF-α, and TIMP-1, were significantly reduced in 185.2 µg/kg treated BALF samples (Fig. 5i)^35–38^. Immunofluorescence imaging shows a marked reduction in TGF-β-related fibrotic markers collagen type I and α-smooth muscle actin (α-SMA) (Supplementary Fig. 16d). Sirius Red staining corroborates a reduction of histological total collagen levels (Supplementary Fig. 16g).

With no significant changes in the total number of cells (Supplementary Fig. 16h–k) and reduction of inflammatory cytokines in BALF (Fig. 5i), inhaled B6_BP_dslf has potential to inhibit TGF-β induced fibrosis without exacerbating inflammation as compared to a systemically delivered antibody with a longer in vivo half-life^4^. A median mass aerodynamic diameter (MMAD) of ~1–5 µm is necessary to reach the lower respiratory tract^39^ for an inhaled nebulized drug. To confirm the aerosol formulability, we nebulized B6_BP_dslf using an Aeroneb nebulizer and collected aerosols of two different MMADs: 2.5–4 µm and 4–6 µm. For both of these particle sizes, B6_BP_dslf is monomeric, hyperstable, and binds to αvβ6 with similar affinity pre-nebulization (Supplementary Fig. 17), paving the way for the development of this molecule as an inhaled nebulized therapy for IPF.

### Evidence of human efficacy using lung organotypic model

To assess the viability of B6_BP_dslf in a human organoid-based bleomycin system, we used the human fluorescent lung alveolar organoid (hFLO) triculture model as described previously^40^. hFLO organoids were allowed to self-pattern for 7 days after which fibrosis was induced using bleomycin for 3 days prior to treatment with 10 nM B6_BP_dslf for 4 days. Upon immunofluorescent analysis, we observed increased pro-fibrotic markers α-SMA, fibronectin, and PDGFRα in bleomycin-treated organoids. Among these, we observed a statistically significant reduction in α-SMA and fibronectin levels in the organoids treated with B6_BP_dslf showing the efficacy of the treatment in lowering pro-fibrotic markers in human organoids (Fig. 5k–n).

## Discussion

The limited effectiveness of current treatments has renewed interest in developing inhaled therapeutics for IPF^34,41^. To the best of our knowledge, the designed αvβ6 inhibitor described here (B6_BP_dslf) binds to its target with higher affinity and selectivity than any previously reported linear or cyclic peptide, or disulfide cross-linked knottin inhibitors of αvβ6^42–46^, and is comparable to the leading antibody BG00011, which is no longer under development for IPF (Supplementary Table 2) due to exacerbation of the disease and death^17,19^. While inhaled tissue-restricted delivery of drugs at the site of action minimizes overall toxicity, dose, and adverse effects, a challenge in development of inhaled biologics is instability or aggregation at the liquid-air interface. The properties of B6_BP_dslf are unchanged following aerosolization: the protein is monomeric, thermostable, has the same CD spectrum, and binds to αvβ6 with similar affinity. In addition to the highly potent anti-fibrotic effects demonstrated here in the bleomycin-induced lung fibrosis model, the reduced systemic exposure due to the short serum half-life (~10 min), high selectivity and affinity for αvβ6, stabilization of the open αvβ6 conformation, ease of production using *E. coli*, hyper-thermostability, and aerosol formulatability give B6_BP_dslf an improved target product profile as a therapeutic candidate for IPF. Our designed αvβ6 inhibitor could also help combat progressive respiratory disease associated with current and future coronavirus infections^47–50^. Further improvement in the pharmacokinetic/pharmacodynamic properties of these molecules could likely be achieved by site-selective chemical PEGylation or fusion to an immunoglobulin Fc domain for immune-oncology indications^8,9^. More generally, a frequent challenge in drug development is the targeting of a single member within a large family of closely related proteins. This can be difficult to achieve with small molecules that share a conserved binding site, and the development of antibody panels capable of fine discrimination require considerable amounts of negative selection. Our structure-based de novo design strategy has high accuracy, as demonstrated by the cryoEM structures and achieves high selectivity by integrating both previously known binding motifs and introducing completely new interactions in a hyperstable small scaffold. As exemplified by our successful design of a potent and selective αvβ8 minibinder, this approach should be widely applicable to developing binders with high selectivity and affinity to individual members of the many therapeutically important families of cell surface receptors.

## Methods

All experiments were carried out in compliance with the appropriate and relevant ethical regulations. All animal protocols were reviewed and approved by an appropriate committee associated with the institution (see below for details). For the BLM induced PF model, all mice were housed in Brigham Young University’s pathogen-free facility and all experimentation was done in accordance with the protocol approved by the IACUC of Brigham Young University. Animal procedures were performed under the protocol #4470-01 approved by the Institutional Animal Care and Use Committee (IACUC) of University of Washington, Seattle, WA.

### Computational techniques

Overview of the design protocol has been discussed in the main text. Structures were assembled from fragments following rules for constructing ideal proteins^25^, sampling different alpha helix, beta sheet, and loop lengths, while constraining torsion angles in the region corresponding to the RGD peptide to those observed in the co-crystal structure using Rosetta. The resulting idealized ferredoxin fold structures were docked in complex with the αvβ6 integrin by superposition on the binding loop, and the amino acids at the binding surface were optimized for low energy interactions with the target. We solved the crystal structure of the best binder at 1.8 Å from the first round of design following a round of optimization using error prone PCR. The crystal structure fits the designed model well except for a rigid body translation of the C-terminal helix along the helical axis by one helical turn (~3.4 Å; Supplementary Fig. 1a).

In a second round of design, we docked the crystal structure of the Round 1 designed binder onto αvβ6 by superimposing on the RGD loop. We identified two loop regions in the design close to the integrin and sampled a range of lengths and conformations for the two loops, and selected 16 designs with loops predicted to make selective interactions with the integrin for experimental testing (Supplementary Figs. 1b, 2, and 3). Variant av6_3 with highest affinity towards αvβ6 was further subjected to site-saturation mutagenesis (SSM) (Supplementary Figs. 4 and 5) with increasing stringency. Five substitutions at the interface which primarily increase charge complementarity were enriched (Supplementary Fig. 5d–h). αvβ6 not only recognises the RGD loop but also an amphipathic helix formed by the LXXL motif that interacts only with the β6 subunit^1^, and substitutions in the region of the design corresponding to this motif were strongly disfavored for β6 binding (Supplementary Fig. 5b). We expressed and purified 9 enriched variants and measured binding for αvβ6 integrin using biolayer interferometry (BLI) measurements; all had subnanomolar binding affinity (the original av6_3 binds to αvβ6 with a *K*_*d*_ of 1.2 (±0.006) nM; Supplementary Fig. 6, Supplementary Table 1). Two high affinity variants were selected for further characterization: B6B8_BP (av6_3_E13T) with a single substitution and B6_BP (av6_3_A39KG64R) with two substitutions introducing positive charges complementing negative charges in both subunits of the αvβ6 integrin (Supplementary Figs. 5 and 6).

### Yeast display

Standard yeast surface display techniques were used to screen designs for binding and directed evolution. Genes encoding the designs were cloned into petcon2 in frame with N-term aga2 and C-term myc tag. Surface expression of myc was detected using FITC conjugated chicken anti-C-myc (Immunology Consultants Laboratory, Inc) and binding was detected using biotinylated human αvβ6 and stained with phycoerythrin conjugated streptavidin (Life technologies) for FACS. αvβ6 was chemically biotinylated to the lysine residues using EZ-Link Sulfo-NHS-LC-Biotin and biotinylation kits following manufacturer protocol. Excess biotin was removed from the mixture by dialyzing against a buffer containing no biotin. Two different buffers were used for the binding and washing steps for yeast display; Binding Buffer: 20 mM Tris, 150 mM NaCl, pH = 8.0, 1% BSA, 1 mM Ca^2+^ and 1 mM Mg^2+^, Wash Buffer: 20 mM Tris, 150 mM NaCl, pH = 8.0, 0.5% BSA, 1 mM Ca^2+^ and 1 mM Mg^2+^.

The SSM library was generated by using mutagenic primers (Supplementary Data 2, 3) for each position following a previously described protocol^51,52^. The resulting library was transformed into yeast using electroporation in duplicates (biological replicate). The sorting was performed in two rounds: The library was first treated with 4 µM Trypsin and 0.8 µM chymotrypsin^53^ for 45 s followed by labeling with 200 pM of biotinylated αvβ6 and top 5% of the binders were collected. For the second and final round of selection, 100 pM of biotinylated αvβ6 was used and the top 1% of the binding population was selected (Supplementary Fig. 4). DNA was extracted from pre and post sorted pools and barcoded. Enrichment ratios were calculated after sequencing the pools using Illumina. Sequences of all the variants of the designed minibinders are reported in Supplementary data 1.

### Protein minibinder expression and purification

Genes encoding protein variants were ordered as gblock gene fragments from IDT and cloned in pet29b in between NdeI/XhoI restriction sites with a C-term Histag, or directly ordered from IDT already cloned into pet29b. All the mutant variants of the proteins were expressed in BL21(DE3*) using Studier autoinduction technique in standard shake flasks at 37 *°*C for 24 h. Cells were harvested and resuspended in 20 mM Tris, 250 mM NaCl, 20 mM Imidazole (lysis buffer). Cells were lysed using microfluidizer and cell debris was separated by centrifuging at 24,000 g for 45 min. Soluble proteins were first purified using standard Ni-NTA affinity columns followed by size exclusion chromatography (S75 10/300 Increase) on a GE-Akta pure FPLC system. Peak corresponding to the monomeric protein was collected and further verified by mass spectrometry. For bleomycin induced PF models, protein was subjected to further purification to achieve endotoxin level <5 EU/ml.

### Integrin αvβ6 and αvβ8 DNA constructs

Wild-type human integrin αvβ6 headpiece^1^ and αvβ6 and αvβ8 ectodomains^11,54^ have been described. Here, the αvβ6 and αvβ8 headpiece and ectodomain constructs were synthesized by GenScript into the pCMV/R vector with an N-terminal signal peptide and C-terminal Human Rhinovirus (HRV) 3C protease cleavage site, ACID coiled coil in the αv (UniProt: P06756) subunit or a BASE coiled coil in the β6 (UniProt: P18564) and β8 (UniProt: P26012) subunits, and hexa-histidine tag. The αv, β6, and β8 subunits each had a cysteine mutation (M430GC, I287C, and V301C, respectively) to generate a disulfide bond to prevent α/β subunit dissociation following 3C cleavage. The Gly inserted prior to residue 430 in the M430GC mutation in the αv subunit was previously reported^55^. Plasmids were transformed into the NEB 5-alpha strain of *E. coli* (New England Biolabs) for subsequent DNA extraction from bacterial culture (Qiagen Plasmid Plus Maxi Kit) to obtain plasmid for transient transfection into Expi293F cells (Thermo Fisher Cat # A14527). The amino acid sequences for the αvβ6 and αvβ8 headpieces and ectodomains are listed in Supplementary data 4.

### Secreted integrin αvβ6 and αvβ8 expression and purification

For integrin headpieces, 800 mL cultures of Expi293F cells were grown in suspension to a density of 3.0 × 10^6^ cells per mL and transiently transfected using PEI-MAX (Polyscience) and cultivated for 5 days in Expi293F expression medium (Life Technologies) at 37 °C, 70% humidity, 8% CO2, and rotating at 150 rpm. Supernatants were clarified by centrifugation (5 min at 4000 rcf), PDADMAC solution was added to a final concentration of 0.0375% (Sigma Aldrich, #409014), and a final spin was performed (5 min at 4000 rcf). Clarified supernatant was supplemented with 1 M Tris-HCl pH 8.0 to a final concentration of 45 mM and 5 M NaCl to a final concentration of ~310 mM. His-tagged integrins were purified from clarified supernatants via a batch bind method where Ni Sepharose excel resin (Cytiva) was added to the treated supernatants and allowed to incubate overnight at 4 °C with gentle shaking. Resin was isolated using 0.2 μm vacuum filtration and transferred to a gravity column, where it was washed with 20 mM Tris pH 8.0, 300 mM NaCl, and protein was eluted with 3 column volumes of 20 mM Tris pH 8.0, 300 mM NaCl, 300 mM imidazole. Eluted protein was concentrated in 50 K MWCO centrifugal filters (Millipore), sterile filtered (0.22 μm), and applied to a Superdex 200 Increase 10/300 SEC column (Cytiva) using 20 mM Tris pH 8.0, 150 mM NaCl, 5% glycerol buffer on an AKTA Pure25 FPLC system (Cytiva). SDS-PAGE was used to assess purity and proper integrin dimerization.

For αvβ6 integrin headpiece and αvβ8 integrin ectodomain used in negative-stain or cryoEM analysis, the GenScript plasmids (described above) containing either the αv and β6 headpieces (αvβ6) or the αv and β8 ectodomains (αvβ8) were co-transfected into ExpiCHO cells (ThermoFisher, Cat #A29127) and grown per the manufacturer’s ‘Max Titer’ recommendations. In brief, cells were grown in suspension at 37 °C, 8% CO_2_, and ~90% humidity. Cultures were co-transfected with plasmids encoding an alpha and beta subunit with Expifectamine CHO. One day post-transfection cells were supplemented with Enhancer and Feed, and cultures were then moved to a 32 °C, 5% CO_2_ incubator. Five days post-transfection, supernatant was harvested and clarified via centrifugation before affinity purification using a 5 mL HisTrap FF Crude column (Cytiva). Eluted protein was pooled, concentrated and purified via gel filtration chromatography using a Superdex 200 Increase 10/300 SEC column (Cytiva) that had been equilibrated with 20 mM Tris-HCl pH = 7.4, 150 mM NaCl, 1 mM MgCl_2,_ and 1 mM CaCl_2_. Peak fractions were pooled, concentrated, and incubated overnight at 4 °C with 1:20 3 C PreScission protease to cleave the ACID-BASE coils. The following day glycerol (10% v/v) was added and samples were snap-frozen and stored at −80 °C.

### Synthesis of PLN-74809

PLN-74809 was identified as Compound 5 in a patent application from Pliant Therapeutics^56^ (Supplementary Data 5) and was synthesized to >97.5% HPLC purity by WuXi STA (Shanghai, China).

### Biotinylation of designed proteins

To generate mono-biotinylated proteins, avi-tag sequence (GLNDIFEAQKIEWHE) was introduced to the N-term of the proteins. Proteins were biotinylated either by co-transforming protein of interest along with pBirA, a vector encoding *E.coli* biotin ligase for in vivo biotinylation or using purified protein and an in vitro biotinylation kit from Avity using manufacturer’s protocol. Biotinylation was further confirmed via mass spec.

### Structural analysis of designed proteins

For determining the crystal structure of B6B8_BP_dslf, we expressed B6B8_BP_dslf with a N- terminal TEV cleavable histag. After protein expression and purification, B6B8_BP_dslf was treated with (1/100) dilution of stock TEV protease and incubated overnight at room temperature dialyzing against TBS. Following the completion of the cleavage (as monitored via SDS-page gel), proteins were run over a second gravity Ni-NTA column to separate cut his-tag and his-tagged-TEV from cleaved protein.

Following the his-tag cleavage, protein was concentrated to ~50 mg/ml and subjected to crystallization trials. Both Binding protein and B6B8_BP_dslf were crystallized by vapor diffusion at 24 °C by mixing with an equal volume of reservoir solution: 0.2 M KNO_3_, 20% PEG3350 (Binding protein) and 0.2 M tripotassium citrate, 20% PEG3350 (B6B8_BP_dslf). Crystals were briefly cryo-soaked in a reservoir solution containing 15% PEG200 and flash-frozen in liquid nitrogen. Diffraction data were collected at the GM/CA beam line of Advanced Photon Source (APS) at −173 °C using a MAR225 CCD detector and processed using XDS.

The diffraction data for binding protein were originally scaled to P6_1_22 space group with large Patterson peaks 1/3 and 2/3 along the c axis indicating two translational NCS molecules along the c axis. A solution was found using molecular replacement with the designed model. Autobuild was able to rebuild most sequences in the model, but R and Rfree were still very high, at 44%/47% with reasonably good electron density maps. Data were then re-scaled to the P3_1_ space group with 12 molecules per asymmetric unit and refined with tetrahedral twinning with three twin laws: -k, -h, -l; k, h, -l; and -h, -k, l. AUTOBUILD was used to build one-third of the sequence and was used several times in the first few of many iterative steps of manual building in COOT^57^ and refinement with PHENIX and RefMAC. MolProbity^58^ was used to validate the final structure.

### Negative-stain EM sample preparation

The integrin-minibinder complexes were formed using a 1:2 integrin to minibinder ratio, incubated at room temperature for 60 min, and diluted to a final concentration of 10 µg/mL in 20 mM Tris-HCl pH = 7.4, 150 mM NaCl, 1 mM MgCl_2,_ and 1 mM CaCl_2_. For both experiments, 3 µL of sample was applied to a glow-discharged 400 mesh copper glider grid that had been covered with a thin layer of continuous amorphous carbon. The specimens were stained with a solution containing 2% (wt/vol) uranyl formate as previously described (PMCID: PMC389902).

### Negative-stain EM data acquisition and processing

Data were acquired using a Thermo Fisher Scientific Talos L120C transmission electron microscope operating at 200 kV and recorded on a 4k × 4k Thermo Fisher Scientific Ceta camera at a nominal magnification of 92,000× with a pixel size of 0.158 nm. Leginon^59^ was used to collect 296 (αvβ6) or 337 (αvβ8) micrographs at a nominal range of 1.8–2.2 μm under focus and a dose of approximately 50 e^−^/Å^2^.

Experimental data were processed using cryoSPARC^60^ and CTFFIND4^61^ within the cryoSPARC wrapper. Initially, 34,794 (αvβ6) or 36,710 (αvβ8) particles were picked using an unbiased blob picker and subjected to three rounds of reference-free 2D alignment and classification to remove false positive particle images. The final particle counts contributing to 2D class averages were 22,096 (αvβ6) and 24,532 (αvβ8).

### CryoEM sample preparation

The integrin-minibinder complexes were formed using a 1:2 integrin to minibinder ratio, incubated at room temperature for 60 min, subjected to size exclusion chromatography, and concentrated to 2-3 mg/mL. From there, complexes were diluted to a final concentration of 0.90 mg/mL (αvβ6) or 0.96 mg/mL (αvβ8) in 20 mM Tris-HCl pH = 7.4, 150 mM NaCl, 1 mM MgCl_2,_ and 1 mM CaCl_2_. For cryoEM grid preparation, both Quantafoil and UltrAufoil grids were glow-discharged for 60 s at 15 mA. Just prior to sample application, 10% CHAPS detergent was added to each complex up to a final concentration of 0.025%. From there, 3 µL of each complex was added to each grid. Both complexes were frozen with a Thermo Fisher Scientific Vitrobot Mark IV using a 4 s blot time (αvβ6) or 4 s and 10 s blot times (αvβ8). All grids were frozen with 100% humidity at 4 °C and plunge-frozen in liquid ethane cooled by liquid nitrogen.

### CryoEM data acquisition and processing

Four datasets (two for αvβ6, three for αvβ8) were acquired on a Thermo Fisher Scientific Glacios cryo-transmission electron microscope operating at 200 kV and recorded with a Gatan K3 Direct Detection Camera. Automated data collection was carried out using the SerialEM software^62^. Ninty-nine frame movies were recorded in super-resolution mode with a super-resolution pixel size of 0.561 Å/px and a nominal magnification of 36kx. Each dataset was collected in a single session with a nominal defocus range of 1.0–1.8 μm under focus and a dose of approximately 50 e^−^/Å^2^. For the αvβ6 complex, one dataset of 1152 micrographs was collected from an Quantafoil grid (gold, 300 mesh, 1.2/1.3) without stage tilt to and a second of 722 micrographs was collected from an UltrAufoil grid with the stage tilted to 35°. For the αvβ8 complex, three datasets were collected from a Quantafoil grid (gold, 300 mesh, 1.2/1.3) with a stage tilt of 0° (1460 micrographs) or a single UltrAufoil grid with the stage tilted to of 30° (570 micrographs) or 35° (3174 micrographs).

Dose fractionated super-resolution image stacks were motion corrected and binned 2 × 2 by Fourier cropping using MotionCor2^63^. Motion corrected stacks were then processed using cryoSPARC^60^ and CTFFIND4^61^ within the cryoSPARC wrapper. Initially, 360,600 (αvβ6) or 3,325,476 (αvβ8) particles were picked using the unbiased blob picker in cryoSPARC and subjected to multiple rounds of reference-free 2D and 3D alignment and classification as outlined in Supplementary Fig. 19. The particle counts contributing to the final 3D structures into which the models were built are 124,715 (αvβ6) and 116,587 (αvβ8). Images showing EM maps were generated using UCSF Chimera, UCSF ChimeraX^64^.

### Model building

The initial atomic models used for the αvβ6 - minibinder complex were the αvβ6 headpiece (PDB: 5FFO, chains E and F) and the designed B6_BP_dslf model. The initial models used for the αvβ8 - minibinder complex were αvβ8 (PDB: 6UJA) and the designed B8_BP2_dslf model. Initial models were fit into their respective cryoEM density using UCSF Chimera^65^ and manually adjusted in COOT^66^. Glycans were manually added using COOT. Models (including glycans) were refined and relaxed using Rosetta. Modeling was aided by using cryoEM maps that were focused on specific regions, using sharpened and unsharpened maps. All maps used for modeling have been deposited.

### Biophysical characterization of designed proteins

Protein secondary structure and thermal stability were measured using the JASCO-1500 CD instrument. For wavelength scan, 10–15 µM of protein in TBS (20 mM Tris, 50 mM NaCl, pH = 8.0) was used. The CD spectra were measured from 240 to 195 nm with a scan rate of 100 nm/min. For thermal melt experiments, signal intensity at 222 nm was monitored as a function of temperature (4–95 °C) with a temperature gradient of 2 °C/min. The sample was held at the specified temperature for at least 5 s before the measurement. To investigate the role of the engineered disulfide bond on stability, 1 mM TCEP was added to the protein to measure thermal stability under reducing conditions.

### Nebulization of B6_BP_dslf

For stability studies, 6 mL protein solution was added to the reservoir of an Aeroneb nebulizer (Kent Scientific item #AG-AL7000 and either item #AG-AL1000 [4.0–6.0 um VMD) or item #AG-AL1100 [2.5–4.0 um VMD]). The solution was nebulized for approximately 10 min while collecting any condensate in a 50 mL Falcon tube placed in a dry ice and ethanol bath (dry ice and ethanol facilitate deposition of the aerosol). Condensate in the Falcon tube and any residual volume remaining in the nebulizer reservoir were both collected and analyzed for stability and activity. Procedure was subsequently repeated using 6 mL of a 1:10 protein solution diluted in PBS.

### Biolayer interferometry for determining binding kinetics of minibinder proteins

Data was collected on an Octet RED96 (Forte Bio) and processed using the instrument’s software. His Tagged and avi-tagged protein binders were immobilized on Ni-NTA and streptavidin sensor tips. The tips were then dipped into wells containing different concentrations of αvβ6 and αvβ8. Association and dissociation steps were recorded for 900 s and 1200 s respectively. An empty sensor with no loaded binding protein was included to discard any non-specific binding of αvβ6 to the octet tip.

### Fluorescent labeling of designed binders

For in vitro binding assay and in vivo imaging experiments, designed binders were labeled with Alexa Fluor™ 488 C5 Maleimide and Alexa Fluor™ 680 C2 Maleimide (Thermo Fisher Scientific), respectively, via a C-term single cysteine variant. In a typical labeling experiment, 50–200 µM of proteins were reduced with 1 mM TCEP for 30 min at room temperature. 3–5 molar excess of the maleimides were added to the protein solution and tumbled at room temperature overnight. The reaction mixture was then purified on a S75 Increase 10/300 column (GE healthcare) to separate free dye from the labeled proteins. Fluorophore conjugation was further confirmed by mass spectrometry.

### In vitro and in vivo binding assays using fluorescently labeled binders

Epidermoid cancer cells (A431) and human embryonic kidney 293T cells (HEK 293T) were purchased from American Type Culture Collection (ATCC, product # CRL-1555 and CRL-3216) and grown in Dulbecco’s Modified Eagle Medium (DMEM, Gibco) supplemented with 10% fetal bovine serum (FBS, Gibco) in a humidified atmosphere with 5% CO2 at 37 °C. Binding assays were performed on A431 carcinoma cells. A431 cells were dissociated from culture flasks with enzyme-free cell dissociation buffer (Gibco). Varying concentrations of AG-AF488 and E13T-AF488 were incubated with 5 × 10^4^ A431 cells in 1X TBS with 0.1% BSA, 1 mM Ca^2+^, and 1 mM Mg^2+^ (BTBS) rotating in suspension for 5 h at 4 °C. Sufficient incubation volumes were used to avoid >5% ligand depletion. After incubation, cells were washed with BTBS and analyzed by flow cytometry on a Accuri C6 instrument (BD Biosciences) or Invitrogen Attune NxT (Invitrogen), and data were quantified using FlowJo software (TreeStar). *K*_*d*_ values were determined by fitting the data to a one site– specific binding curve using Prism 7 (GraphPad Software).

For in vivo imaging experiments; approximately 1.5 × 10^7^ A431 cells and 1.5 × 10^7^ HEK 293T cells were suspended in 25 μL DMEM supplemented with 10% FBS along with 25 μL Matrigel Basement Membrane Matrix (Corning, #354234) and injected into the left and right shoulder, respectively, of 6–8 week old female athymic nude mice (Jackson Laboratories, NU/J #002019, homozygous for Foxn1nu). Mice were imaged when tumors reached 5–10 mm in diameter. Tumor Bearing mice were injected via the tail vein with 1.5 nmol AF680-labeled proteins in 100 μL of 20 mM Tris buffer (pH 7.4) and 150 mM NaCl, and imaged with a IVIS Lumina Series III system (PerkinElmer) at the indicated time points. The AF680 fluorophore was excited at 615–665 nm and emission was analyzed at 695–770 nm. In each image, a mouse injected with PBS alone was included as a negative control to allow measurement of background signals for data processing.

### Inhibition of αvβ6-mediated TGF-β activation by B6_BP_dslf using a TMLC co-culture assay

TGF-β activation was measured using transformed mink lung epithelial cells (TMLC) stably transfected with part of the plasminogen activated inhibitor 1 (PAI-1) reporter conjugated to a luciferase reporter (from Professor Daniel Rifkin (New York University, NY, USA))^67^. TMLCs were plated in 96 well plates (15,000 cells/well) in DMEM (Gibco 41966) supplemented with 1% FBS, 10 U/ml Penicillin G and 10 ug/ml Streptomycin G sulfate and allowed to adhere for 3 h. K562 cells stably transfected with αvβ6^68^ were incubated with B6B8_BP, B6_BP, or media alone (‘no BP’ control) or a range of control antibodies for 15 min to allow binding to occur, and then co-cultured with the TMLCs overnight (final concentration 60,000 αvβ6 K562 cells/well). TMLCs incubated alone or co-cultured with parental K562 cells were included as negative controls. Recombinant TGF-β1 (1 ng/ml R&D Systems) was included as a positive control for the PAI-1 luciferase reporter response to active TGF-β1, and a number of antibodies were included in the TMLC:αvβ6 K562 co-culture as additional controls. Anti-TGFβ1, 2, 3 mIgG1 clone 1D11 (20 ug/ml) was included to confirm the effect of complete inhibition of TGF-β1 activity in this assay, along with the corresponding mouse IgG1 isotype control (both antibodies R & D Systems). A titration of anti-avβ6 3G9 hIgG1 monoclonal antibody (1.4–1000 ng/ml, known clinically as STX-100, formerly known as 6.3G9^11^) was included as a positive control for inhibition of avβ6 in this assay along with a human IgG1 isotype control. Additional antibodies were included at single (high) concentrations to indicate the effect of maximal inhibition with each antibody. 264RAD (40 μg/ml) was included as this is a neutralizing antibody to both avβ6 and avβ8^68^. An anti-αv (CD51) antibody (20 mg/ml, Enzo Life Sciences) was included to confirm the effect of inhibiting multiple integrins containing the αvsubunit. Unless indicated otherwise, antibodies were manufactured by AstraZeneca, UK. After 18–20 h, the supernatants were removed and cells were washed in PBS and then lysed in 100 µl of reporter lysis buffer (1x) (Promega E397A) followed by a freeze thaw cycle to ensure complete lysis. Luciferase activity was quantified using the Luciferase assay system (Promega E1501) according to the manufacturer’s instructions. 80 µl of lysate was transferred to white-walled luminescence plates (Perkin Elmer), followed by addition of 100 µl of Luciferase assay reagent and the luminescence signal read on a luminometer (Envision, Perkin Elmer).

### Competitive inhibition of αvβ6- and αvβ8-mediated TGF-β activation by B6_BP_dslf using a CAGA co-culture assay

TGF-β activation was measured using HEK293 cells stably transfected with a reporter construct consisting of 12 repeats of the CAGA TGF-β responsive element upstream of the luciferase gene (from Professor Tom Thompson (University of Cincinnati, Cincinnati, OH, USA)^69^. Expi293F cells were transfected with αvβ6, αvβ8, or empty vector, or co-transfected with GARP and TGF-β1 with an N-terminal FLAG tag. After two days in FreeStyle culture medium, cells were harvested for the co-culture assay. Cell surface expression of αvβ6 and αvβ8 was confirmed by staining with integrin αv subunit-specific 17E6 antibody^70^ (20 ug/mL) followed by secondary fluorescent detection in FACS using AlexaFluor647 goat anti-mouse (Invitrogen A21235, at 800-fold dilution).

Cell surface expression of GARP/TGF-β1 complexes was confirmed by direct fluorescent detection of APC-labeled anti-FLAG (clone L5, BioLegend) 637308) staining in FACS. CAGA-reporter cells (15,000 cells) were plated with αvβ6 or αvβ8 EXPi293F transient transfectants (15,000 cells) in 96 well plates (30,000 cells/well) in FreeStyle (Gibco). Expi293F cells transiently co-transfected with GARP and TGF-β1 (5000 cells) and empty vector transfected cells (10,000) were mixed with 2-fold serial dilutions of B6_BP_dslf, irrelevant nanobody, inhibitory 7.1g10 antibody (integrin β6 subunit specific)^11^, or inhibitory ADWA11 antibody (integrin β8 subunit specific)^13^ in FreeStyle media and then immediately co-cultured with the CAGA-reporter cells and integrin transfectants overnight (for a final total amount of 45,000 cells/well in 100 µl of media). Serial dilutions were prepared in Freestyle media supplemented with 0.1% BSA to prevent protein loss due to adherence to plastic. CAGA-reporter cells co-cultured with GARP/TGF-β1 co-transfectants and empty vector (mock) transfectants were included to determine the background level of integrin-independent TGF-β1 activation, and CAGA-reporter cells co-cultured with GARP/TGF-β1 co-transfectants and αvβ6 or αvβ8 transfectants were used to determine the total level of TGF-β1 activation in the presence of either integrin. After 18–20 h, the supernatants were removed and cells were incubated in 50 μl of reporter lysis buffer (1x) (Promega E397A) on ice for 30 min. Luciferase activity was quantified using the Luciferase assay system (Promega E1501) according to the manufacturer’s instructions. 40 µl of lysate was transferred to white-walled luminescence plates (Perkin Elmer), followed by addition of 40 µl of Luciferase assay reagent and the luminescence signal read on a luminometer (Biotek Synergy H1).

### Human integrin αvβ6 and αvβ8 Latency Associated Peptide-1 (hLAP_1_)-binding assays

Recombinant human integrin αvβ6 or αvβ8 headpieces at 1.0 μg/mL in TBS (25 mM Tris pH 8.0, 150 mM NaCl) were incubated overnight at 4 °C on 96-well Nunc MaxiSorp plates (ThermoFisher, #442404) (100 μL/well). Plates were blocked with 200 μL/well of blocking buffer (2% [w/v] bovine serum albumin [BSA] in TBS) and incubated for 1 h at 37 °C. Plates were washed 3× in binding/wash buffer (25 mM Tris pH 8.0, 150 mM NaCl, 1 mM CaCl_2_, 1 mM MgCl_2_, 1 mM MnCl_2_, 0.1% [w/v] BSA, 0.02% [v/v] Tween20) using a robotic plate washer (BioTek). Serial dilutions of B6_BP_dslf, B6B8_BP_dslf, B8_BP_dslf, and PLN-74809 starting at 100 nM and diluting 3-fold seven times and 1 μg/mL (for αvβ6) or 0.25 μg/mL (for αvβ8) recombinant human LAP_1_ (R&D Systems, #246-LP-025/CF) in binding/wash buffer were added (100 μL/well) and incubated for 2 h at room temperature. After washing plates 3× in binding/wash buffer, plates were incubated for 1 h at room temperature with 0.5 μg/mL biotinylated mouse IgG2a anti-hLAP_1_ antibody in binding/wash buffer (R&D Systems, clone #27240, #BAM2462) (100 μL/well). Following washing plates 3× in binding/wash buffer, plates were incubated with peroxidase-conjugated streptavidin (Vector Labs, #SA-5014-1) in binding/wash buffer for 30 min at room temperature (100 μL/well). Following a final 3× plate wash in binding/wash buffer, 100 μL TMB (3,3′,5′,5-tetramethylbenzidine) substrate (SeraCare, #5120-0083) was added to each well, and then TMB was immediately quenched with 1 N HCl (100 μL/well). Absorbance at 450 nm was immediately collected for each well on an Agilent BioTek Epoch 2 plate reader. Data were plotted in Prism 9 (GraphPad Software, San Diego, CA) to determine the 50% inhibitory concentration (IC_50_) using a four-parameter logistic regression model.

### Animals

For the BLM induced PF model, all mice were housed in Brigham Young University’s pathogen-free facility and all experimentation was done in accordance with the protocol approved by the IACUC of Brigham Young University. These protocols conform to both institutional and national guidelines and regulations. Mice were randomly assigned to the treatment groups before any experimental assessments were made. Each cage had a combination of treatment groups to control for cage variation.

For pharmacokinetic analysis of B6_BP_dslf following different routes of administration, male C57BL/6 mice (8–12 weeks old) were obtained from Jackson Laboratory (Strain# 000664, Bar Harbor, Maine) and maintained at the Comparative Medicine Facility at the University of Washington, Seattle, WA, accredited by the American Association for the Accreditation of Laboratory Animal Care International (AAALAC). Animal procedures were performed under the protocol #4470-01 approved by the Institutional Animal Care and Use Committee (IACUC) of University of Washington, Seattle, WA.

### Pharmacokinetic analysis of B6_BP_dslf in healthy mice

Pharmacokinetics of B6_BP_dslf via the intravenous (IV), intraperitoneal (IP), and intranasal (IN) routes was investigated in healthy male C57BL/6 mice (*N* = 5–7 per time point, body weight 19–22 g) housed in standard holding cages and maintained in a controlled environment with free access to food and water in the Comparative Medicine Facility at the University of Washington, Seattle, WA. B6_BP_dslf, dissolved in 25 mM Tris pH 8.0, 150 mM NaCl, was dosed under isoflurane anesthesia by the IV (via retro-orbital injection) and IP routes at 2 mg/kg and via the IN route at 4 mg/kg. For IV and IP routes, a fixed volume of 50 μL was used. For IN administration, mice were lightly scruffed to create a vertical line from the nose to the lung and a fixed volume of 20 μL (10 μL/nostril) was dripped into the nostrils and allowed to be inhaled. Animals were returned to their home cage and propped up on their back to recover. Lungs were perfused with ~15 mL TBS via the right heart ventricle and harvested and blotted dry on a time course of 5 min to 48 h after B6_BP_dslf administration; lung weight was recorded and lung was flash-frozen in liquid nitrogen and stored at −80 °C until homogenization. Blood samples were also obtained over the same time course via sampling by terminal cardiac puncture. Serum was isolated from hematocrit via centrifugation at 2000 × *g* for 10 min, and stored at −80 °C until use. Non-compartmental methods were used to obtain estimates of the terminal elimination half-life (t_1/2,z_) for each animal and mean values were obtained by averaging the individual parameters.

### Bleomycin-induced pulmonary fibrosis

12 week-old C57BL/6 male mice (Jackson Laboratories, Bar Harbor, ME) were intratracheally instilled with a single dose of 1 U/kg bleomycin sulfate in 0.9% sterile saline (50 µl/animal). Mice treated with the intraperitoneal injection were anesthetized with an intraperitoneal injection of ketamine-xylazine (100 and 10 mg/kg body weight) in 0.9% sterile saline. An IV Catheter (BD Insyte shielded IV catheter, 22 ga × 38 mm) was inserted into the trachea. Bleomycin was then administered through the catheter. Using this method, we observed limited fibrotic development (i.e., “mild” bleomycin model), and so for the inhalation study, we changed our method as advised by our IACUC committee to use isoflurane to anesthetize and added the application of approximately 10 µL of 2% lidocaine to the laryngeal area using the tip of a feeding tube (20 ga × 38 mm, Instech Lab Inc.) to inhibit spontaneous closing of the vocal cords. This improved the development of fibrosis, and we refer to this as the “severe” bleomycin model. To ensure the study only included mice which developed fibrosis the following criteria were applied to all mice treated with bleomycin. Exclusion criteria A (no development of fibrosis): for inclusion in the study mice had to have a weight loss of 3% or greater by day 7 from day 0 and noticeable fibrotic regions on the micro-CT scans. Exclusion criteria B (morbid weight loss): if any mice lost 30% or more of their body weight from day 0, they were euthanized and removed from analysis. Exclusion criteria C: mice that died from unexpected complications of experimental procedures or treatments. Exclusion criteria A was in place prior to the beginning of the study. Exclusion criteria B was instituted based on Gilhodes et al., 2017. Mouse numbers used in experiments as a result of these exclusion criteria are listed in Supplementary Data 6.

### B6_BP_dslf binder treatments

C57BL/6 mice were intraperitoneally injected with B6_BP_dslf binder every other day starting at day 7 from bleomycin instillation and ending on day 19 for a total of 7 treatments administered. Mice were injected with B6_BP_dslf with a dose of 100 µg/kg or 1 mg/kg body weight with a volume of 10 µl/g body weight per injection. For the inhalation study, mice were anesthetized using 5% isoflurane and hung semi-recumbently by their top incisors and their tongue retracted to visualize the vocal cords. B6_BP_dslf was delivered every other day with an oropharyngeal addition of 25–50 µL with drug concentrations of 43.6 and 185.2 µg/kg. The B6_BP_dslf was pipetted into the oral cavity and was audibly inhaled into the lungs.

### flexiVent lung mechanics assessment

On day 21 from bleomycin instillation, lung mechanics measurements were performed as described in Gilhodes et al and Devos et al.^71,72^. These lung mechanics measurements were performed using the flexiVent FX system (SCIREQ Inc., Montreal Qx, Canada). The instrument was equipped with a FX1 module and a Negative Pressure-Driven Forced Expiration (NPFE) extension for mice run by flexiWare 8.0 software. C57BL/6 mice were anesthetized using an intraperitoneal injection of ketamine-xylazine (100 and 10 mg/kg body weight) in 0.9% sterile saline. Once mice were observed to be in a surgical plane of anesthesia, the trachea was exposed to insert a 22-gauge metal cannula. The mice were then attached to the flexiVent and received an intraperitoneal injection of 0.8 mg/kg body weight pancuronium bromide to prevent spontaneous breathing. Mice were ventilated with a tidal volume of 10 mL/kg with a frequency of 150 breaths/min and an end-expiratory pressure of 3 cmH_2_O. The baseline was recorded, and the following scripts were run three times: Deep Inflation, Snapshot-150, Quick Prime, Negative Pressure-Driven Forced Expiration (NPFE). After the scripts were completed, mice were euthanized.

### High-resolution micro-computed tomography (HR-μCT) scans

Mice were induced with 1.5–2.5% isoflurane and placed into the Quantum GX Micro-CT scanner (Perkin Elmer, Waltham, MA) attached to a nose cone to continue the delivery of anesthetic. Micro-CT images of the lungs were acquired using the following parameters: 90 kV, 88 uA, acquisition FOV 36 mm, Al 0.5 mm + Cu 0.06 mm filter, acquisition time 4 min under High-Resolution conditions. Scans were calibrated for Hounsfield units (HU) and analyzed using AccuCT^TM^ Advanced Imaging (Perkin Elmer, Waltham, MA). A semi-automatic segmentation process was used to isolate the left and right lobes of the lung. By performing HU histogram analysis of frequency of intensities for a sample of the scan image, the threshold for lung tissue was determined. Using these thresholds, the lungs were segmented. The frequency of intensities was determined for the segmented lung. Captured lung volume, dense tissue volume, and mean intensity were also calculated. Dense tissue was defined as anything above the intensity where non-treated mice and bleomycin-treated mice intersected on the frequency of intensities histogram.

### Masson trichrome staining

The left lobe of the lung was inflated with 4% paraformaldehyde at 25 cm H_2_O of pressure. Lungs were stored in PFA overnight and then washed with PBS and dehydrated using a series of ethanol washes and then processed and paraffinized overnight using the Shandon Citadel 1000 tissue processor. After the tissues were paraffinized, they were embedded into paraffin blocks and sectioned into 7 µm slices using a Microm 325 microtome. Tissue was then deparaffinized as described previously (IHC Deparaffinization Protocol, Abcam). Masson trichrome staining was performed according to manufacturer’s instructions (Sigma Aldrich, HT15-1KT).

### Sirius red staining and quantification

After the lungs were inflated, paraffinized, and sectioned as described above each 7 µm thick paraffin sections were first deparaffinized then rehydrated. Slides were immersed in 1% phosphomolybdic acid solution^73^. The slides were rinsed in water and submerged in a 0.1% Sirius red solution for 60 min at room temperature. Slides were immersed in 2 baths of acidified water and then dehydrated^74^. These tissue sections were imaged by an Olympus BX51 microscope at 4X magnification. Those images were individually analyzed through ImageJ software following the established NIH protocol^75^.

### Ashcroft scoring of masson trichrome-stained lung slices

After Masson trichrome staining was performed, slides were scored using a modified Ashcroft scoring applied to the whole lung divided into 10x fields where sections were given a score of 0-8 per section and the total score is defined by scores summed and divided by the number of sections^76^.

### Bronchoalveolar lavage fluid (BALF) collection and cell counts

BALF was collected by intubating the trachea with a 22-gauge cannula. 800 µL of PBS was instilled and recovered from the lungs via syringe following three repetitive plunges. BALF was centrifuged at 10,000 rpm for 10 min, after which the supernatant was collected, and the pellet resuspended in PBS. A 250 µL resuspension of the cell pellet was spun down onto slides using the Cytospin™ 3 Centrifuge for 3 min at 800 rpm. Slides were stained using Wright’s Stain and imaged at 1000× on the Olympus BX51 Microscope^77^. A total of 200 cells were differentially counted for macrophages, lymphocytes, and polymorphonucleocytes in duplicate and cell counts were averaged^78^.

### BALF cytokine array

The supernatant of the BALF was collected as stated above and stored at −80 °C prior to processing. When thawed on ice, HALT™ Protease and Phosphatase Inhibitor Cocktail 100X (48446, ThermoFisher Scientific) was added to a final concentration of 1X and then total protein concentration was determined using the Pierce™ BCA Protein Assay Kit (ThermoScientific). Each treatment group (NT, BLM, and B6_BP_dslf 185.2 µg/kg) had three supernatants pooled together at equal concentrations. The cytokine expression was measured through the Mouse Inflammation Antibody Array - Membrane (40 Targets) (ab133999, abcam) according to manufacturer’s instructions. Membranes were imaged using FluorChem imaging system (Alpha Innotech, San Jose, CA) The membranes were quantified using ImageJ software as described in Schindelin et al.^79^. The resulting densitometry for the individual cytokines were then analyzed using one-way ANOVA.

### Immunofluorescence staining

Paraffin-embedded lung sections were deparaffinized using Histo-Clear (HS-200, National Diagnostics) three times for 5 min each time, after which the slides were rehydrated in serial ethanol concentrations in water for 5 min each step (that is, 100%-100%-80%-80%-70%-70%-30%-30%-0%). Antigen retrieval was done as described previously using citraconic anhydride solution^80^. The slides were washed three times with PBS-T then blocked using 50% Mouse-to-Mouse blocking reagent (MTM500, ScyTek Laboratories) in Seablock blocking buffer (37527, ThermoFisher Scientific) for an hour. Anti-α Smooth Muscle Actin (1:200, Cat No. 48938, Cell Signaling Technology) and anti-Collagen type I (1:250, Cat No. PA5-95137, Invitrogen) primary antibodies were diluted in 10% Seablock PBST and incubated with the samples overnight at 4 °C. The slides were then washed three times with 10% Seablock PBST. The samples were then incubated with corresponding secondary antibodies Goat anti-Rabbit IgG (Heavy chain), Superclonal™ Recombinant Secondary Antibody, Alexa Fluor™ 647 (1:500, Cat No. A27040, Thermo Fisher Scientific) and Goat anti-Mouse IgG (H + L), Superclonal™ Recombinant Secondary Antibody, Alexa Fluor™ 488 (1:500, Cat No. A28175, Thermo Fisher Scientific) and a DAPI counterstain (14285, Cayman). The slides were then mounted using diamond antifade (P36961, Thermo Fisher Scientific) and cure at room temperature for at least 24 h prior to imaging. Confocal imaging was done using a Leica TCS SP8 Hyvolution confocal microscope with LASX software (Leica, version 3.1.1.15751). Argon laser power was consistently set to 25%. Use of the HyD detectors were preferred over the PMT detectors and gains for the detectors are set to less than 100. The images were taken at 1024×1024 pixel format. The final images were exported as TIFF files and gamma was not adjusted for any images.

### Sandwich ELISA to quantify B6_BP_dslf in lung homogenate and serum samples

To extract B6_BP_dslf from lung tissue, pre-weighed entire lung was homogenized in 1 mL tissue protein extraction reagent (T-PER, ThermoFisher, #78510) + 1X Halt protease/phosphatase inhibitor cocktail (ThermoFisher, #78440) using Omni ceramic bead tubes (Omni cat # 19–627) and an Omni electric homogenizer for 3 rounds of 20 sec at full speed (8.0 m/s). Homogenized samples were then incubated on ice for 2 h, centrifuged at 19,000 × *g* for 20 min at 4 °C, and isolated supernatants were stored at −80 °C until use in the sandwich ELISA.

Recombinant human integrin αvβ6 or αvβ8 headpieces at 1.0 μg/mL in TBS with Ca^2+^/Mg^2+^ (20 mM Tris pH 8.0, 150 mM NaCl, 1 mM Ca^2+^Cl_2_, 1 mM Mg^2+^Cl_2_) were incubated overnight at 4 °C on 96-well Nunc MaxiSorp plates (ThermoFisher, #442404) (100 μL/well). Plates were blocked with 200 μL/well of blocking buffer (2% [w/v] bovine serum albumin [BSA] in TBS with 0.05% Tween20 [TBST]) and incubated for 1 h at room temperature. Plates were washed 3× in TBST using a robotic plate washer (BioTek). 100 μL of lung homogenate supernatant or serum dilutions for each mouse starting at 1:100 and serially diluting 5-fold seven times using TBS with Ca^2+^/Mg^2+^ and 0.5% (w/v) BSA (binding buffer) (8 total dilutions) were added to each well and incubated for 1 h at room temperature. Each 96-well plate with biological sample dilutions also included wells with 100 μL of B6_BP_dslf standard starting at 1 nM and serially diluting 2-fold ten times using binding buffer (11 total dilutions). After washing plates 3× in TBST, plates were incubated for 30 min at room temperature with 100 μL/well of 10 nM rabbit anti-B6_BP_dslf mAb called “A1” diluted in binding buffer. After washing plates 3× in TBST, plates were incubated for 30 min at room temperature with 100 μL/well of goat anti-rabbit IgG HRP-linked mAb (Cell Signaling Technology, #7074) diluted 10,000-fold in binding buffer. Following a final 3× TBST plate wash, 100 μL of TMB (3,3′,5′,5-tetramethylbenzidine, SeraCare, #5120-0083) was added to each well and rested for 2 min. TMB was quenched with 100 μL of 1 N HCl. Absorbance at 450 nm was immediately collected for each well on an Agilent BioTek Epoch 2 plate reader. Data were plotted in Prism 9 (GraphPad Software, San Diego, CA) to determine the 50% effective concentration (EC_50_) using a four-parameter logistic regression model. A logarithmic equation fit to the linear portion of the sigmoidal curve of the B6_BP_dslf standard curve, lung weights, and EC_50_ values for lung homogenate supernatant and serum were used to calculate B6_BP_dslf concentration (nM) in mouse lung tissue and undiluted sera.

### Quantification of collagen, fibronectin, and p-SMAD2 in lung homogenates

Whole lung tissue was homogenized using 300 uL RIPA buffer (10 mM Tris-Cl (pH 8.0), 1 mM EDTA, 1% Triton X-100, 0.1% sodium deoxycholate, 0.1% SDS, 140 mM NaCl, and 1 mM PMSF) and HALT™ Protease and Phosphatase Inhibitor Cocktail 100X (48446, ThermoFisher Scientific) was added to a final concentration of 1X, and ran at 60 Hz for a total of 30 s per manufacturer instructions(). The tissue lysate was collected, the total protein concentration determined using Pierce ™ BCA Protein Assay Kit. Protein samples were separated through 5–10% SDS-PAGE gels and transferred to 0.2 µm nitrocellulose membranes (Bio-Rad, Cat No.1620150, Hercules, CA) through electro blotting. To block, 1% BSA in PBS and Mouse-to-Mouse Blocking Reagent (MTM500, ScyTek Laboratories Inc.) were used and the following primary antibody probes were used overnight: Collagen I Polyclonal Antibody (1:500, Cat. No. PA5-95137), Fibronectin Polyclonal Antibody (Cat. No. PA5-29578, ThermoFischer Scientific), and Phospho-SMAD2 (Ser465/Ser467) (E8F3R) Rabbit mAb (1:500, Cat. No. 18338, Cell Signaling Technology). After washing the primary antibodies off, secondary antibodies IRDye® 800CW Donkey Anti-Rabbit IgG (H + L) (Cat No. 926–3221, Licor, Lincoln, NE, 1: 15,000) and Goat anti-Mouse IgG (H + L) Highly Cross-Adsorbed Secondary Antibody, Alexa Fluor 680 (Cat. No. A-21058, Invitrogen, 1: 5,000) were used. The blots were developed using the Odyssey CLx (Model No. 9140, Li-Cor, Lincoln, NE). Quantifications were done by using ImageJ. Three biological replicates were used for each treatment group.

### Collagen quantification using Sircol ™ soluble and insoluble collagen assay

Whole lung tissue was homogenized as described above then 100 µL of homogenate was incubated with 1 mL of acetic acid-pepsin digest overnight and the rest of the Biocolor Sircol™ Soluble Collagen Assay (S1000, Ilex Life Science) and Biocolor Sircol™ Insoluble Collagen Assay (S2000, Ilex Life Science) were done following manufacturers instructions. Three biological replicates were used for each treatment group.

### Human fluorescent lung organoid triculture (hFLO) bleomycin model

A 3D human alveolar triculture model was created as previously described^40^. Alveolar type II cell line A549 (ATCC CCL-185), endothelial cell EAHy (ATCC RL-2922), and human normal lung fibroblast cell HFL1 (ATCC CCL-153) were incorporated in a suspension culture supplemented with 300 µg mL^−1^ basement membrane extract (BME). These aggregates were grown until day 7 of culture and then treated with 20 μg mL^−1^ bleomycin (13877, Cayman Chemical Company). Media was changed after 3 days of culture with bleomycin. As a treatment, we used 10 nM B6_BP_dslf. The cultures were kept in treatment media for 4 more days with media changing every other day. Cell aggregates were collected and fixed in 4% paraformaldehyde for 15 min, washed with PBS-T three times, 15 min each time and resuspended in 15% and 30% sucrose in PBS prior to embedding in OCT compound (Sakura FineTek). 10 µm sections were then stained, imaged, and analyzed as described previously^40^.

### Statistical analysis

All values were reported as mean ± SD. Data were analyzed by one-way ANOVA followed by Tukey’s post-hoc test for multiple comparisons. Outliers were identified using Grubb’s test (Q = 5%). Analysis and graphs were done with GraphPad Prism 9.0 (GraphPad, San Diego, CA. USA). Results with *p*-value < 0.05 were considered statistically significant. Analysis was done blinded to the treatment group assigned.

### Software

In silico design the analysis of the inhibitors was performed using a combination of bash, python, and Rosetta. All scripts used in this paper have been uploaded to a github account (https://github.com/aroy10/avb6-publication). Data analyses were performed with custom code in Python and IPython.

### Reporting summary

Further information on research design is available in the Nature Portfolio Reporting Summary linked to this article.

### Supplementary information

Supplementary Information

Peer Review File

Description of Additional Supplementary Files

Supplementary Data 1

Supplementary Data 2

Supplementary Data 3

Supplementary Data 4

Supplementary Data 5

Supplementary Data 6

Reporting Summary

### Source data

Source Data

## Data Availability

Structures from the first-round design and B6B8_BP_dslf monomer have been deposited in the Protein Data Bank with accession numbers 7LMV [10.2210/pdb7LMV/pdb] and 7LMX [10.2210/pdb7LMX/pdb], respectively. Structures of αvβ8 + B8_BP_dslf and αvβ6 + B6_BP_dslf have been deposited in the Electron Microscopy Data Bank (EMDB) with the accession numbers EMD-41153 and EMD-41154, and Protein Data Bank with the acession numbers PDB ID 8TCF [10.2210/pdb8TCF/pdb] and PDB ID 8TCG [10.2210/pdb8TCG/pdb]. 5FFO [10.2210/pdb5FFO/pdb] and 4UM9 [10.2210/pdb4UM9/pdb] coordinates were downloaded from Protein Data Bank. Source data are provided within this paper. Source data are provided with this paper.

## Code availability

Detailed step by step protocol and all associated codes have been deposited on github (https://github.com/aroy10/avb6-publication) and are available from the main authors upon request. XMLs for designing the disulfide bond discussed in the main text have also been deposited in the same repository.
