# Supplementary material for: De novo design of highly selective miniprotein inhibitors of integrins αvβ6 and αvβ8
Source: Nat Commun. 2023 Sep 13;14:5660. doi: 10.1038/s41467-023-41272-z (PMC10500007; doi:10.1038/s41467-023-41272-z)
Supplement: Supplementary file 1 — Supplementary Information [file 41467_2023_41272_MOESM1_ESM.pdf]

Supplementary Figures, Tables, and Legends

Supplementary Fig. 1: Crystal structures of designed αvβ6 inhibitors from first and second round design strategies.
Supplementary Fig. 2: Metal-dependent binding of designed proteins to human αvβ6.
Supplementary Fig. 3: Flow cytometry screening of second-round designed protein binders using human αvβ6.
Supplementary Fig. 4: Sorting scheme for the site-saturation mutagenesis (SSM) library of av6_3.
Supplementary Fig. 5: Experimental optimization of av6_3 for binding and heatmap of key evolved residues.
Supplementary Fig. 6: BLI binding of purified mutants against titrations of biotinylated human αvβ6/αvβ8.
Supplementary Fig. 7: Selectivity of designed αvβ8 inhibitors against αvβ6 and αvβ8 using yeast surface display.
Supplementary Fig. 8: Selectivity of designed αvβ6 inhibitors against other RGD-binding integrins.
Supplementary Fig. 9: Thermal stability of B6_BP_dslf under reducing and non-reducing conditions.
Supplementary Fig. 10: TMLC and CAGA-reporter co-culture assays assessing αvβ6-mediated inhibition of TGF-β1.
Supplementary Fig. 11: *In vivo* imaging of αvβ6 (+) A431 tumors using fluorescently-labeled B6_BP.
Supplementary Fig. 12: Lung and serum pharmacokinetics of B6_BP_dslf in healthy male C57BL/6 mice.
Supplementary Fig. 13: HR-μCT imaging, histopathology, and lung function after IP B6_BP_dslf in "mild" bleo-model.
Supplementary Fig. 14: CT frequency, lung function, and cellular responses after IP B6_BP_dslf in "mild" bleo-model.
Supplementary Fig. 15: HR-μCT imaging and lung function after IP B6_BP_dslf in "severe" bleo-model.
Supplementary Fig. 16: Lung function and cellular responses after OA B6_BP_dslf in "severe" bleo-model.
Supplementary Fig. 17: Stability of B6_BP_dslf following nebulization.
Supplementary Fig. 18: Negative stain EM 2D class averages, SDS-PAGE, SEC, and cryoEM statistics.
Supplementary Fig. 19: Processing schematic for cryoEM of αvβ6 + B6_BP_dslf and αvβ8 + B8_BP_dslf complexes.
Supplementary Table 1: Kinetic analysis of BLI binding of purified mutants against titrations of biotinylated human αvβ6.
Supplementary Table 2: Affinity and selectivity comparison of B6_BP against other leading reported αvβ6 inhibitors.
Supplementary Table 3: Predicted and observed interactions between integrins αvβ6/αvβ8 and designed minibinders.
Supplementary Table 4: Statistics of X-ray diffraction and structure refinement.
Supplementary Table 5: Exclusion criteria for animals in bleomycin-induced lung fibrosis model.

Supplementary Figures

a.

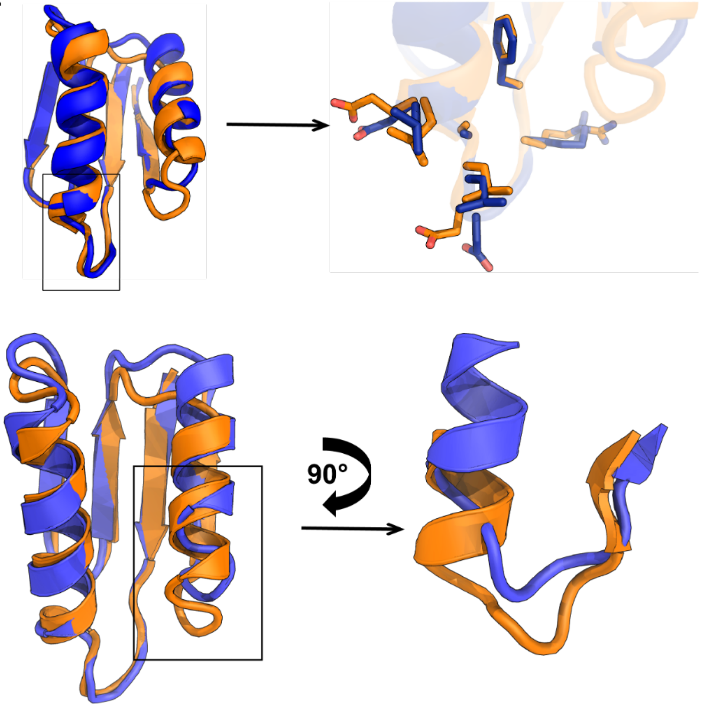

b.

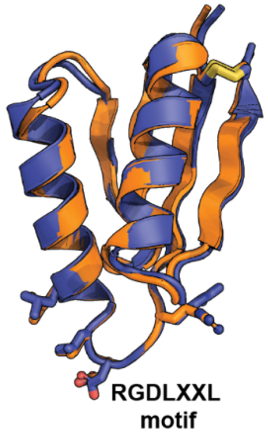

Supplementary Fig. 1: Crystal structures of designed αvβ6 inhibitors from first and second round design strategies. **a)** Crystal structure (blue) of the evolved variant from the first round of design superimposed onto the design model (orange). Although the first part of the crystal structure including the RGD loop (Top panel) overlaid well with the design model, there was rigid body movement of the C-terminal helix of the fold equivalent to one helical turn (bottom panel). For the second generation of designs, the crystal structure of the previous round was superimposed onto αvβ6 by aligning the RGD motif. Two loops were sampled for length and conformation and 16 designs were ordered in the second round. **b)** Superposition of the designed B6B8_BP_dslf (orange) and the crystal structure (blue) in the cartoon model. The designed disulfide bond and the RGDLXXL motif is shown in stick representation.

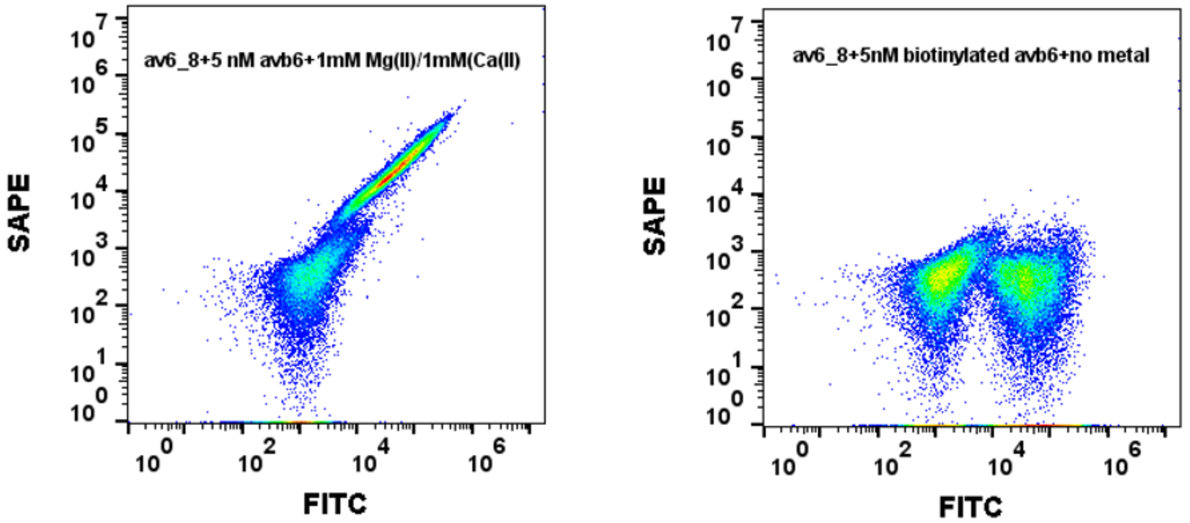

Supplementary Fig. 2: Metal-dependent binding of designed proteins to human αvβ6. The designed protein shows metal dependent binding to αvβ6. In the absence of any metal, there is no detectable binding (left panel) as compared to in presence of 1mM Ca(II)/1mM Mg(II) (right panel).

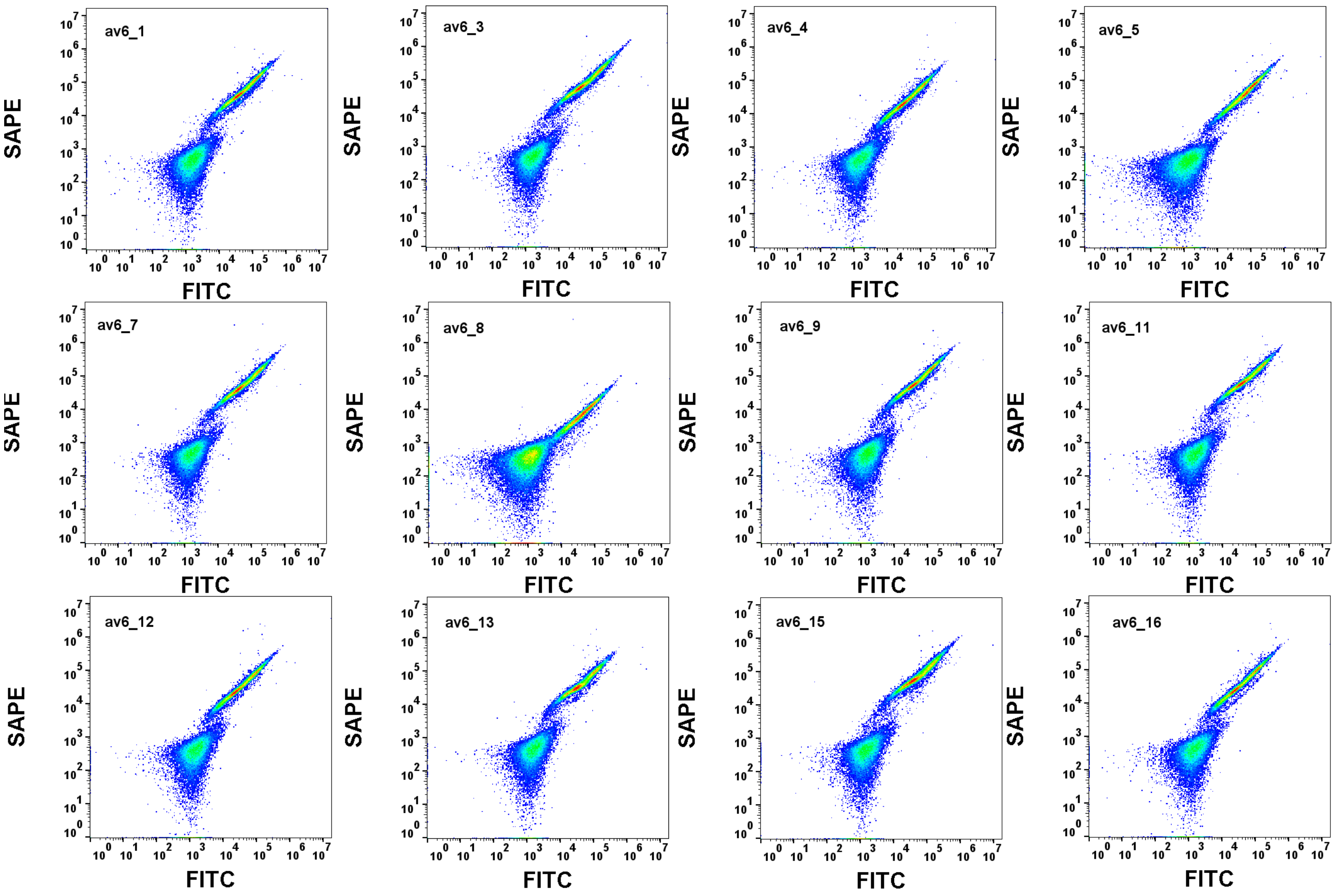

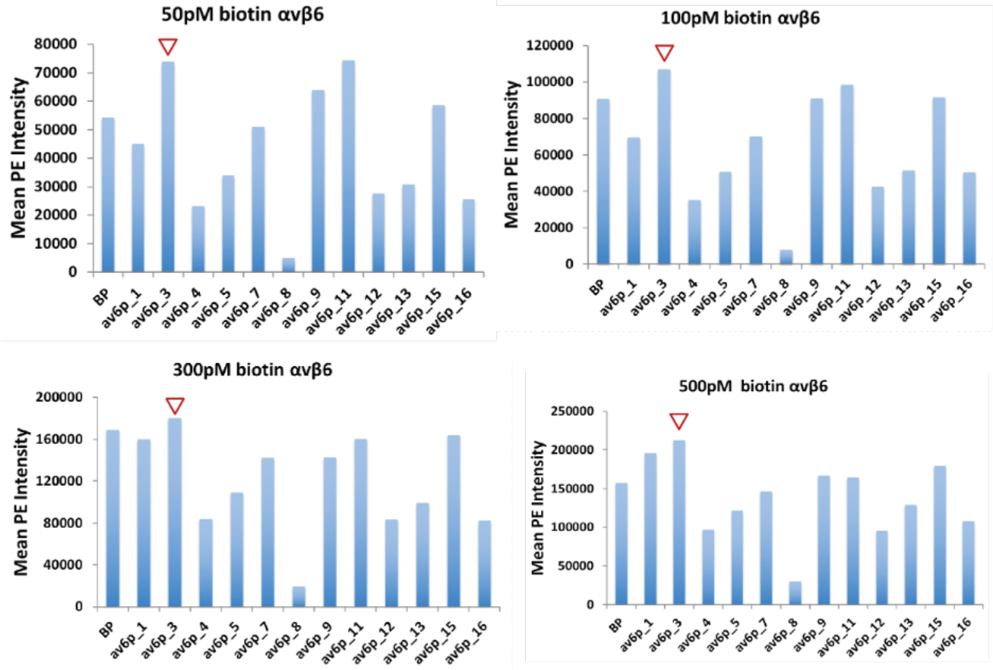

Supplementary Fig. 3: Flow cytometry screening of second-round designed protein binders using human αvβ6. (Top Panel) Designs were expressed using yeast surface display technique and incubated with 50 pM of biotinylated human αvβ6. (Bottom Panel) Mean PE intensity of BP (binding protein) and all second round designs at 50 pM, 100 pM, 300 pM, and 500 pM of biotinylated integrin αvβ6. av6_3 shows the highest binding signal (PE fluorescence signal) across all concentrations tested.

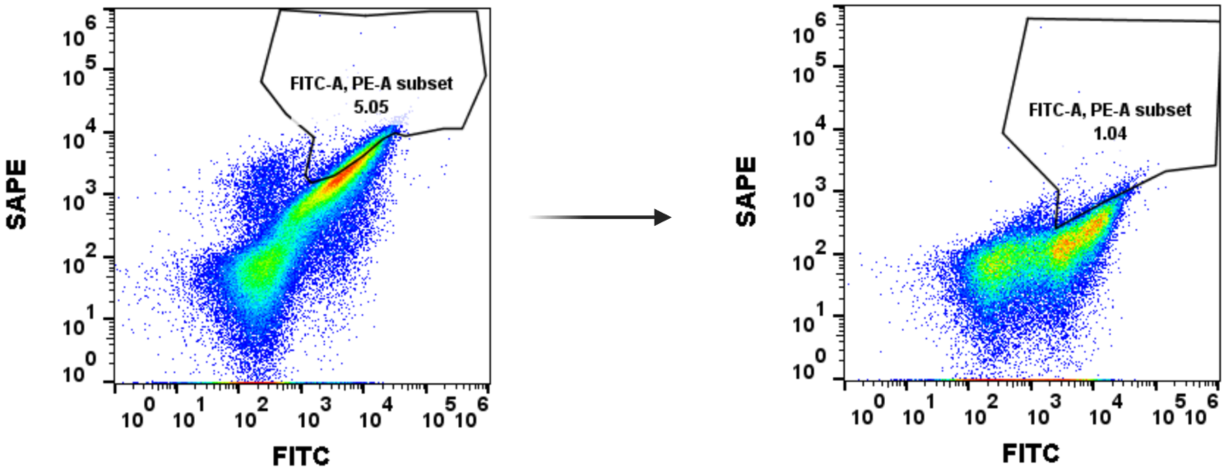

Supplementary Fig. 4: Sorting scheme for the site-saturation mutagenesis (SSM) library of av6_3. For the first round of selection, the library was incubated with 200 pM of biotinylated human αvβ6 and the top 5% of the binders were collected. For the second and final round of selection, 100 pM of biotinylated αvβ6 was used and the top ~1% of the binding population was collected.

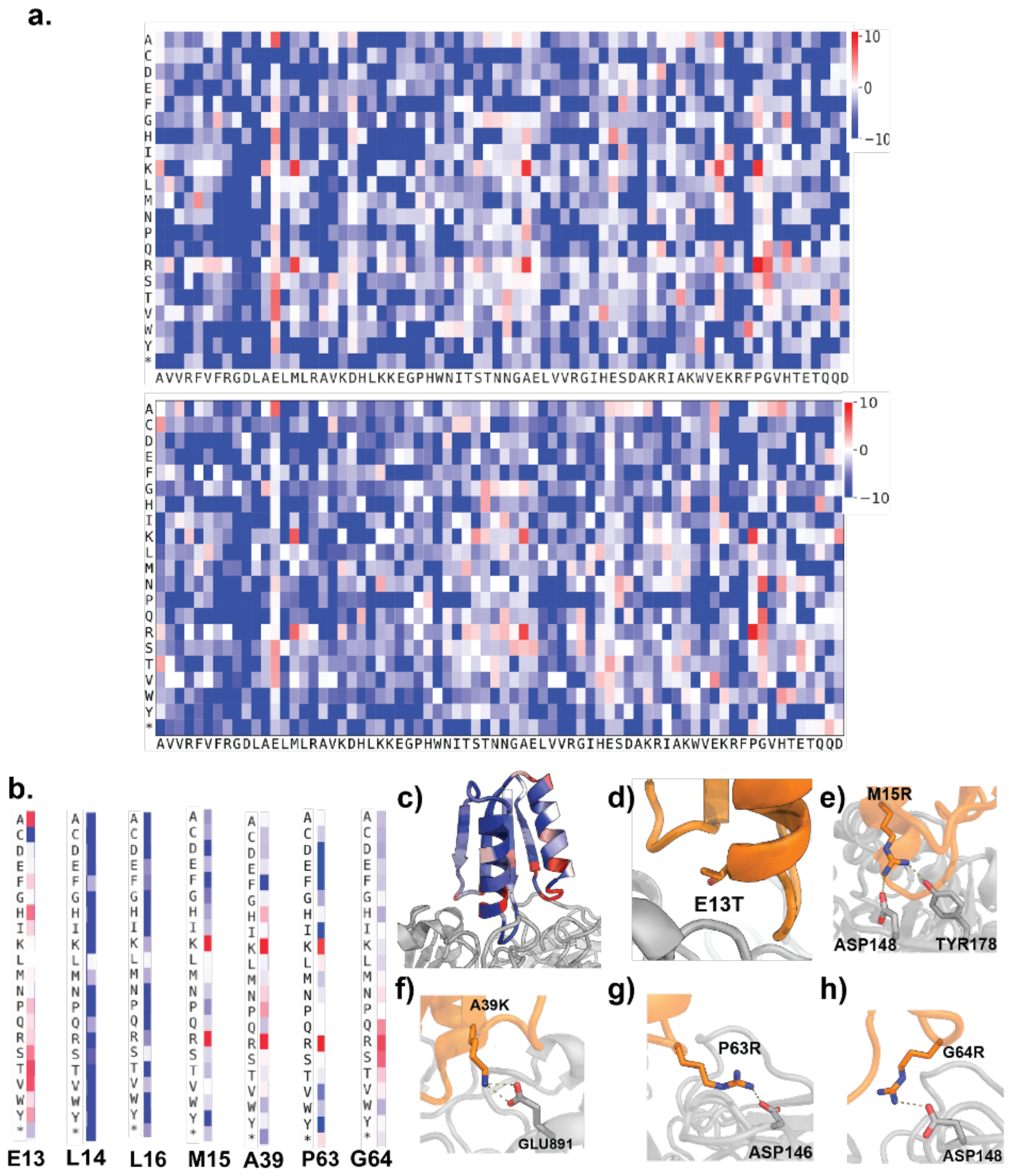

Supplementary Fig. 5. Experimental optimization of av6_3 for binding and heatmap of key evolved residues. a) Enrichment ratio of the evolved variants compared to the naive library after two rounds of sorting with increasing stringency (200 pM and 100 pM) from biological replicates. Beneficial mutations are colored in red and deleterious mutations are colored in blue. b) Heat map of key enriched and conserved residues derived from SSM optimization of the inhibitor. E13 prefers to be small polar residues or small hydrophobic residues. L14 and L16 of the RGD<u>L</u>XX<u>L</u> motif are highly conserved, demonstrating the importance of the LXXL motif. M15/A39/P63/G64 mutations are mainly charge-complementary to the receptor. c) The majority of the enriched substitutions increase charge complementarity to the receptors. d) E13 prefers to be small polar or hydrophobic. e) M15R/K is well within hydrogen bonding interaction range to D148 and Y178 of αvβ6. f) A39K substitution facing the β6 subunit likely introduces a salt bridge with Glu891. g,h) Two consecutive residues (P63 and G64) on Loop2 facing the αv subunit are enriched as positively charged Lys or Arg residue, which likely form salt bridges with two acidic residues D146 and D148 on the αv subunit of the receptor, respectively.

a.

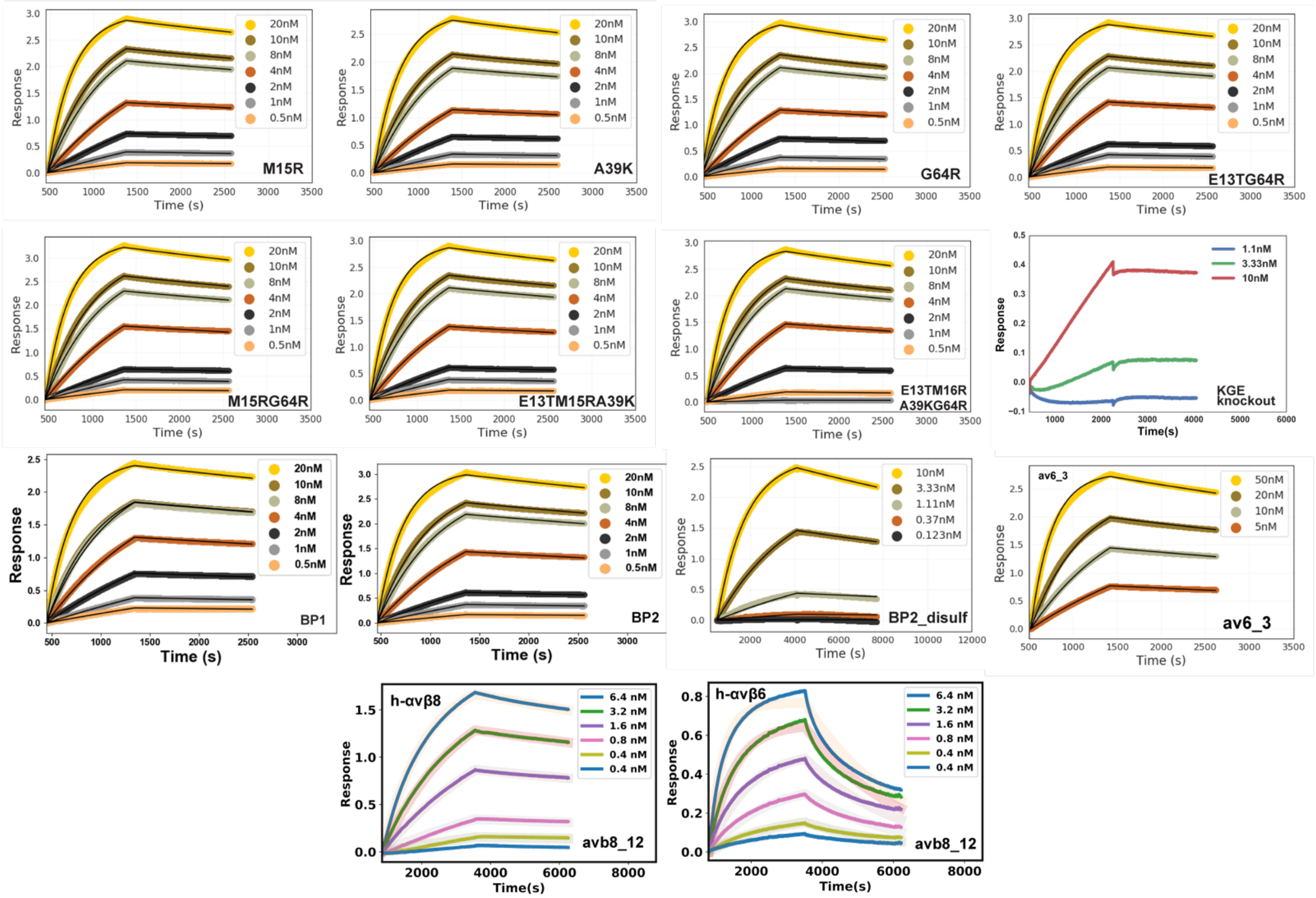
M15R
A39K
G64R
E13TG64R
M15RG64R
E13TM15RA39K
E13TM16R A39KG64R
KGE knockout
BP1
BP2
BP2_disulf
av6_3
h-αvβ8
h-αvβ6
avb8_12
20nM
10nM
8nM
4nM
2nM
1nM
0.5nM
1.1nM
3.33nM
3.33nM
1.11nM
0.37nM
0.123nM
50nM
5nM
6.4 nM
3.2 nM
1.6 nM
0.8 nM
0.4 nM
Response
Time (s)
Time(s)

b.

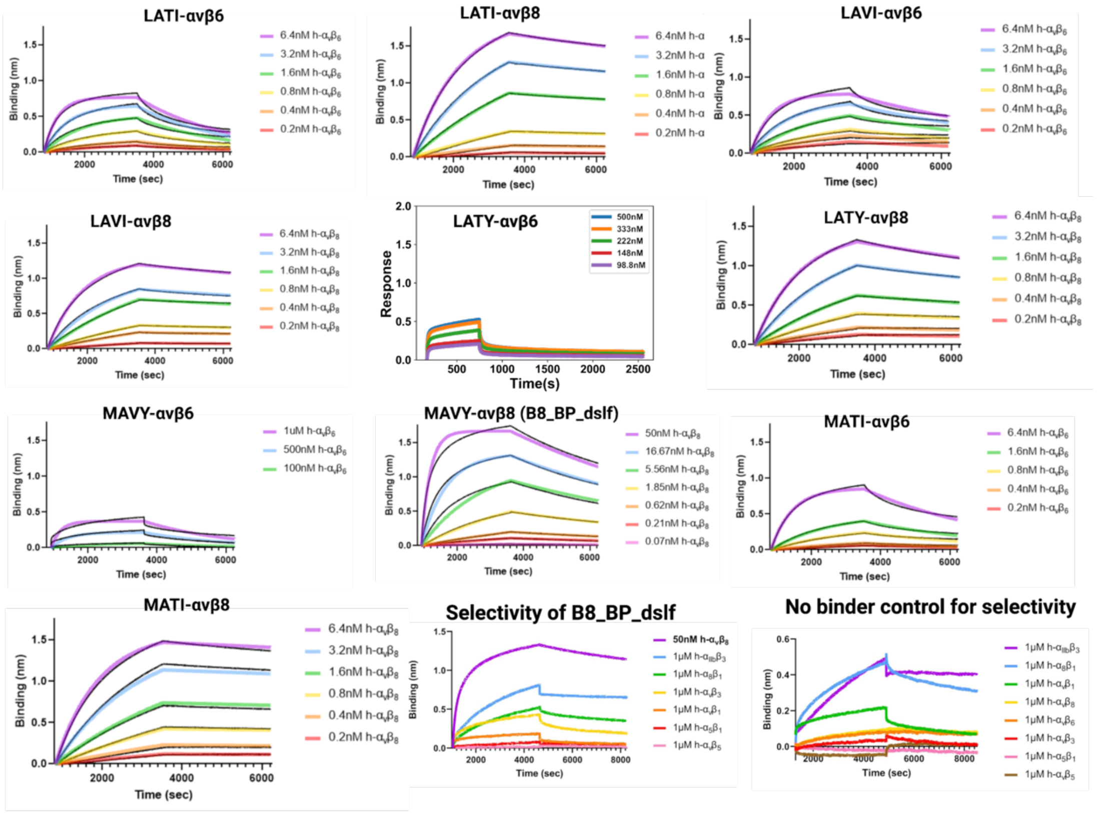
LATI-αvβ6
LATI-αvβ8
LAVI-αvβ6
LAVI-αvβ8
LATY-αvβ6
LATY-αvβ8
MAVY-αvβ6
MAVY-αvβ8 (B8_BP_dslf)
MATI-αvβ6
MATI-αvβ8
Selectivity of B8_BP_dslf
No binder control for selectivity
Binding (nm)
Response
Time (sec)
Time(s)
500nM
333nM
222nM
148nM
98.8nM

Supplementary Fig. 6: Bio-layer interferometry (BLI) binding of all purified mutants against titrations of human αvβ6, αvβ8, and other RGD integrins. (a) See Table S1 for kinetic analysis values. All panels are binding against αvβ6 titrations, except the bottom left panel that shows binding of avb8_12 to αvβ8. **(b)** Binding for the B8_BP_dslf point mutants and selectivity for B8_BP_dslf for RGD integrins.

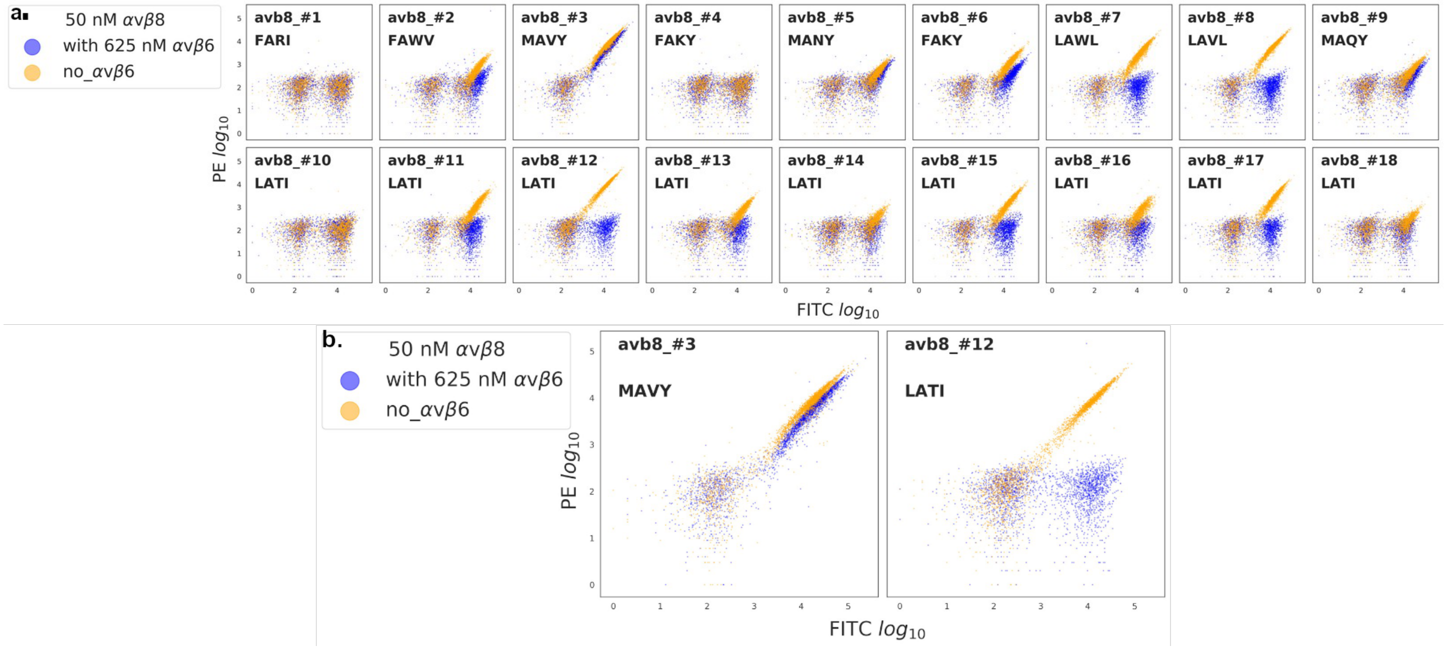

Supplementary Fig. 7: Competition assay using yeast surface display technique to determine selectivity of designed αvβ8 binders. a) Designs were expressed using yeast surface display technique and incubated with 50 nM of FLAG-tagged αvβ8 and 625nM of unlabelled αvβ6. Binding was detected by anti-FLAG-PE conjugated antibody from abcam (Product # ab72469). **b)** Effect of -RGDLXXL motif on selectivity of the designed binders. Mutation of -RGDMAVY motif (avb8_#3 or B8_BP_dslf) to -RGDLATI (avb8_#12) completely abrogates selectivity towards αvβ8.

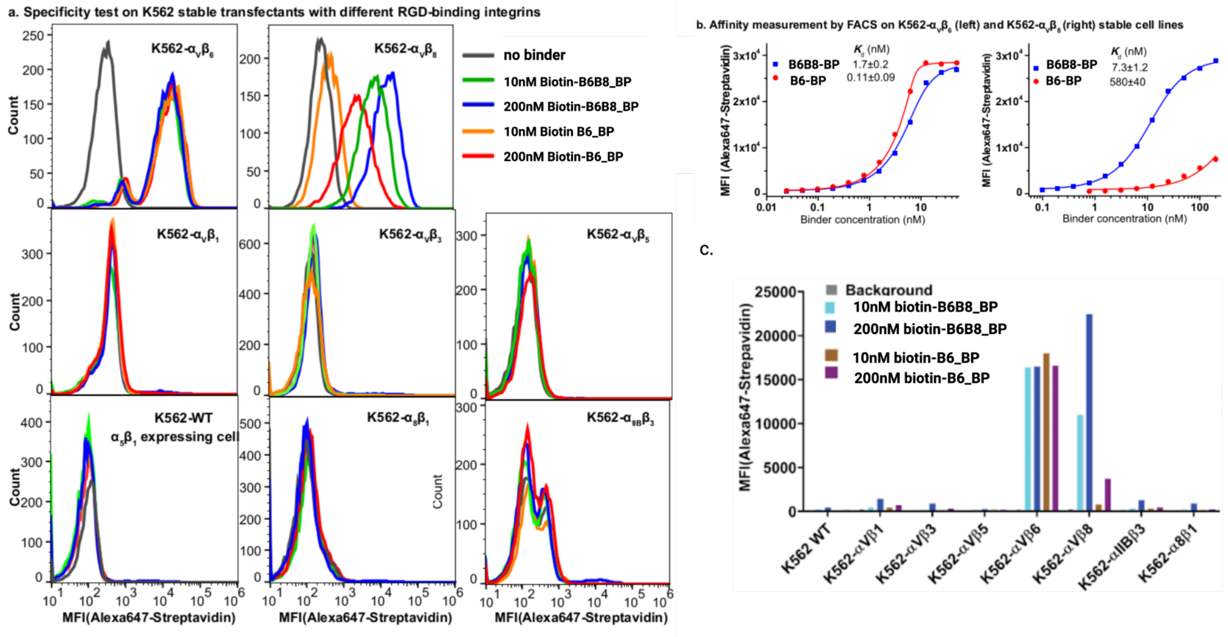

Supplementary Fig. 8: Selectivity of designed αvβ6 inhibitors against other RGD-binding integrins. a) Selectivity of B6B8_BP and B6_BP against other RGD-binding integrins: B6B8_BP/B6_BP does not bind to αvβ1, αvβ3, αvβ5, α5β1, α8β1, and αiibβ3 integrins up to 200 nM concentration. Assays were performed using biotinylated B6B8_BP/B6_BP via an N-term Avitag and K562 cells stably transfected with different integrins. All the incubation and washes were done in the assay buffer, L15 medium with 1% BSA. In detail, cells were incubated with biotinylated binders with indicated concentrations at 22℃ for 1h, followed by three washes and staining with Alexa-647 streptavidin for 20 minutes, followed by another three washes and subject to flow cytometry. **b)** Dose-dependent titration of B6B8_BP and B6_BP on K562 cells stably transfected with αvβ6 (left) and αvβ8 (right) with the same method described in panel a. B6B8_BP and B6_BP binds to αvβ6 with K_d values of 1.7 (± 0.2) and 0.11 (± .09) nM, respectively, and binds to αvβ8 with K_d values of 7.3 (± 1.2) and 580 (±40) nM, respectively. Values are reported as mean ± SD **c)** Selectivity of B6B8_BP and B6_BP against 8 -RGD binding integrins: K562 cells stably transfected with different integrins were incubated with biotinylated B6B8_BP and B6_BP followed by staining with Alexa-647 streptavidin. B6_BP binding is highly specific to αvβ6, whereas B6B8_BP binds to both αvβ6 and αvβ8. Both B6B8_BP and B6_BP show negligible binding up to 200 nM of concentration against other 6 integrins.

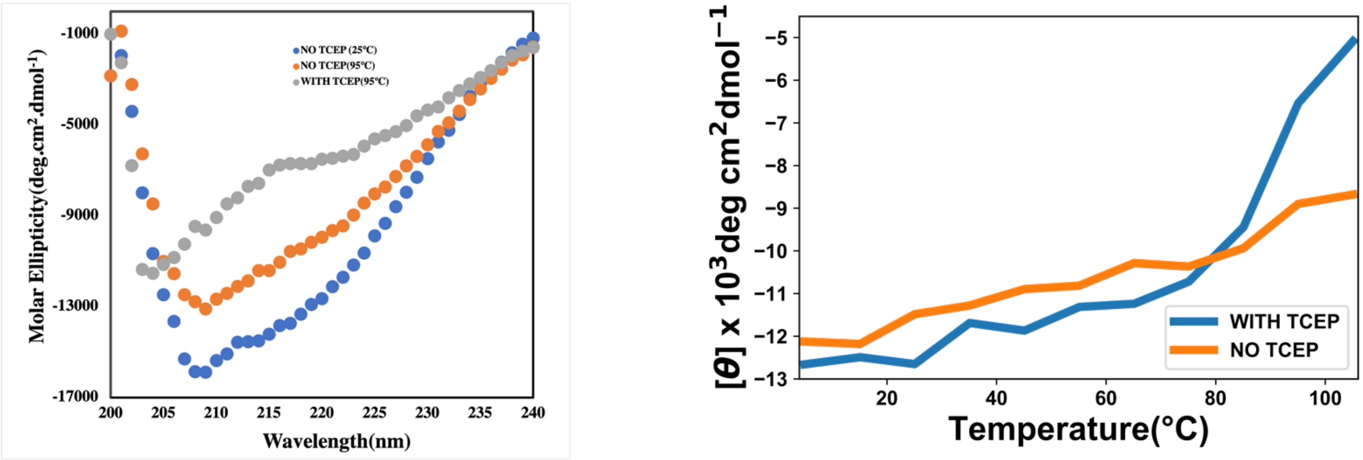

Supplementary Fig. 9: Thermal stability of B6_BP_dslf under reducing and nonreducing conditions. a) Circular dichroism (CD) wavelength scan of B6_BP_dslf at 25°C and 95°C in the presence of 1 mM reducing agent (TCEP). b) Thermal melt of B6_BP_dslf monitored by CD spectra in the presence/absence of a reducing agent. B6_BP_dslf maintains its signature CD spectra consistent with a mixed alpha/beta fold under non-reducing conditions. Under reducing conditions, B6_BP_dslf melts ~90°C indicating hyper-thermostability is partially mediated by the engineered disulfide bond.

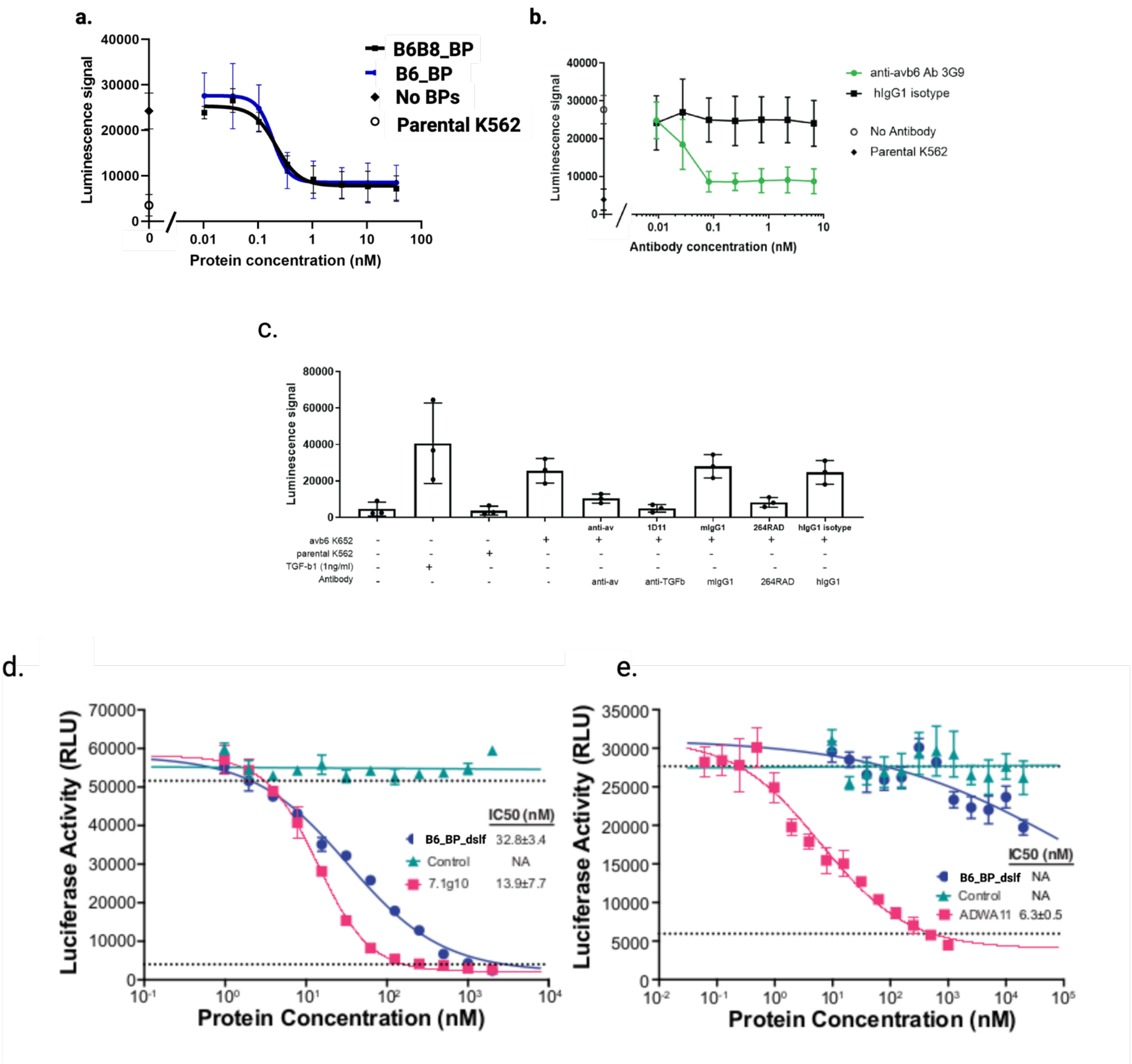

Supplementary Fig. 10: TMLC and CAGA-reporter co-culture assays assessing αvβ6-mediated inhibition of TGF-β1. a) TGF-β inhibition mediated by B6B8_BP and B6_BP in TMLC:αvβ6 K562 co-culture assay. Both B6B8_BP and B6_BP blocks αvβ6 mediated TGF-β activation with similar IC_{50} values (216 pM and 188 pM, respectively). Mean ± SD are plotted. b) Positive and negative controls for αvβ6-mediated TGF-β activation in TMLC:αvβ6 K562 co-culture assay. An αvβ6-specific antibody 3G9 (known clinically as STX-100 and BG00011) can inhibit TGF-β activation in the TMLC assay, whereas the human IgG1 isotype control has no effect on αvβ6 mediated TGF-β activation. Mean ± SD are plotted. c) Additional controls for TMLC assay: Recombinant TGF-β induces a clear increase in luciferase activity, showing that the TMLCs can respond to active TGF-β. Anti TGF-β, anti-αv, and an anti-αvβ6/β8 (264-RAD) are capable of blocking TGF-β activation in the TMLC assay. See methods section for detailed description for each antibody used in this assay. Mean ± SD are plotted. d,e) B6_BP_dslf selectively inhibits αvβ6-mediated TGF-β1 activation. αvβ6 (d) and αvβ8 (e) transfectants were co-incubated with CAGA-reporter cells and GARP/TGF-β1 transfectants and inhibitors. Control: irrelevant nanobody. 7.1g10: inhibitory antibody to integrin β6. ADWA11, an inhibitory antibody to integrin β8. Mean ± SD are plotted, and IC_{50} values for each inhibitor are reported. Lower dashed lines represent integrin-independent TGF-β1 activation, which is 7.8% of the total activation with αvβ6 transfectants (upper dashed line in d) and 13.3% of the total activation with αvβ8 transfectants (upper dashed line in e). Each experiment was performed in triplicates (n=3).

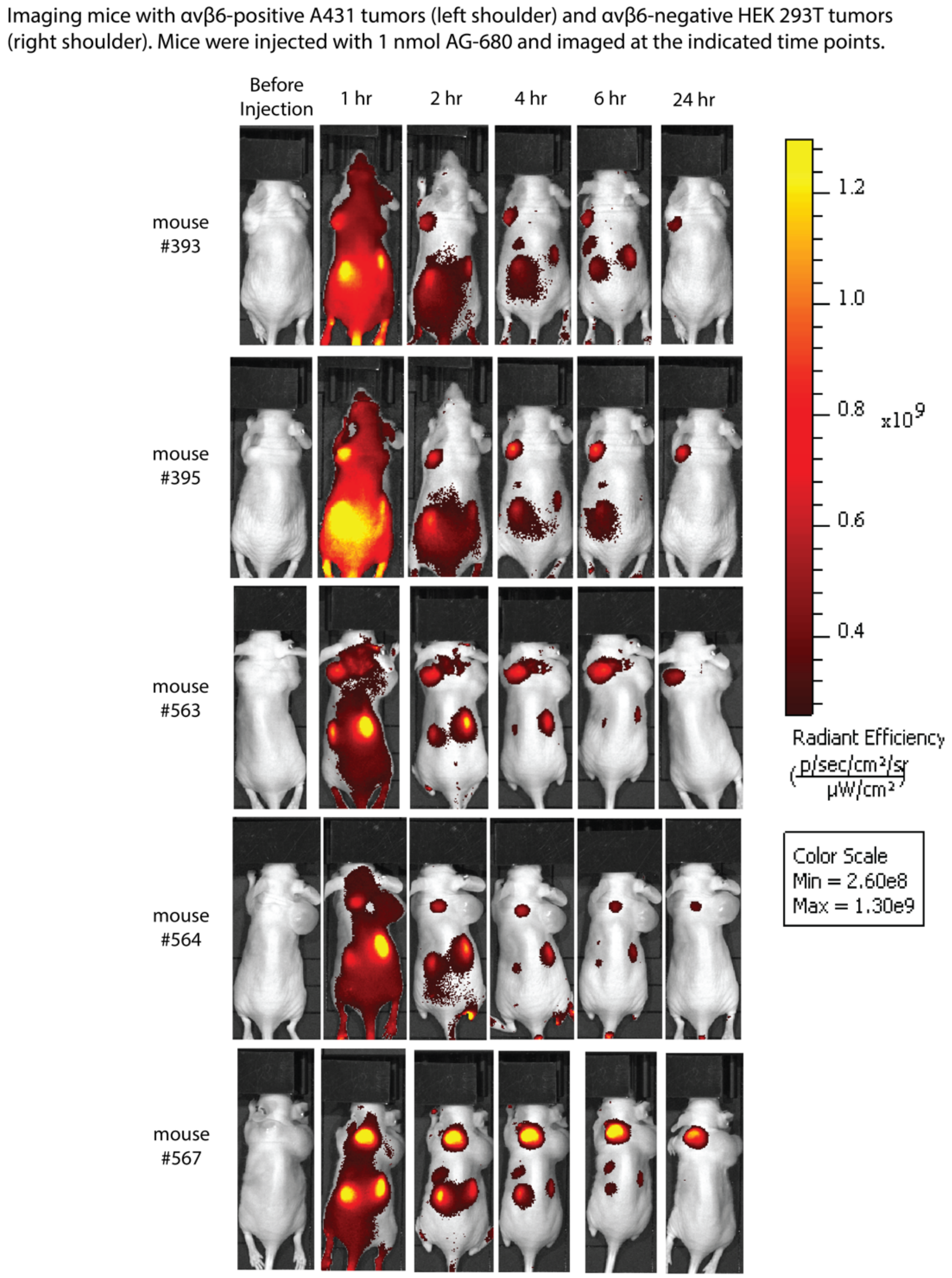

Supplementary Fig. 11: *In vivo* imaging of αvβ6 (+) A431 tumors using fluorescently-labelled B6_BP. Imaging αvβ6 (+) tumors *in vivo*; athymic nude mice (n=5) were injected with αvβ6 (+) A431 cells on the left shoulder and αvβ6 (-) HEK293T cells on the right shoulder. AlexaFluor-680-labelled B6_BP (AF680-B6_BP) was injected via tail vein to image the tumors over time as indicated.

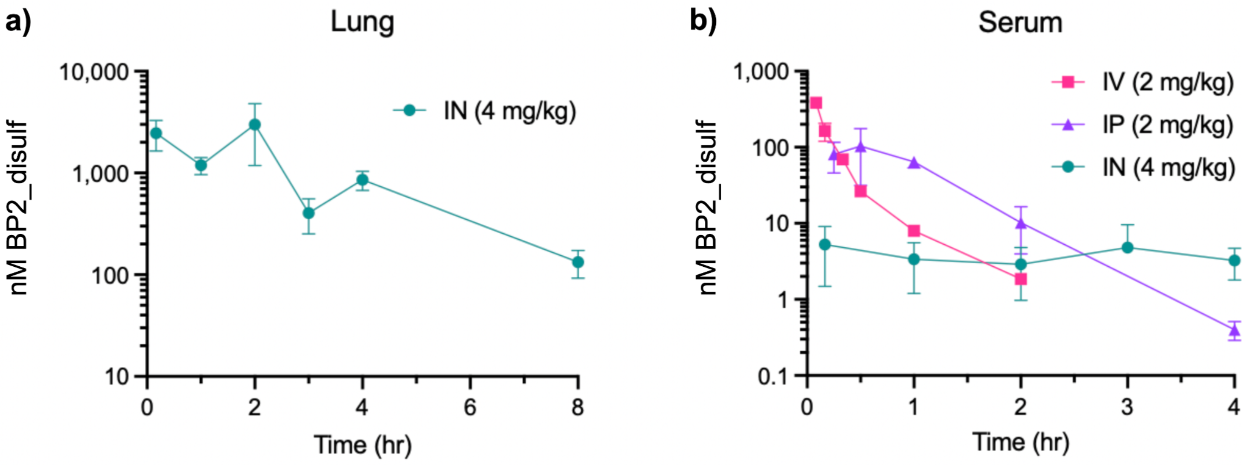

c)

Biological sample	BP2_disulf dose (mg/kg)	Route of administration	Terminal elimination half-life ($t_{1/2,z}$)
Lung	4	Intranasal (IN)	55.2 ± 15.6 min
Serum	2	Intravenous (IV)	10.5 ± 0.8 min
Serum	2	Intraperitoneal (IP)	11.2 ± 1.8 min

Supplementary Fig. 12: Lung and serum pharmacokinetics of B6_BP_dslf in healthy male C57BL/6 mice following a single intravenous or intraperitoneal 2 mg/kg dose, or intranasal 4 mg/kg dose. To characterize the pharmacokinetics of B6_BP_dslf following different routes of administration, 8-12 week old male C57BL/6 mice were given 2 mg/kg B6_BP_dslf via the intravenous (IV) or intraperitoneal (IP) routes, or 4 mg/kg B6_BP_dslf via the intranasal (IN) route. B6_BP_dslf concentrations were quantified in (a) lung tissue and (b) serum samples collected at different time points post-administration using a sandwich enzyme linked immunosorbent assay (ELISA) method. (a,b) Following intranasal administration, serum concentrations were on average 484-fold lower than the lung concentrations over 4 h. (b) The area under the curve from 0 to 2 hours ($AUC_{0\text{-}2hr}$) following IP administration was ~2-fold higher than the $AUC_{0\text{-}2hr}$ from the IV route (7,569.7 ± 1,055.6 nM•min vs. 3,892.9 ± 925.4 nM•min). (c) Terminal elimination half-lifes ($t_{1/2,z}$) of B6_BP_dslf in lung and serum following different routes of administration. The elimination of B6_BP_dslf from the lungs within hours and low relative serum concentrations compared to lung concentrations following IN administration is consistent with the lung and serum pharmacokinetics observed for intranasal GSK3008348 in healthy C57BL/6 mice[1]. Each experiment was performed using n=5 mice/group. All values are reported as mean ± SD.

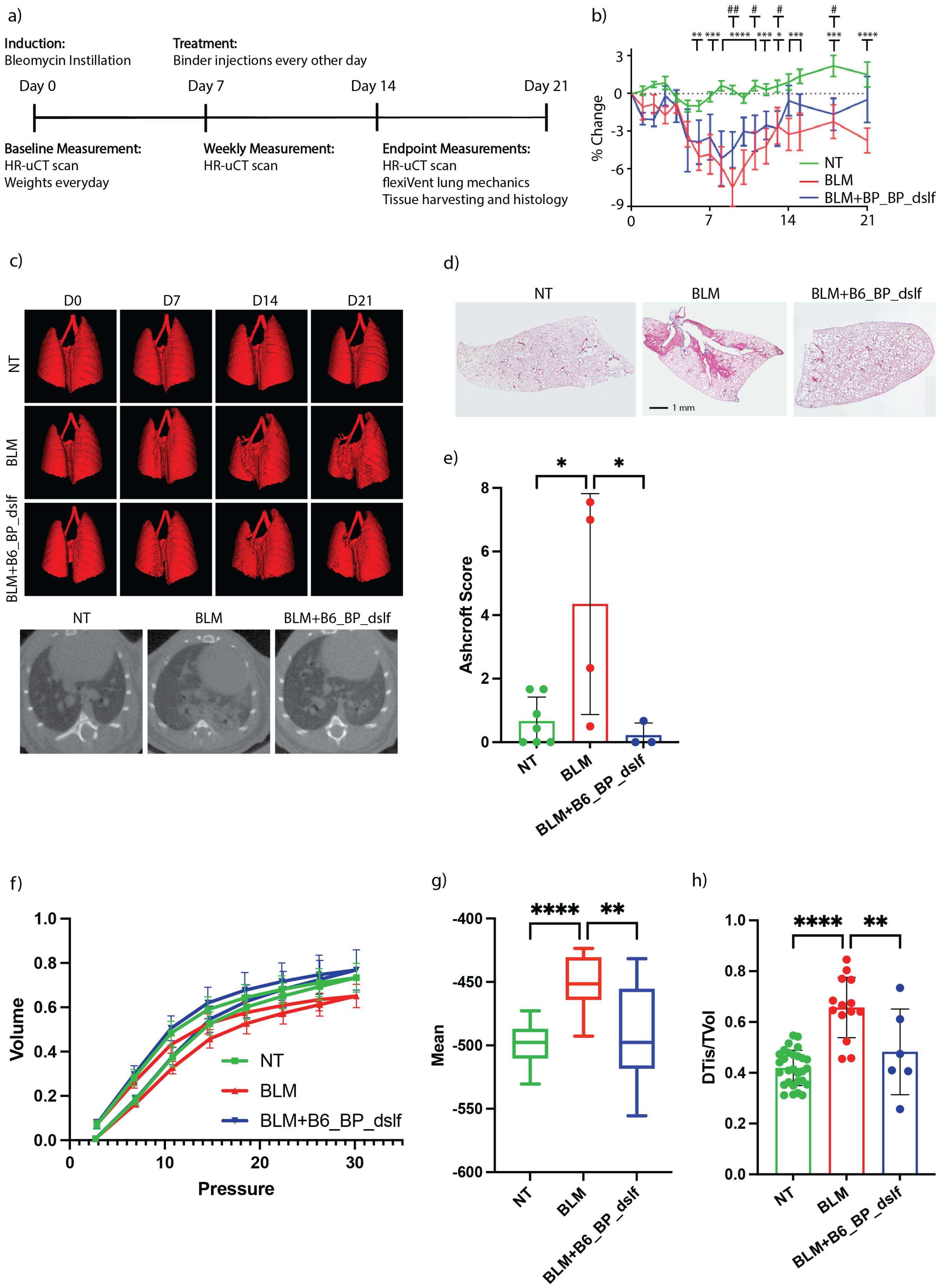
a)
Induction:
Bleomycin Instillation
Treatment:
Binder injections every other day
Day 0
Day 7
Day 14
Day 21
Baseline Measurement:
HR-uCT scan
Weights everyday
Weekly Measurement:
HR-uCT scan
Endpoint Measurements:
HR-uCT scan
flexiVent lung mechanics
Tissue harvesting and histology
b)
% Change
NT
BLM
BLM+BP_BP_dslf
c)
D0
D7
D14
D21
NT
BLM
BLM+B6_BP_dslf
NT
BLM
BLM+B6_BP_dslf
d)
NT
BLM
BLM+B6_BP_dslf
1 mm
e)
Ashcroft Score
NT
BLM
BLM+B6_BP_dslf
f)
Volume
Pressure
NT
BLM
BLM+B6_BP_dslf
g)
Mean
NT
BLM
BLM+B6_BP_dslf
h)
DTis/TVol
NT
BLM
BLM+B6_BP_dslf

Supplementary Fig. 13: High-resolution micro-computed tomography (HR-µCT) imaging, histopathology, and lung function following B6_BP_dslf intraperitoneal (IP) administration in the "mild" bleomycin-induced pulmonary fibrosis mouse model. a) Treatment regimen for bleomycin-induced pulmonary fibrosis. Mice were intratracheally administered bleomycin at 1 U/kg body weight. Mice were injected intraperitoneally with B6_BP_dslf binder at 100 µg/kg every-other-day starting at day 7 post-bleomycin instillation, and ending on day 19, for a total of 7 treatments administered (NT). b) Weight changes for NT, BLM and BLM+B6_BP_dslf groups (data represented as mean ± SD, Tukey's t-test, NT n=24; BLM n=14; BLM+B6_BP_dslf n=5) asterisks above error bars notates statistical significance between NT and BLM and # above error bars notates statistical significance between NT vs BLM+B6_BP_dslf using a Two-Way ANOVA followed by Tukey's t-test where p-values were adjusted for multiple comparisons. c) Upper and lower panel: High-resolution micro-CT scans with asterisks at fibrotic areas in the lower lobes lung. d) Masson-Trichrome staining for NT, BLM and BLM+B6_BP_dslf treated mice. e) Gradation of fibrotic burden: Average Ashcroft Scoring (mean ± SEM, Tukey's t-test, NT vs BLM P value=0.0259, BLM vs BLM+B6_BP_dslf P value=0.0398) of histological slides for NT (0.66), BLM (4.347) and BLM+B6_BP_dslf (0.22) treated mice. f) Representative pressure-volume loop curves (PV-loops) for NT, BLM and B6_BP_dslf groups (mean ± SEM, NT n=17; BLM n=7; BLM+B6_BP_dslf n=5). g) Mean intensities of micro-CT scan (box and whiskers show the mean and the maximum and minimum values, Tukey's t-test, NT vs BLM P value=<0.0001, NT vs BLM+B6_BP_dslf P value=0.7677, BLM vs BLM+B6_BP_dslf P value=0.0019, NT n=29; BLM n=14; BLM+B6_BP_dslf n=6). h) Quantification of lung density from micro-CT scans (mean ± SD, Tukey's t-test, NT vs BLM P value=<0.0001, BLM vs BLM+B6_BP_dslf P value=0.0023, NT=29; BLM n=14, BLM+B6_BP_dslf n=6). For all graphs, * p-value< 0.05, ** p-value<0.01, *** p-value<0.001, **** p-value<0.0001 from two-tailed Tukey's t-test following ANOVA and were adjusted for multiple comparisons.

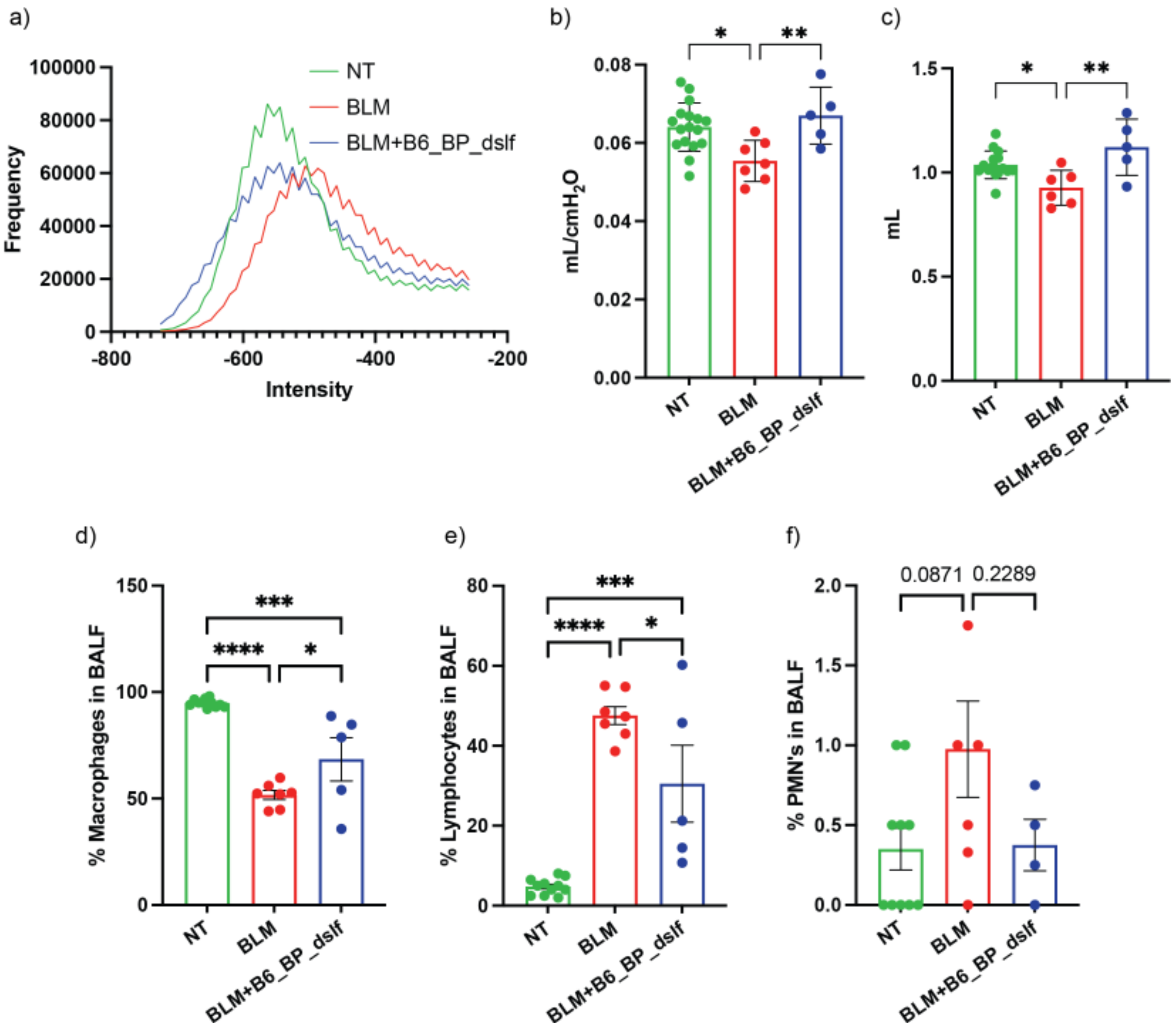

Supplementary Fig. 14: Computed tomography (CT) imaging frequency of intensities, lung function, and cellular responses following B6_BP_dslf intraperitoneal (IP) administration in the "less severe" bleomycin-induced pulmonary fibrosis mouse model. a) Frequency of intensities of CT scans in Hounsfield Units (data represented as mean intensity value, NT n=29; BLM n=14; BLM+B6_BP_dslf n=6). b, c) Measurement of lung mechanics on Day 21 for NT, BLM and BLM+B6_BP_dslf. Both static compliance (NT vs BLM P value=0.0116, BLM vs BLM+B6_BP_dslf P value=0.0096, NT n=17; BLM n=7; BLM+B6_BP_dslf n=5) and forced vital capacity (NT vs BLM P value=0.0371, BLM vs BLM+B6_BP_dslf P value=0.003, NT n=15; BLM n=6; BLM+B6_BP_dslf n=5) improves and nears NT groups following B6_BP_dslf treatment as compared to the BLM only group. d,e,f) Differential counts of cellular response 21 days post bleomycin administration: d) macrophages (NT vs BLM P value=<0.0001, NT vs BLM+B6_BP_dslf P value=0.0005, BLM vs BLM+B6_BP_dslf P value=0.0373, NT n=11; BLM n=7; BLM+B6_BP_dslf n=5) e) lymphocytes, (NT vs BLM P value=<0.0001, NT vs BLM+B6_BP_dslf P value=0.0004, BLM vs BLM+B6_BP_dslf P value=0.0263, NT n=11; BLM n=7; BLM+B6_BP_dslf n=5) and f) polymorphonucleocytes. (NT vs BLM P value=0.0871, BLM vs BLM+B6_BP_dslf P value=0.2289, NT n=10; BLM n=7; BLM+B6_BP_dslf n=4) All data are represented as mean ± SEM with * p-value< 0.05, ** p-value<0.01, *** p-value<0.001, **** p-value<0.0001 from two-tailed Tukey's t-test following ANOVA and were adjusted for multiple comparisons.

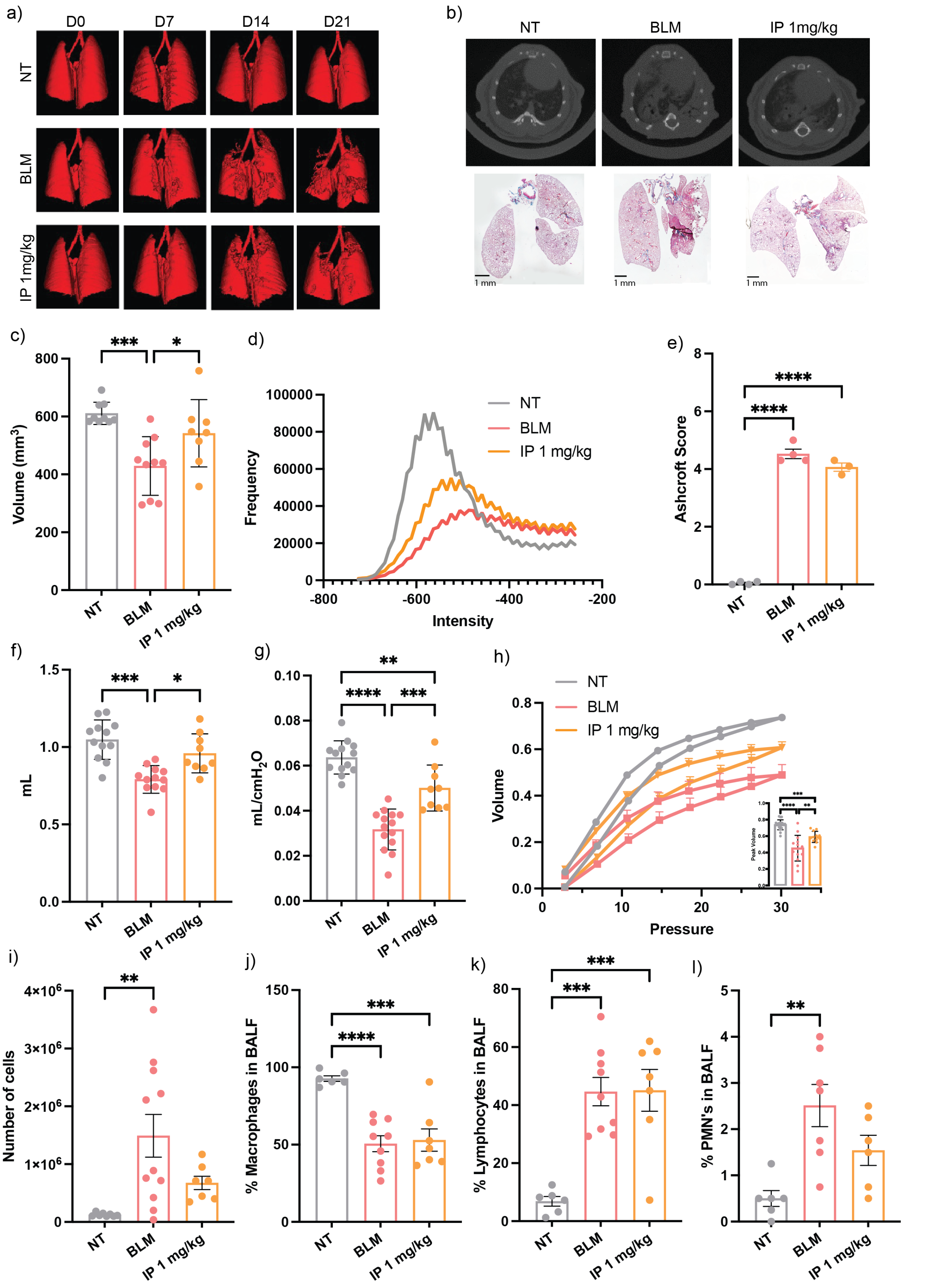
a)
D0
D7
D14
D21
NT
BLM
IP 1mg/kg
b)
NT
BLM
IP 1mg/kg
1 mm
1 mm
1 mm
c)
Volume (mm³)
***
*
NT
BLM
IP 1 mg/kg
d)
Frequency
Intensity
NT
BLM
IP 1 mg/kg
e)
Ashcroft Score
****
****
NT
BLM
IP 1 mg/kg
f)
mL
***
*
NT
BLM
IP 1 mg/kg
g)
mL/cmH2O
**
****
***
NT
BLM
IP 1 mg/kg
h)
NT
BLM
IP 1 mg/kg
Volume
Pressure
Peak Volume
i)
Number of cells
**
NT
BLM
IP 1 mg/kg
j)
% Macrophages in BALF
***
****
NT
BLM
IP 1 mg/kg
k)
% Lymphocytes in BALF
***
***
NT
BLM
IP 1 mg/kg
l)
% PMN's in BALF
**
NT
BLM
IP 1 mg/kg

Supplementary Fig. 15: High-resolution micro-computed tomography (HR-μCT) imaging and lung function following B6_BP_dslf intraperitoneal (IP) administration in the "more severe" bleomycin-induced pulmonary fibrosis mouse model. a) Longitudinal three-dimensional renderings of HR-μCT scans show the development of fibrosis. b) Upper panel: Representative HR-μCT scans at Day 21 with lower panel: representative whole lung stained with Masson Trichrome for NT, BLM, and B6_BP_dslf 1 mg/kg groups harvested at Day 21. c) Quantification of lung volumes from HR-μCT scans on Day 21 (mean ± SD, Tukey's t-test, NT vs BLM P value=0.0006, BLM vs B6_BP_dslf IP 1 mg/kg P value=0.0382, NT n=9, BLM n=10, B6_BP_dslf IP 1 mg/kg n=8). d) Histograms of the frequency of intensities from HR-μCT scans on Day 21. e) Average Ashcroft Score from Masson Trichrome sections (mean ± SD, Tukey's t-test, NT vs BLM P value=<0.0001, NT vs B6_BP_dslf IP 1 mg/kg P value=<0.0001, BLM vs B6_BP_dslf IP 1 mg/kg P value=0.0243, NT n=5, BLM n=3, B6_BP_dslf IP 1 mg/kg n=3). f) Forced Vital Capacity (mean ± SEM, Tukey's t-test, NT vs BLM P value=<0.0001 , NT vs B6_BP_dslf IP 1 mg/kg P value=0.0032 , BLM vs B6_BP_dslf IP 1 mg/kg P value=<0.0001 , NT n=12, BLM n=12, B6_BP_dslf IP 1 mg/kg n=9). g) Static Compliance (mean ± SEM, Tukey's t-test, NT vs BLM P value=<0.0001 , NT vs B6_BP_dslf IP 1 mg/kg P value=0.0032 , BLM vs B6_BP_dslf IP 1 mg/kg P value=<0.0001 , NT n=13, BLM n=14, B6_BP_dslf IP 1 mg/kg n=9). h) Pressure-Volume Loops measured by the SCIREQ flexiVent FX at Day 21 with peak volumes in the inset graph show a rescue from the restrictive nature of BLM-induced fibrosis. (mean ± SEM, Tukey's t-test, NT vs BLM P value=<0.0001 , NT vs B6_BP_dslf IP 1 mg/kg P value=0.0008 , BLM vs B6_BP_dslf IP 1 mg/kg P value=0.0034 , NT n=20, BLM n=14, B6_BP_dslf IP 1 mg/kg n=12). i) Total BALF cell count at Day 21 (mean ± SD, Tukey's t-test, NT vs BLM P value=0.0044 , NT n=8, BLM n=11, B6_BP_dslf IP 1 mg/kg n=7). Differential cell counts j) Macrophages (mean ± SD, Tukey's t-test, NT vs BLM P value=<0.0001 , NT vs B6_BP_dslf IP 1 mg/kg P value=0.0003 , NT n=6, BLM n=9, B6_BP_dslf IP 1 mg/kg n=7). k) Lymphocytes (mean ± SD, Tukey's t-test, NT vs BLM P value=0.0003, NT vs B6_BP_dslf IP 1 mg/kg P value=0.0004 , NT n=6, BLM n=9 B6_BP_dslf IP 1 mg/kg n=7). and l) PMNs (mean ± SD, Tukey's t-test, NT vs BLM P value=0.0026, NT n=6, BLM n=7, B6_BP_dslf IP 1 mg/kg n=6). All data were analyzed using a two-tailed Tukey's t-test following ANOVA: * p-value< 0.05, ** p-value<0.01, *** p-value<0.001, **** p-value<0.0001, and were adjusted for multiple comparisons.

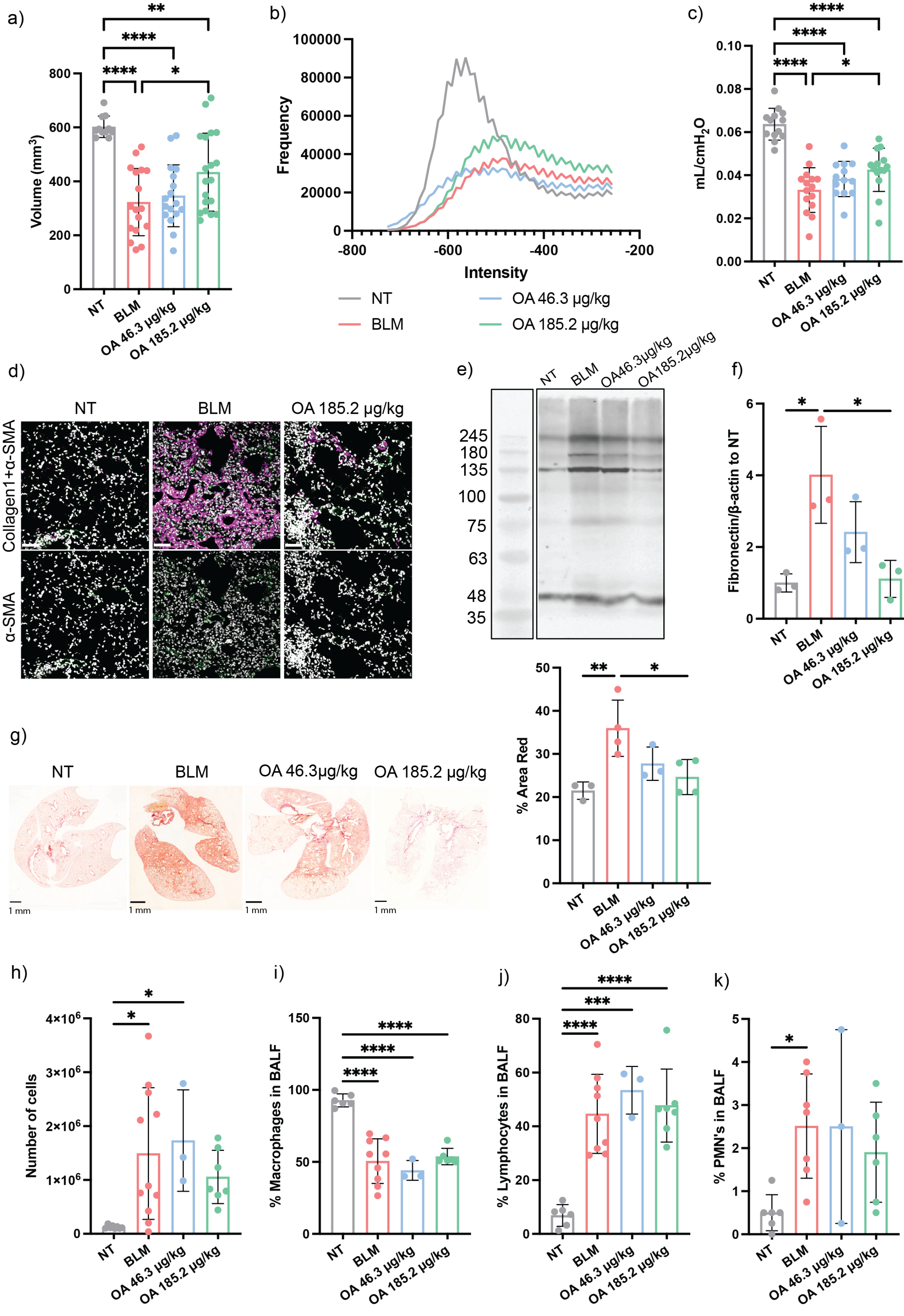
a)
Volume (mm³)
NT
BLM
OA 46.3 µg/kg
OA 185.2 µg/kg
b)
Frequency
Intensity
NT
BLM
OA 46.3 µg/kg
OA 185.2 µg/kg
c)
mL/cmH2O
d)
NT
BLM
OA 185.2 µg/kg
Collagen1+α-SMA
α-SMA
e)
NT
BLM
OA46.3µg/kg
OA185.2µg/kg
245
180
135
100
75
63
48
35
f)
Fibronectin/β-actin to NT
g)
NT
BLM
OA 46.3µg/kg
OA 185.2 µg/kg
1 mm
% Area Red
h)
Number of cells
i)
% Macrophages in BALF
j)
% Lymphocytes in BALF
k)
% PMN's in BALF

Supplementary Fig. 16: Lung mechanics and cellular responses following B6_BP_dslf oropharyngeal administration (OA) in the "more severe" bleomycin-induced pulmonary fibrosis mouse model. a) Quantification of HR-uCT volumes on Day 21 (data represented as mean ± SD, Tukey's t-test, NT vs BLM P value=<0.0001, NT vs B6_BP_dslf 185.2 ug/kg P value=0.0024 , BLM vs B6_BP_dslf 185.2 ug/kg P value=0.0344, NT n=11, BLM n=17, B6_BP_dslf 46.3 ug/kg n=17, B6_BP_dslf 185.2 ug/kg n=19). b) Histograms of the frequency of intensities from HR-uCT scans on Day 21 (data represented as mean intensity values NT n=9, BLM n=10, B6_BP_dslf 46.3 ug/kg n=6, B6_BP_dslf 185.2 ug/kg n=9). c) Static Compliance as measured by the SCIREQ flexiVent FX at Day 21 (data represented as mean ± SEM, Tukey's t-test, NT vs BLM P value=<0.0001, NT vs B6_BP_dslf 46.3 ug/kg P value=<0.0001, NT vs B6_BP_dslf 185.2 ug/kg P value=<0.0001,BLM vs B6_BP_dslf 185.2 ug/kg P value=0.0388, NT n=13, BLM n=13, B6_BP_dslf 46.3 ug/kg n=13, B6_BP_dslf 185.2 ug/kg n=14). d) Immunofluorescence histological staining of collagen type-I and α-SMA for NT, BLM and OA B6_BP_dslf 185.2 ug/kg (scale bar=50 um). e) Representative images from western blot analysis for pro-fibrotic markers: Fibronectin, Collagen 1, p-SMAD2 with B-actin loading control. Quantification of Collagen 1 and p-SMAD are represented in Fig 4e, f. f) Quantification of Fibronectin western blot (data represented as mean ± SD, Tukey's t-test, NT vs BLM P value=0.0105, BLMvB6_BP_dslf 185.2 ug/kg P value=0.013, NT n=3, BLM n=3, B6_BP_dslf 46.3 ug/kg n=3, B6_BP_dslf 185.2 ug/kg n=3). g) Representative Sirius Red images and quantification of percentage red area (Tukey's t-test, NT vs BLM P value=0.0099, BLM vs B6_BP_dslf 185.2 ug/kg P value=0.0272, NT n=3, BLM n=4, B6_BP_dslf 46.3 ug/kg n=3, B6_BP_dslf 185.2 ug/kg n=4). h) Total BALF Cell Counts from recovered BALF fluid on Day 21 (mean ± SD, Tukey's t-test, NT vs BLM P value=0.0102, NT vs B6_BP_dslf 46.3 ug/kg P value=0.0469, NT n=8, BLM n=11, B6_BP_dslf 46.3 ug/kg n=3, B6_BP_dslf 185.2 ug/kg n=7). Differential cell counts i) Macrophage (mean ± SD, Tukey's t-test, NT vs BLM P value=<0.0001, NT vs B6_BP_dslf 46.3 ug/kg P value=<0.0001, T vs B6_BP_dslf 185.2 ug/kg P value=<0.0001, , NT n=6, BLM n=9, B6_BP_dslf 46.3 ug/kg n=3, B6_BP_dslf 185.2 ug/kg n=6). j) Lymphocyte (mean ± SD, Tukey's t-test, NT vs BLM P value=<0.0001, NT vs B6_BP_dslf 46.3 ug/kg P value=<0.0001, NT vs B6_BP_dslf 185.2 ug/kg P value=<0.0001, NT n=6, BLM n=9, B6_BP_dslf 46.3 ug/kg n=3, B6_BP_dslf 185.2 ug/kg n=7), and k) PMNs (mean ± SD,Tukey's t-test, NT vs BLM P value=0.0369, NT n=6, BLM n=7, B6_BP_dslf 46.3 ug/kg n=3, B6_BP_dslf 185.2 ug/kg n=6). All data were analyzed using a two-tailed Tukey's t-test following ANOVA: * p-value< 0.05, ** p-value<0.01, *** p-value<0.001, **** p-value<0.0001, and were adjusted for multiple comparisons.

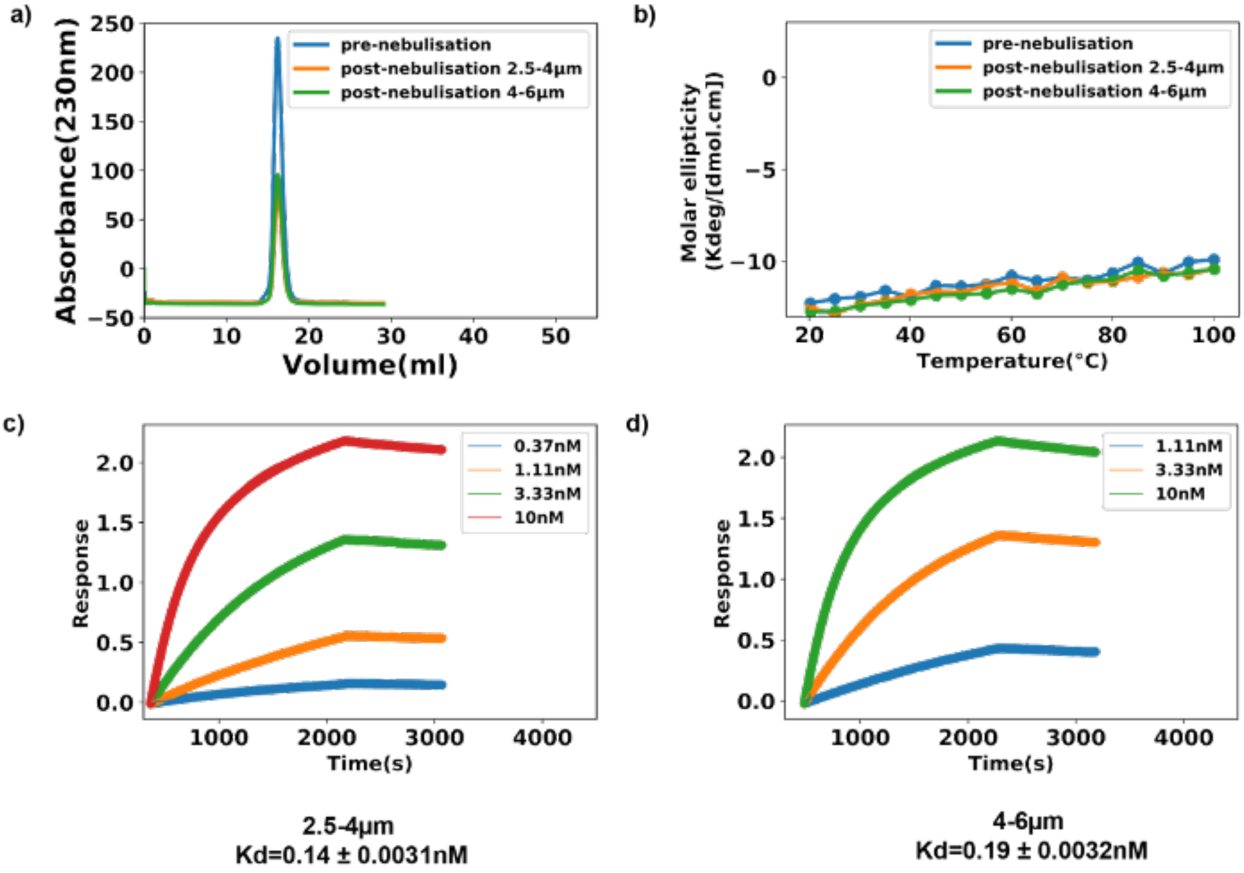

Supplementary Fig. 17: Stability of B6_BP_dslf following nebulization. a) Size-exclusion chromatography pre- and post-nebulization. b) Thermal melt of B6_BP_dslf pre- and post-nebulization using circular dichroism spectroscopy. The protein remains highly thermostable following nebulization. c,d) BLI binding experiment using aerosolized B6_BP_dslf and human αvβ6 integrin. B6_BP_dslf binds to αvβ6 with similar affinity post-nebulization for both particle sizes.

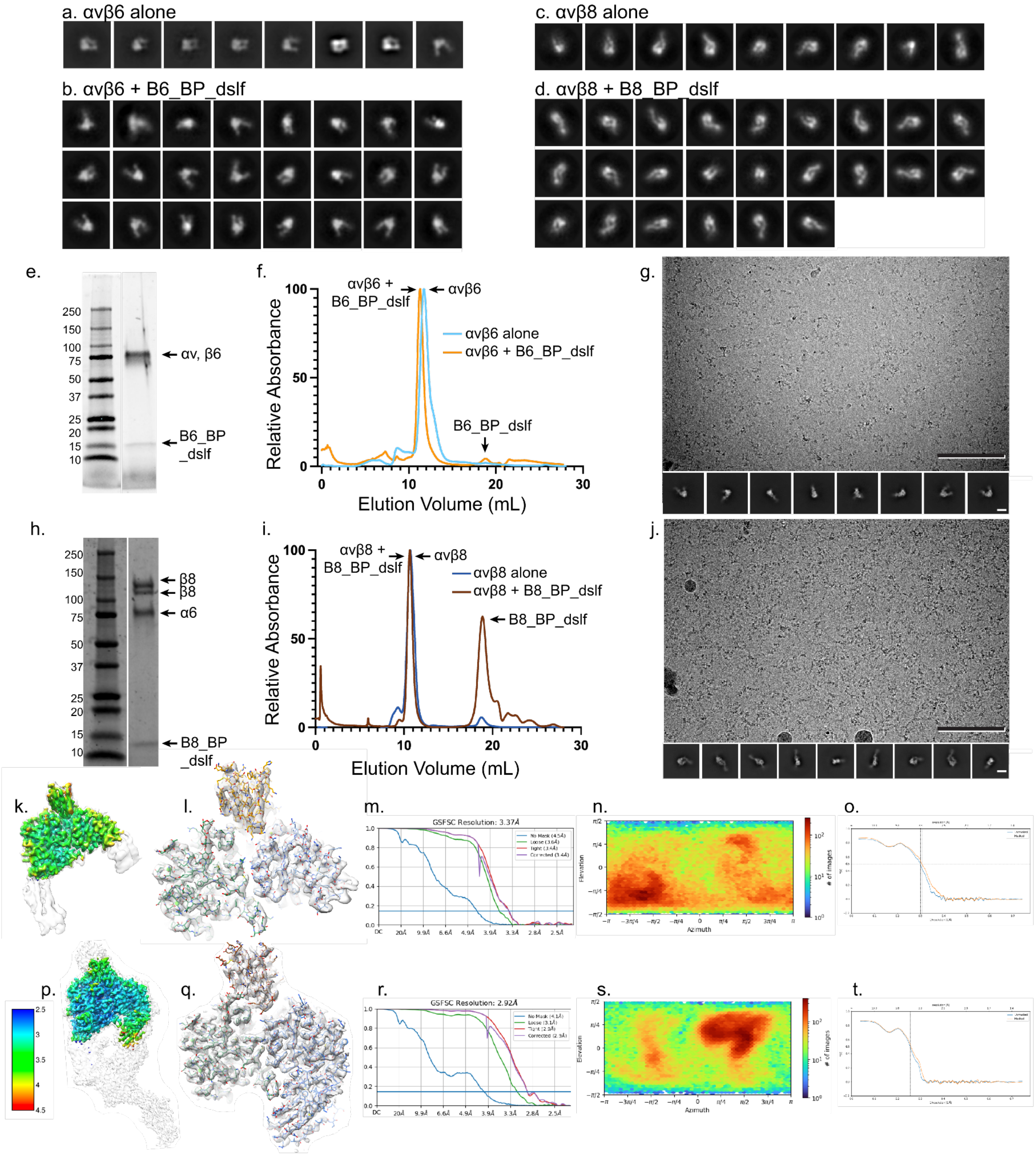

Supplementary Fig. 18: Negative stain 2D EM class averages, SDS-PAGE, SEC, cryoEM micrographs, cryoEM 2D class averages, and 3D reconstruction statistics of αvβ6 and αvβ8 integrin alone and in complex with respective selective minibinders B6_BP_dslf and B8_BP_dslf. a-d) Negative stain EM 2D class averages of (a) αvβ6 and (c) αvβ8 alone, and complexes of (b) αvβ6 + B6_BP_dslf and (d) αvβ8 + B8_BP_dslf. **e, h)** SDS-PAGE of complexes of (e) αvβ6 + B6_BP_dslf and (i) αvβ8 + B8_BP_dslf. Each complex formation and subsequent SDS-PAGE gel was carried one time and

was not repeated. **f, i)** Size-exclusion chromatography of (f) αvβ6 alone and in complex with B6_BP_dslf and (j) αvβ8 alone and in complex with B8_BP_dslf using a Superdex 200 Increase 10/300 SEC column (Cytiva). **g, j)** Representative motion corrected micrographs and 2D cryoEM class averages of (g) αvβ6 + B6_BP_dslf and (**j**) αvβ8 + B8_BP_dslf particles suspended in vitreous ice. Micrograph scale bars is 100 nm, 2D class averages scale bars are XXXX nm. For αvβ6 + B6_BP_dslf, two independent datasets were collected; for αvβ8 + B8_BP_dslf three independent datasets were collected, with thousands of similar micrographs (see Supplementary Fig.19 for exact numbers). **k-r)** 3D reconstruction statistics including maps colored by local resolution (k,p), close up of map and model (l,q), FSC (m,r), angular distribution heat map (n,s), and map to model GSC (o,t) for αvβ6 + B6_BP_dslf (k-o), and αvβ8 + B8_BP_dslf (p-t).

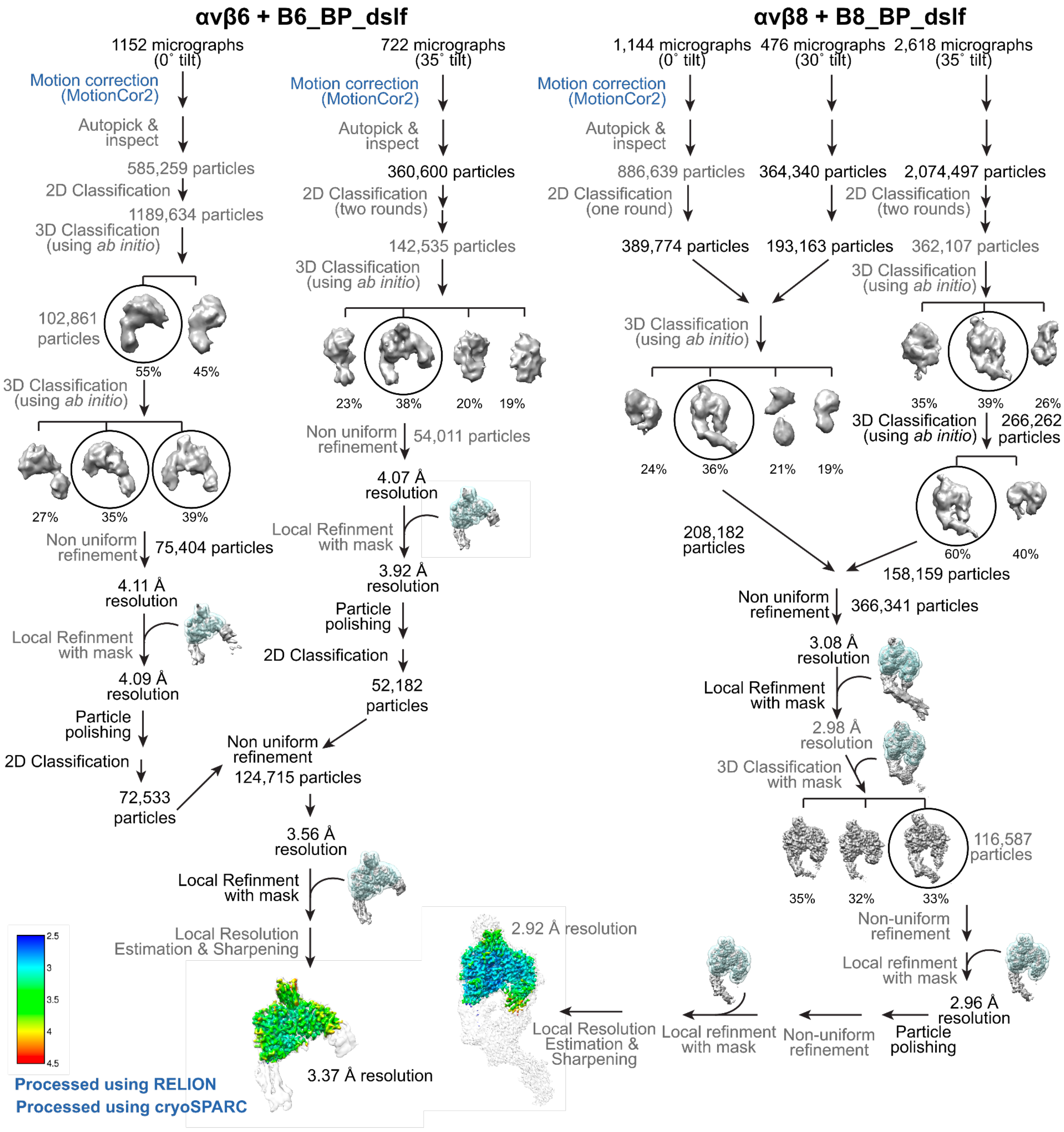

Supplementary Fig. 19: Processing schematic for cryoEM of αvβ6 + B6_BP_dslf and αvβ8 + B8_BP_dslf complexes. A schematic showing the processing, classification, and refinement workflow of the αvβ6 + B6_BP_dslf and αvβ8 + B8_BP_dslf complexes. Particle numbers at key steps and percentages for each class are indicated. Final maps are colored on the same scale, as indicated, based on local resolution estimates.

Supplementary Tables

Supplementary Table 1: Kinetic analysis of BLI binding of purified mutants against titrations of biotinylated human αvβ6 or αvβ8.

Mutant	Receptor	K_{on} ($M^{-1}s^{-1}$)	K_{dis} (s^{-1})	K_D (nM)
**E13T (B6B8_BP)**	h-αvβ6	$1.68x10^5 \pm 1.60x10^2$	$5.60x10^{-5} \pm 2.26x10^{-7}$	0.33 ± 0.0014
**M15R**	h-αvβ6	$1.75x10^5 \pm 1.26x10^2$	$6.65x10^{-5} \pm 1.74x10^{-7}$	0.38 ± 0.0010
**A39K**	h-αvβ6	$1.27x10^5 \pm 2.91x10^1$	$8.16x10^{-5} \pm 1.23x10^{-7}$	0.64 ± 0.0010
**G64R**	h-αvβ6	$1.41x10^5 \pm 1.89x10^2$	$1.34x10^{-4} \pm 3.08x10^{-7}$	0.95 ± 0.0025
**E13TG64R**	h-αvβ6	$1.12x10^5 \pm 8.77x10^1$	$8.87x10^{-5} \pm 1.60x10^{-7}$	0.79 ± 0.0016
**M15RG64R**	h-αvβ6	$1.04x10^5 \pm 1.72x10^2$	$7.71x10^{-5} \pm 3.19x10^{-7}$	0.74 ± 0.0033
**A39KG64R (B6_BP)**	h-αvβ6	$9.92x10^4 \pm 9.83x10^1$	$8.82x10^{-5} \pm 1.85x10^{-7}$	0.89 ± 0.0021
**E13TM15RA39K**	h-αvβ6	$8.43x10^4 \pm 1.32x10^2$	$9.10x10^{-5} \pm 2.62x10^{-7}$	1.08 ± 0.0035
**E13TM15RA39KG64R**	h-αvβ6	$1.04x10^5 \pm 8.88x10^1$	$8.31x10^{-5} \pm 1.63x10^{-7}$	0.79 ± 0.0017
**av6_3**	h-αvβ6	$8.16x10^4 \pm 1.37x10^2$	$9.67x10^{-5} \pm 4.43x10^{-7}$	1.19 ± 0.0058
**B6_BP_dslf**	h-αvβ6	$7.44x10^4 \pm 5.5x10^1$	$3.08x10^{-5} \pm 1.00x10^{-7}$	0.50 ± 0.0010
**B8_BP_dslf**	h-αvβ8	$8.03x10^4 \pm 1.70x10^2$	$2.39x10^{-4} \pm 7.83x10^{-7}$	1.97 ± 0.0012
**avb8_12**	h-αvβ8	$1.19x10^5 \pm 1.22x10^3$	$3.97x10^{-5} \pm 1.00x10^{-7}$	0.33 ± 0.0010
**avb8_12**	h-αvβ6	$3.47x10^5 \pm 1.07x10^3$	$3.92x10^{-4} \pm 5.33x10^{-7}$	1.13 ± 0.0038

Supplementary Table 2: Affinity and selectivity comparison of B6_BP against other leading reported αvβ6 inhibitors.

Integrin	B6_BP Affinity (nM)[1]	Fold Selectivity	GSK3008348 (IC50 nM)[2]	Fold Selectivity	A20FMDV2 (nM)[3]	Fold selectivity	Knottin R01 (nM)[4,*]	Fold selectivity	264-RAD (nM)	Fold selectivity
αvβ6	0.11		0.012		3		3.6		.01	
αvβ1	>200	>1818	2.6	190	Not reported	N/A	Not reported	Not reported	Not reported	Not reported
αvβ3	>200	>1818	37.4	3375	>10000	>3333	>10	>2.77	Does not bind	N/A
αvβ5	>200	>1818	11.7	1035	>100000	>33330	>10	>2.77	Does not bind	N/A
αvβ8	580	>5000	2.1	182	Not reported	Not reported	Not reported	Not reported	Binds (Kd not reported)	
α5β1	>200	>1818	97.2	6911	>10000	>33330	>10	>2.77	Does not bind	N/A
α8β1	>200	>1818	0.3	26	Not reported	Not reported	Not reported	Not reported	Not reported	Not reported
αiibβ3	>200	>1818	10000	917814	Not reported	Not reported	Not reported	Not reported	Not reported	Not reported

1: Measured using stable K562 cell lines overexpressing corresponding integrins.

2: Measured by competition assay using radio-labelled ligands.

3: Measured by competitive ELISA

4: Measured by integrin protein capture on immobilized knottins

*: This variant has been subjected to directed evolution in subsequent publications.

Supplementary Table 3. Predicted and observed interactions between integrins αvβ6/αvβ8 and designed minibinders.

αvβ6 - B6_BP_dslf *predicted* interactions by design (confirmed interactions shown in **bold**)				αvβ6 - B6_BP_dslf *observed* interactions in cryoEM (predicted interactions shown in **bold**)			
**αv**	**β6**	**minibinder**	**interaction type**	**αv**	**β8**	**minibinder**	**interaction type**
150		10	hydrogen	213		10	hydrogen
177		10	hydrogen	**218**		**10**	**hydrogen**
178		10	hydrogen	**218**		**10**	**salt**
**218**		**10**	**hydrogen**		**126**	**12**	**hydrogen**
150		10	salt		127	12	hydrogen
**218**		**10**	**salt**		**218**	**12**	**hydrogen**
	**126**	**12**	**hydrogen**		**219**	**12**	**hydrogen**

αv	β6	minibinder	interaction type	αv	β8	minibinder	interaction type
	**219**	**12**	**hydrogen**		**129**	**19**	**hydrogen**
	**218**	**12**	**hydrogen**		**316**	**41**	**hydrogen**
	**129**	**19**	**hydrogen**		**182**	**63**	**hydrogen**
	185	19	hydrogen		**183**	**63**	**hydrogen**
	254	39	hydrogen		**129**	**19**	**salt**
	**316**	**41**	**hydrogen**		**316**	**41**	**salt**
	**182**	**63**	**hydrogen**				
	**183**	**63**	**hydrogen**				
	**129**	**19**	**salt**				
	**316**	**41**	**salt**				

**αvβ8 - B8_BP_dslf *predicted* interactions by design** (confirmed interactions shown in **bold**)				**αvβ8 - B8_BP_dslf *observed* interactions in** (predicted interactions shown in **bold**)			
**αv**	**β6**	**minibinder**	**interaction type**	**αv**	**β8**	**minibinder**	**interaction type**
150		10	hydrogen	**218**		**10**	**hydrogen**
**218**		**10**	**hydrogen**	**215**		**41**	**hydrogen**
**215**		**41**	**hydrogen**	119		66	hydrogen
119		63	hydrogen	178		67	hydrogen
150		10	salt	**218**		**10**	**salt**
**218**		**10**	**salt**		114	12	hydrogen
	98	12	hydrogen		115	12	hydrogen
	99	12	hydrogen		116	12	hydrogen
	189	12	hydrogen		207	12	hydrogen
	285	40	hydrogen		208	12	hydrogen
	285	40	salt		115	16	hydrogen
					304	40	hydrogen
					304	40	salt

Supplementary Table 4. Statistics of X-ray diffraction and structure refinement. [1]

Data collection and refinement statistics (molecular replacement)

	BP (PDB: 7LMV)	B6B8_BP_dslf (PDB: 7LMX)
**Data collection**		
Space group	$P3_1$	$P2_12_12_1$
Cell dimensions		
a, *b*, *c* (Å)	92.27, 92.27, 82.42	40.69, 64.1, 80.99
α, β, γ (°)	90, 90, 120	90, 90, 90
Resolution (Å)	40.26 - 1.90 (1.96 -1.90)*	50.00 - 1.80 (1.86 - 1.80)*
R_{merge}	0.093 (0.613)	0.087 (1.283)
$I / \sigma I$	10.4 (2.4)	13.5 (2.5)
Completeness (%)	99.9 (99.8)	98.9 (100.0)
Redundancy	5.6 (4.5)	6.0 (6.3)
**Refinement**		
Resolution (Å)	40.26 - 1.90 (1.96 - 1.90)	26.20 - 1.80 (1.86 - 1.80)
No. reflections	61653 (6206)	20060 (1978)
R_{work} / R_{free}		
No. atoms		

Protein	6444	1777
Ligand/ion	0	0
Water	334	91
B-factors		
Protein	36.56	42.99
Ligand/ion	0	0
Water	34.80	44.23
R.m.s. deviations		
Bond lengths (Å)	0.006	0.004
Bond angles (°)	0.560	0.606

*Single Crystal used for each data collection. *Values in parentheses are for highest-resolution shell.

Data collection, phasing and refinement statistics (MIR)

	Crystal 1 name	Crystal 2 name
**Data collection**		
Space group		
Cell dimensions		
a, *b*, *c* (Å)		
α, β, γ (°)		
Resolution (Å)	##(high res shell) *	
R_{sym} or R_{merge}	##(high res shell)	
$I / \sigma I$	##(high res shell)	
Completeness (%)	##(high res shell)	
Redundancy	##(high res shell)	
**Refinement**		
Resolution (Å)		
No. reflections		
R_{work} / R_{free}		
No. atoms		
Protein		
Ligand/ion		
Water		
B-factors		
Protein		
Ligand/ion		
Water		
R.m.s deviations		
Bond lengths (Å)		
Bond angles (°)		

*Number of xtals for each structure should be noted in footnote. *Values in parentheses are for highest-resolution shell.

[AU: Equations defining various *R*-values are standard and hence are no longer defined in the footnotes.] [AU: Phasing data should be reported in Methods section.]
[AU: Ramachandran statistics should be in Methods section at end of Refinement subsection.]
[AU: Wavelength of data collection, temperature and beamline should all be in Methods section.]

Data collection, phasing and refinement statistics for MAD (SeMet) structures

	Native		Crystal 1 name			Crystal 2 name	
**Data collection**							
Space group			common #			common #	
Cell dimensions							
a, *b*, *c* (Å)			common #			common #	
α, β, γ (°)			common #			common #	
		Peak	*Inflection*	*Remote*	*Peak*	*Inflection*	*Remote*
Wavelength		#	#	#	#	#	#
Resolution (Å)		#	#	#	#	#	#
R_{sym} or R_{merge}		#	#	#	#	#	#
$I / \sigma I$		#	#	#	#	#	#
Completeness (%)		#	#	#	#	#	#
Redundancy		#	#	#	#	#	#
**Refinement**							
Resolution (Å)			common #			common #	
No. reflections							
R_{work} / R_{free}							
No. atoms							
Protein							
Ligand/ion							
Water							
B-factors							
Protein							
Ligand/ion							
Water							
R.m.s deviations							
Bond lengths (Å)							
Bond angles (°)							

*Number of xtals for each structure should be noted in footnote. *Values in parentheses are for highest-resolution shell.

[AU: Equations defining various *R*-values are standard and hence are no longer defined in the footnotes.]
[AU: Phasing data should be reported in Methods section.]
[AU: Ramachandran statistics should be in Methods section at end of Refinement subsection.]
[AU: Wavelength of data collection, temperature and beamline should all be in Methods section.]

Supplementary Table 5. Exclusion criteria for bleomycin-induced pulmonary fibrosis.

**Treatment Group** (Supplementary Figs. 13, 14)	**Exclusion Criteria A**[1]	**Exclusion Criteria B**	**Exclusion Criteria C**	**Included in study**
NT	0	0	0	29
BLM	20	0	0	14
BLM + IP	18	0	0	6
**Treatment Group** (Figure 4, Supplementary Figs. 15, 16)				
BLM	1	5	8	10
BLM + IP	0	5	10	9
Inhalations[2]	0	16	12	9

1. These criteria are as follows: Exclusion Criteria A: no development of fibrosis, Exclusion Criteria B: culled because of morbid weight loss, and Exclusion Criteria C: unexpected deaths from experimental procedures
2. n=4 43.6 µg/kg, n=5 185.2 µg/kg

Supplementary References

1. John, A. E. *et al.* Translational pharmacology of an inhaled small molecule αvβ6 integrin inhibitor for idiopathic pulmonary fibrosis. *Nat. Commun.* **11**, 4659 (2020).
2. Strauch, E.-M., Fleishman, S. J. & Baker, D. Computational design of a pH-sensitive IgG binding protein. *Proc. Natl. Acad. Sci. U. S. A.* **111**, 675–680 (2014).
3. Strauch, E.-M. *et al.* Computational design of trimeric influenza-neutralizing proteins targeting the hemagglutinin receptor binding site. *Nat. Biotechnol.* **35**, 667–671 (2017).
4. Rocklin, G. J. *et al.* Global analysis of protein folding using massively parallel design, synthesis, and testing. *Science* **357**, 168–175 (2017).

5. Dong, X., Hudson, N. E., Lu, C. & Springer, T. A. Structural determinants of integrin β-subunit specificity for latent TGF-β. *Nat. Struct. Mol. Biol.* **21**, 1091–1096 (2014).

6. Nishimura, S. L., Sheppard, D. & Pytela, R. Integrin alpha v beta 8. Interaction with vitronectin and functional divergence of the beta 8 cytoplasmic domain. *J. Biol. Chem.* **269**, 28708–28715 (1994).

7. Weinreb, P. H. *et al.* Function-blocking integrin alphavbeta6 monoclonal antibodies: distinct ligand-mimetic and nonligand-mimetic classes. *J. Biol. Chem.* **279**, 17875–17887 (2004).

8. Wang, J. *et al.* Atypical interactions of integrin αVβ8 with pro-TGF-β1. *Proc. Natl. Acad. Sci. U. S. A.* **114**, E4168–E4174 (2017).

9. Jacob, C. H. A. *et al.* Amino acid compounds and methods of use. *Patent* (2021).

10. Emsley, P. & Cowtan, K. Coot: model-building tools for molecular graphics. *Acta Crystallogr. D Biol. Crystallogr.* **60**, 2126–2132 (2004).

11. Davis, I. W. *et al.* MolProbity: all-atom contacts and structure validation for proteins and nucleic acids. *Nucleic Acids Res.* **35**, W375–83 (2007).

12. Potter, C. S. *et al.* Leginon: a system for fully automated acquisition of 1000 electron micrographs a day. *Ultramicroscopy* **77**, 153–161 (1999).

13. Punjani, A., Rubinstein, J. L., Fleet, D. J. & Brubaker, M. A. cryoSPARC: algorithms for rapid unsupervised cryo-EM structure determination. *Nat. Methods* **14**, 290–296 (2017).

14. Rohou, A. & Grigorieff, N. CTFFIND4: Fast and accurate defocus estimation from electron micrographs. *J. Struct. Biol.* **192**, 216–221 (2015).

15. Abe, M. *et al.* An Assay for Transforming Growth Factor-β Using Cells Transfected with a Plasminogen Activator Inhibitor-1 Promoter-Luciferase Construct. *Analytical Biochemistry* vol. 216 276–284 (1994).

16. Eberlein, C. *et al.* A human monoclonal antibody 264RAD targeting αvβ6 integrin reduces tumour growth and metastasis, and modulates key biomarkers in vivo. *Oncogene* **32**, 4406–4416 (2013).

17. Cash, J. N. *et al.* Development of a small-molecule screening method for inhibitors of cellular response to myostatin and activin A. *J. Biomol. Screen.* **18**, 837–844 (2013).

18. Stockis, J. *et al.* Blocking immunosuppression by human Tregs in vivo with antibodies targeting integrin αVβ8. *Proc. Natl. Acad. Sci. U. S. A.* **114**, E10161–E10168 (2017).

19. Devos, F. C. *et al.* Forced expiration measurements in mouse models of obstructive and restrictive lung diseases.

Respir. Res. **18**, 123 (2017).

20. Gilhodes, J.-C. *et al.* Quantification of Pulmonary Fibrosis in a Bleomycin Mouse Model Using Automated Histological Image Analysis. *PLoS One* **12**, e0170561 (2017).

21. Hübner, R.-H. *et al.* Standardized quantification of pulmonary fibrosis in histological samples. *Biotechniques* **44**, 507–11, 514–7 (2008).

22. Appendix A: Stains and Solutions Used in Hematology and Cytology. in *Exotic Animal Hematology and Cytology* (ed. Campbell, T. W.) 377–381 (John Wiley & Sons, Inc., 2015).

23. Lewis, J. B. *et al.* Transgenic up-regulation of Claudin-6 decreases fine diesel particulate matter (DPM)-induced pulmonary inflammation. *Environ. Sci. Pollut. Res. Int.* **25**, 18179–18188 (2018).

24. Schindelin, J. *et al.* Fiji: an open-source platform for biological-image analysis. *Nat. Methods* **9**, 676–682 (2012).

25. Namimatsu, S., Ghazizadeh, M. & Sugisaki, Y. Reversing the effects of formalin fixation with citraconic anhydride and heat: a universal antigen retrieval method. *J. Histochem. Cytochem.* **53**, 3–11 (2005).

26. Valdoz, J. C. *et al.* Soluble ECM promotes organotypic formation in lung alveolar model. *Biomaterials* **283**, 121464 (2022).
