# Supplementary material for: De novo design of highly selective miniprotein inhibitors of integrins αvβ6 and αvβ8
Source: Nat Commun. 2023 Sep 13;14:5660. doi: 10.1038/s41467-023-41272-z (PMC10500007; doi:10.1038/s41467-023-41272-z)
Supplement: Supplementary file 3 — Description of Additional Supplementary Files [file 41467_2023_41272_MOESM3_ESM.pdf]

Description of Additional Supplementary Files

Supplementary Data 1: Sequences of all designs and evolved variants reported in this paper.

Supplementary Data 2: Forward mutagenic primer sequences used for directed evolution.

Supplementary Data 3: Reverse mutagenic primer sequences used for directed evolution.

Supplementary Data 4: Integrin αv136 and αv138 headpiece and ectodomain amino acid sequences.

Supplementary Data 5: Synthetic Scheme and QC of PLN-74809

Supplementary Data 6: CryoEM data collection, refinement, and validation statistics.
