# Supplementary material for: De novo design of highly selective miniprotein inhibitors of integrins αvβ6 and αvβ8
Source: Nat Commun. 2023 Sep 13;14:5660. doi: 10.1038/s41467-023-41272-z (PMC10500007; doi:10.1038/s41467-023-41272-z)
Supplement: Supplementary file 7 — Supplementary Data 4 [file 41467_2023_41272_MOESM7_ESM.pdf]

>Human αv headpiece

MAFPPRRRLRLGPRGLPLLLSGLLLPLCRAFNLDVDSPAEYSGPEGSYFGFAVDFFVPSASSRMFLLVGAPKANTTQPGIVEGGQVL
KCDWSSTRRCQPIEFDATGNRDYAKDDPLEFKSHQWFGASVRSKQDKILACAPLYHWRTEMKQEREPVGTCFLQDGTKTVEYAPC
RSQDIDADGQGFCQGGFSIDFTKADRVLLGGPGSFYWQGQLISDQVAEIVSKYDPNVYSIKYNNQLATRTAQAIFDDSYLGYSVAVG
DFNGDGIDDFVSGVPRAARTLGMVYIYDGKNMSSLYNFTGEQMAAYFGFSVAATDINGDDYADVFIGAPLFMDRGSDGKLQEVGQV
SVSLQRASGDFQTTKLNGFEVFARFGSAIAPLGDLDQDGFNDIAIAAPYGGEDKKGIVYIFNGRSTGLNAVPSQILEGQWAARSGCPP
SFGYSMKGATDIDKNGYPDLIVGAFGVDRAILYRARPVITVNAGLEVYPSILNQDNKTCSLPGTALKVSCFNVRFCLKADGKGVLPRKL
NFQVELLLDKLKQKGAIRRALFLYSRSPSHSKNMTISRGGLMQCEELIAYLRDESEFRDKLTPITIFMEYRLDYRTAADTTGLQPILNQF
TPANISRQAHILLTGGLEVLFQGPGENAQLEKELQALEKENAQLEWELQALEKELAQGGSHHHHHH

>Human β6 headpiece

MAFPPRRRLRLGPRGLPLLLSGLLLPLCRAHVQGGCALGGAETCEDCLLIGPQCAWCAQENFTHPSGVGERCDTPANLLAKGCQL
NFIENPVSQVEILKNKPLSVGRQKNSSDIVQIAPQSLILKLRPGGAQTLQVHVRQTEDYPVDLYYLMDLSASMDDDLNTIKELGSRLSK
EMSKLTSNFRLGFGSFVEKPVSPFVKTTPEEIANPCSSIPYFCLPTFGFKHILPLTNDAERFNEIVKNQKISANIDTPEGGFDAIMQAAV
CKEKIGWRNDSLHLLVFVSDADSHFGMDSKLAGIVCPNDGLCHLDSKNEYSMSTVLEYPTIGQLIDKLVQNNVLLIFAVTQEQVHLYE
NYAKLIPGATVGLLQKDSGNILQLIISAYEELRSEVELEVLGDTEGLNLSFTAICNNGTLFQHQKKCSHMKVGDTASFSVTVNIPHCERR
SRHIIIKPVGLGDALELLVSPECNCDCQKEVEVNSSKCHHGNGSFQCGVCACHPGHMGPRCESGGLEVLFQGPSGGAQLKKKLQA
LKKKNAQLKWKLQALKKKLAQGGSHHHHHH

>Human β8 headpiece

MAFPPRRRLRLGPRGLPLLLSGLLLPLCRAEDNRCASSNAASCARCLALGPECGWCVQEDFISGGSRSERCDIVSNLISKGCSVDSI
EYPSVHVIIPTENEINTQVTPGEVSIQLRPGAEANFMLKVHPLKKYPVDLYYLVDVSASMHNNIEKLNSVGNDLSRKMAFFSRDFRLGF
GSYVDKTVSPYISIHPERIHNQCSDYNLDCMPPHGYIHVLSLTENITEFEKAVHRQKISGNIDTPEGGFDAMLQAAVCESHIGWRKEAK
RLLLVMTDQTSHLALDSKLAGIVCPNDGNCHLKNNVYVKSTTMEHPSLGQLSEKLIDNNINVIFAVQGKQFHWYKDLLPLLPGTIAGEI
ESKAANLNNLVVEAYQKLISEVKVQVENQVQGIYFNITAICPDGSRKPGMEGCRNVTSNDEVLFNVTVTMKKCDVTGGKNYAIIKPIGF
NETAKIHIHRNCSCQCEDNRGPKGKCVDETFLDSKCFQCDENKSGGLEVLFQGPSGGAQLKKKLQALKKKNAQLKWKLQALKKKLA
QGGSHHHHHH

>Human αv ectodomain

MAFPPRRRLRLGPRGLPLLLSGLLLPLCRAFNLDVDSPAEYSGPEGSYFGFAVDFFVPSASSRMFLLVGAPKANTTQPGIVEGGQVL
KCDWSSTRRCQPIEFDATGNRDYAKDDPLEFKSHQWFGASVRSKQDKILACAPLYHWRTEMKQEREPVGTCFLQDGTKTVEYAPC
RSQDIDADGQGFCQGGFSIDFTKADRVLLGGPGSFYWQGQLISDQVAEIVSKYDPNVYSIKYNNQLATRTAQAIFDDSYLGYSVAVG
DFNGDGIDDFVSGVPRAARTLGMVYIYDGKNMSSLYNFTGEQMAAYFGFSVAATDINGDDYADVFIGAPLFMDRGSDGKLQEVGQV
SVSLQRASGDFQTTKLNGFEVFARFGSAIAPLGDLDQDGFNDIAIAAPYGGEDKKGIVYIFNGRSTGLNAVPSQILEGQWAARSGCPP
SFGYSMKGATDIDKNGYPDLIVGAFGVDRAILYRARPVITVNAGLEVYPSILNQDNKTCSLPGTALKVSCFNVRFCLKADGKGVLPRKL
NFQVELLLDKLKQKGAIRRALFLYSRSPSHSKNMTISRGGLMQCEELIAYLRDESEFRDKLTPITIFMEYRLDYRTAADTTGLQPILNQF
TPANISRQAHILLDCGEDNVCKPKLEVSVDSDQKKIYIGDDNPLTLIVKAQNQGEGAYEAELIVSIPLQADFIGVVRNNEALARLSCAFK
TENQTRQVVCDLGNPMKAGTQLLAGLRFSVHQQSEMDTSVKFDLQIQSSNLFDKVSPVVSHKVDLAVLAAVEIRGVSSPDHVFLPIP
NWEHKENPETEEDVGPVVQHIYELRNNGPSSFSKAMLHLQWPYKYNNNTLLYILHYDIDGPMNCTSDMEINPLRIKISSLQTTEKNDT
VAGQGERDHLITKRDLALSEGDIHTLGCGVAQCLKIVCQVGRLDRGKSAILYVKSLLWTETFMNKENQNHSYSLKSSASFNVIEFPYK
NLPIEDITNSTLVTTNVTWGIQPAPMTGGLEVLFQGPGENAQLEKELQALEKENAQLEWELQALEKELAQGGSHHHHHH

>Human β6 ectodomain

MAFPPRRRLRLGPRGLPLLLSGLLLPLCRAHVQGGCALGGAETCEDCLLIGPQCAWCAQENFTHPSGVGERCDTPANLLAKGCQL
NFIENPVSQVEILKNKPLSVGRQKNSSDIVQIAPQSLILKLRPGGAQTLQVHVRQTEDYPVDLYYLMDLSASMDDDLNTIKELGSRLSK
EMSKLTSNFRLGFGSFVEKPVSPFVKTTPEEIANPCSSIPYFCLPTFGFKHILPLTNDAERFNEIVKNQKISANIDTPEGGFDAIMQAAV
CKEKIGWRNDSLHLLVFVSDADSHFGMDSKLAGIVCPNDGLCHLDSKNEYSMSTVLEYPTIGQLIDKLVQNNVLLIFAVTQEQVHLYE
NYAKLIPGATVGLLQKDSGNILQLIISAYEELRSEVELEVLGDTEGLNLSFTAICNNGTLFQHQKKCSHMKVGDTASFSVTVNIPHCERR
SRHIIIKPVGLGDALELLVSPECNCDCQKEVEVNSSKCHHGNGSFQCGVCACHPGHMGPRCECGEDMLSTDSCKEAPDHPSCSGR
GDCYCGQCICHLSPYGNIYGPYCQCDNFSCVRHKGLLCGGNGDCDCGECVCRSGWTGEYCNCTTSTDSCVSEDGVLCSGRGDC
VCGKCVCTNPGASGPTCERCPTCGDPCNSKRSCIECHLSAAGQAREECVDKCKLAGATISEEEDFSKDGSVSCSLQGENECLITFLI
TTDNEGKTIIHSINEKDCPKPPNSGGLEVLFQGPSGGAQLKKKLQALKKKNAQLKWKLQALKKKLAQGGSHHHHHH

>Human β8 ectodomain

MAFPPRRRLRLGPRGLPLLLSGLLLPLCRAEDNRCASSNAASCARCLALGPECGWCVQEDFISGGSRSERCDIVSNLISKGCSVDSI
EYPSVHVIIPTENEINTQVTPGEVSIQLRPGAEANFMLKVHPLKKYPVDLYYLVDVSASMHNNIEKLNSVGNDLSRKMAFFSRDFRLGF
GSYVDKTVSPYISIHPERIHNQCSDYNLDCMPPHGYIHVLSLTENITEFEKAVHRQKISGNIDTPEGGFDAMLQAAVCESHIGWRKEAK
RLLLVMTDQTSHLALDSKLAGIVCPNDGNCHLKNNVYVKSTTMEHPSLGQLSEKLIDNNINVIFAVQGKQFHWYKDLLPLLPGTIAGEI
ESKAANLNNLVVEAYQKLISEVKVQVENQVQGIYFNITAICPDGSRKPGMEGCRNVTSNDEVLFNVTVTMKKCDVTGGKNYAIIKPIGF
NETAKIHIHRNCSCQCEDNRGPKGKCVDETFLDSKCFQCDENKCHFDEDQFSSESCKSHKDQPVCSGRGVCVCGKCSCHKIKLGK
VYGKYCEKDDFSCPYHHGNLCAGHGECEAGRCQCFSGWEGDRCQCPSAAAQHCVNSKGQVCSGRGTCVCGRCECTDPRSIGR
FCEHCPTCYTACKENWNCMQCLHPHNLSQAILDQCKTSCALMEQQHYVDQTSECFSSPSSGGLEVLFQGPSGGAQLKKKLQALKK
KNAQLKWKLQALKKKLAQGGSHHHHHH
