# Supplementary material for: De novo design of highly selective miniprotein inhibitors of integrins αvβ6 and αvβ8
Source: Nat Commun. 2023 Sep 13;14:5660. doi: 10.1038/s41467-023-41272-z (PMC10500007; doi:10.1038/s41467-023-41272-z)
Supplement: Supplementary file 8 — Supplementary Data 5 [file 41467_2023_41272_MOESM8_ESM.pdf]

Research Summary:

No.	Compound ID	Structure	Quantity	Timeline	Status
1	Compound 6	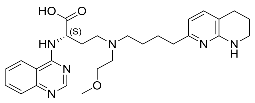	10.0 g	05/09/2022	In progress

Project ID: UW-20220210

(05/25/2022-05/31/2022)

Structure	Compound ID	Compound 6	Compound ID (WX)	N/A
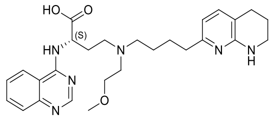	Quantity	10.0 g	Purity	95%
	Starting date	02/21/2022	Timeline	05/09/2022
	Status	In progress	Chemist	Xiaoyan Li

Synthetic Scheme:

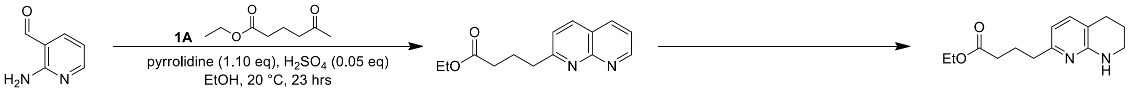

1	2	2 (used)	Conditions	3	3 (used)
5.00 g	4.50 g	1.50 g	Pd/C, H_2 (15 Psi), MeOH, 20 °C	1.40 g	1.40 g
45.0 g	62.0 g	1.50 g	PtO_2, H_2 (15 Psi), MeOH, 20 °C	1.30 g	1.30 g
50.0 g	60.0 g	1.50 g	Pd/C, H_2 (50 Psi), MeOH, 20 °C	1.30 g	9.80 g
		10.0 g	Pd/C, H_2 (15 Psi), EtOH, 20 °C	9.80 g	51.0 g
		52.0 g	Pd/C, H_2 (15 Psi), EtOH, 20 °C	51.0 g	62.6 g
		60.0 g	Pd/C, H_2 (15 Psi), EtOH, 20 °C	62.6 g	

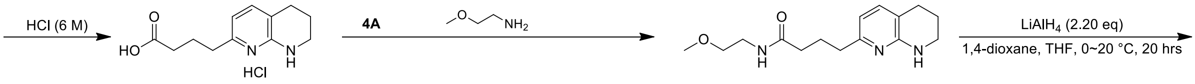

4	4 (used)	Conditions	5	5 (used)
2.34 g (crude)	2.34 g	Cpd_4A (1.50 eq), HATU (1.45 eq), DIEA (2.00 eq), DCM, 20 °C, 2 hrs	1.93 g (purity: 40.5%, purified by pre-HPLC) → 500 mg	210 mg
2.00 g (crude)	1.00 g	Cpd_4A (1.50 eq), HOBt (1.50 eq), EDCI(2.00 eq), DIEA (6.00 eq), DMF, 20 °C, 4 hrs	210 mg (purity: 90%)	1.80 g
11.8 g (crude)				10.0 g
53.0 g (pure)	1.00 g	Cpd_4A (5.00 eq), HOBt (1.10 eq), DIEA (6.00 eq), EDCI(1.10 eq), DMF, 20-60 °C, 20 hrs	~22% of Cpd_4 was remained, 400 mg obtained	11.0 g
60.0 g	100 mg	Cpd_4A (5.00 eq), HATU (1.10 eq), DIEA (2.00 eq), DCM, 25 °C, 2 hrs	76 mg (purity: 93%)	
	2.00 g	Cpd_4A (5.00 eq), HATU (1.10 eq), DIEA (2.00 eq), DCM, 25 °C, 4 hrs	644 mg (purity: 92%) after reversed phase	
	5.00 g	Cpd_4A (5.00 eq), HATU (1.10 eq), DIEA (3.00 eq), DCM, 25 °C, 2 hrs	550 mg (86.6% purity) after reversed phase, then C.C purification	
	34.0 g	Cpd_4A (5.00 eq), HATU (1.10 eq), DIEA (3.00 eq), DCM, 25 °C, 2 hrs	21.0 g (98.2% purity) after reversed-phase HPLC (netural condition) purification	

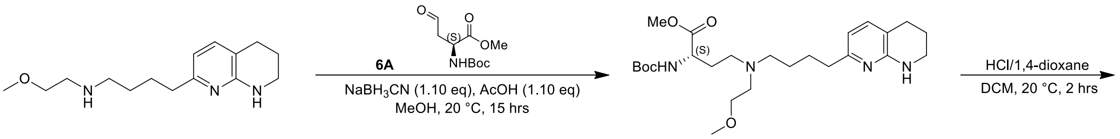

6	6 (used)	7
171 mg		53 mg after Pre-TLC purification
1.02 g		821 mg after prep-HPLC purification
9.50 g	6.60 g	9.80 g
10.3 g	13.2 g	19.4 g

PRIVATE & TOP CONFIDENTIAL

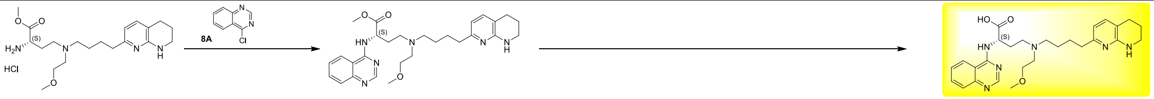

Cpd 8		Conditions	Cpd 11
50 mg (curde)		8A (1.30 eq), $NaHCO_3$ (5.40 eq), THF/H_2O, 70 °C, 2 hrs	16.0 mg (free base)
786 mg (crude)		8A (1.10 eq), $NaHCO_3$ (5.40 eq), THF/H_2O, 70 °C, 2 hrs	1.24 g (crude)
9.10 g (crude)	317 mg	8A (1.10 eq), $NaHCO_3$ (9.00 eq), THF/H_2O, 70 °C, 12 hrs	85.0 mg (3TFA salt)
18.4 g (crude)	220 mg	8A (1.10 eq), DIEA (5.90 eq), DMF, 60 °C, 12 hrs	105 mg (3TFA salt)
	200 mg	8A (1.10 eq), DIEA (5.90 eq), i-PrOH, 60 °C, 12 hrs	70.3% of Cpd_11 was formed
	2.06 g	8A (1.10 eq), DIEA (5.90 eq), i-PrOH, 60 °C, 5 hrs	2.10 g (3TFA salt, 93.7% purity) after pre-HPLC (TFA condition) purification
	6.55 g	8A (1.10 eq), DIEA (5.90 eq), i-PrOH, 60 °C, 5 hrs	2.40 g (free base, 96.3% purity, 74% e.e),after pre-HPLC purification
	18.4 g	8A (1.10 eq), DIEA (6.20 eq), i-PrOH, 60 °C, 5 hrs	9.80 g (free base, 95.0% purity, 74% e.e), after pre-HPLC purification

Cpd 11			Conditions	Compound 6	
16.0 mg			LiOH (2.00 eq), THF, MeOH, H_2O, 25 °C, 2 hrs	LCMS showed 33.8% compound 6 was formed	
427 mg (crude)			LiOH (2.00 eq), THF, MeOH, H_2O, 25 °C, 2 hrs	36.0 mg (purity: 99.2%) after twice purification	about 1 g, 78.8% e.e value
803mg (crude)			LiOH (5.00 eq), THF, MeOH, H_2O, 25 °C, 20 hrs	90.0 mg (purity: 89.2%), 81.0 mg (purity: 83.8%)	
2.10 g			LiOH (5.00 eq), THF, MeOH, H_2O, 25 °C, 3 hrs	446 mg (TFA salt, purity: 93.8%)	
514 mg			LiOH (3.00 eq), THF, H_2O, 25 °C, 15 hrs	190 mg (TFA salt, purity: 95.5%)	
88 mg			LiOH (4.00 eq), $CaCl_2$ (16.0), i-PrOH/THF/H_2O, 25 °C	91.0 mg (TFA salt, purity: 98.9%)	
330 mg			LiOH (4.00 eq), $CaCl_2$ (16.0), i-PrOH/THF/H_2O, 25 °C	298 mg (TFA salt, purity: 95.1%)	
1.10 g	SFC → 843 mg, >99% e.e	117 mg	LiOH (6.00 eq), $CaCl_2$ (16.0), i-PrOH/THF/H_2O, 25 °C	131 mg (TFA salt, purity: 100%, 96.5% e.e value)	
10.0 g	SFC → 8.00 g, >99% e.e	720 mg	LiOH (6.00 eq), $CaCl_2$ (16.0), i-PrOH/THF/H_2O, 25 °C	2.10 g (TFA salt, purity: 97.5%), under HNMR checking	
		3.80 g	LiOH (6.00 eq), $CaCl_2$ (16.0), i-PrOH/THF/H_2O, 25 °C	1.50 g (TFA salt, purity: 91.1%), under secondary purification<br>1.60 g (TFA salt, purity: 71%), under secondary purification	

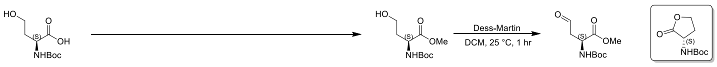

Cpd 9	Conditions	Cpd 10	Cpd 10 input	6A
1.00 g	MeI (1.00 eq), K_2CO_3 (1.00 eq), DMF, 20 °C, 15 hrs	500 mg of Cpd_10<br>373 mg of 10-BP	100 mg	crude confirmed by HNMR
1.00 g	MeI (1.20 eq), Cs_2CO_3 (0.50 eq), DMF, 20 °C, 15 hrs	866 mg	2.00 g	1.20 g yellow oil, pure
20.0 g	MeI (1.20 eq), Cs_2CO_3 (0.50 eq), DMF, 20 °C, 15 hrs	18.8 g	16.7 g	6.60 g
26.3 g	MeI (1.20 eq), Cs_2CO_3 (0.50 eq), DMF, 20 °C, 15 hrs	24.8 g	76.1 g	47.8 g
70.0 g	MeI (1.20 eq), Cs_2CO_3 (0.50 eq), DMF, 20 °C, 15 hrs	58.1 g		

Research progress:

By now, 2.10 g of Compound 6 is was obtained, with 97.5% purity under HNMR checking, 1.50 g of final Compound 6 is under secondary purification.

HPLC of Compound 6.pdf   LCMS of Compound 6.pdf   SFC of Compound 6.pdf

Confidential. For research only, Not for regulatory filing.

Compound ID: compound 6

EC7425-64-P1C DMSO Bruker_CD-G_400MHz

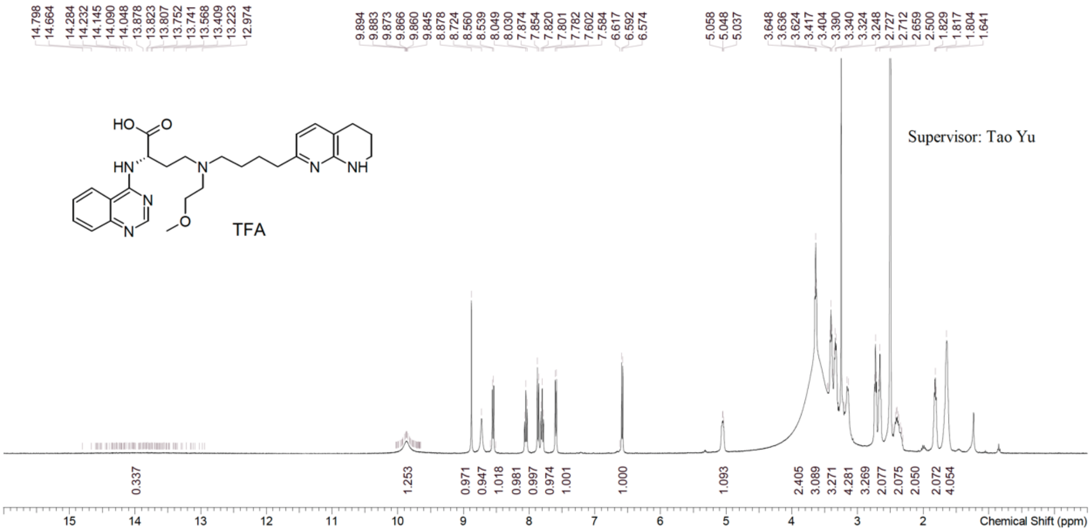

Supervisor: Tao Yu

Operator: Date:

If you have any question related to this spectrum, please contact yu_tao@wuxiapptec.com for support.
WuXi V-Lab Request compound synthesis with ease, speed, and flexibility Please click below link to download the APP: http://www.wuxiapptec.com/WXVLab
LabNetwork Millions of discovery products from hundreds of suppliers, including > 125,000 WuXi novel catalog products for medicinal chemistry & drug discovery. Online at www.LabNetwork.com

1H-NMR of PLN-74809 in DMSO

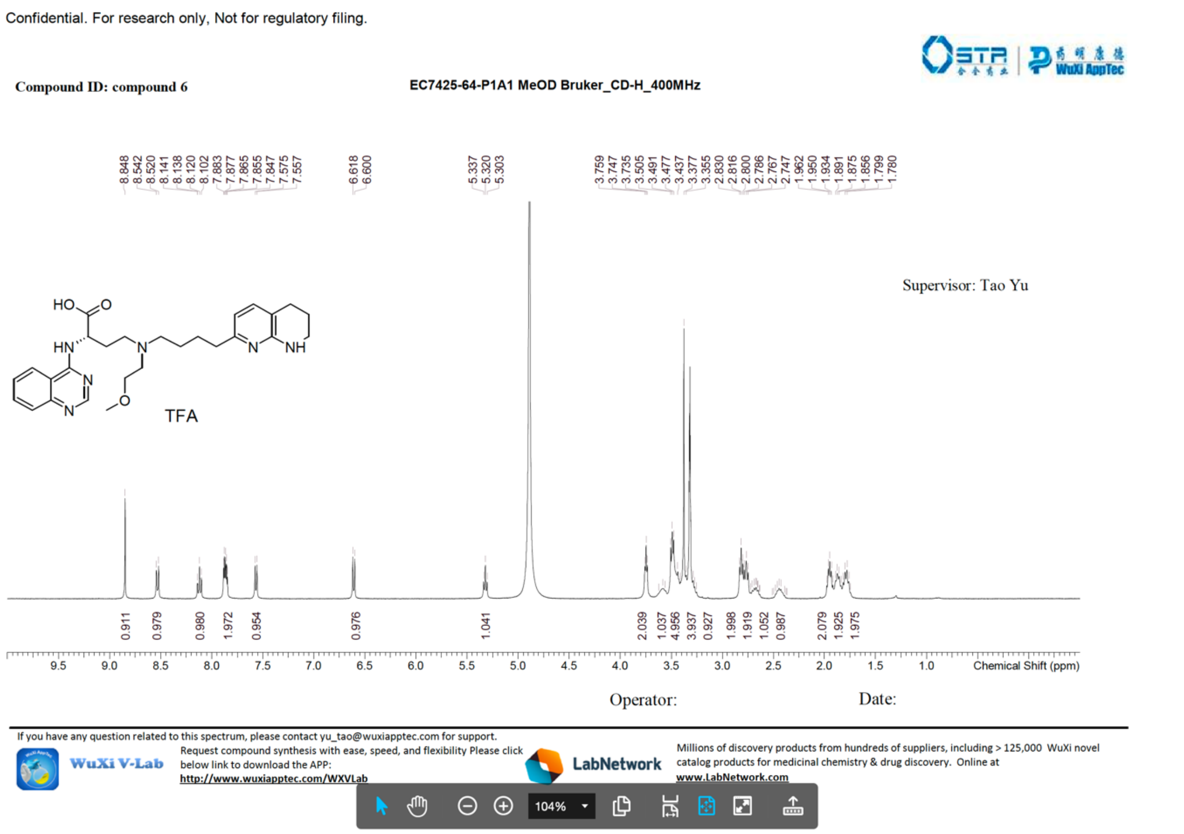

1H-NMR of PLN-74809 in MeOD

LCMS Report

==================================

Compound ID : compound6
Sample ID : EC7425-64-P1F1
Injection Vol : 1ul
Location : vial79
Acq Method : D:\method\0-60AB_1min.lcm
Org DataFile : D:\DATA\2022\2205\220524\EC7425-64-P1F1.lcd
Injection Date : 2022/5/24 10:57:13
Instrument : CAS-CD-LCMS-AE

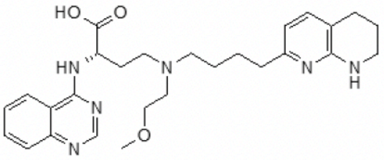

Exact Mass: 492.28

Chromatogram

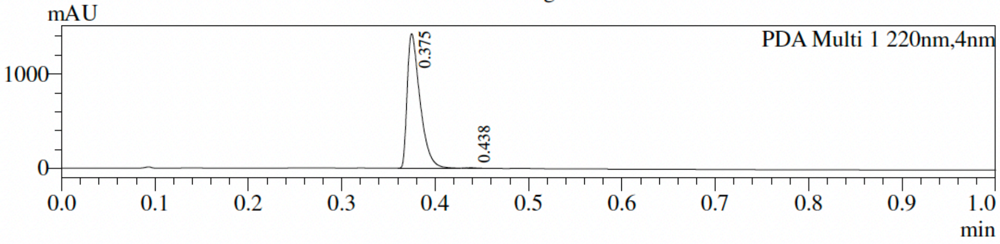

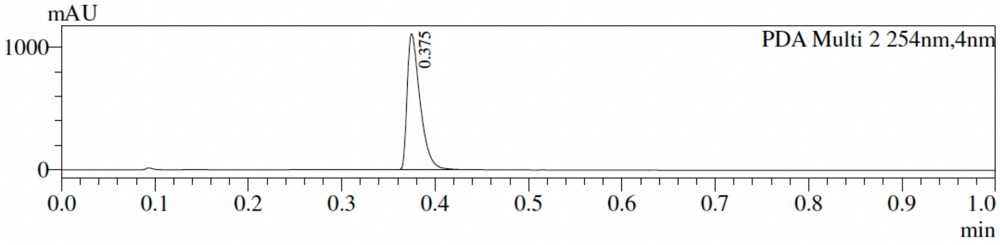

MS Chromatogram

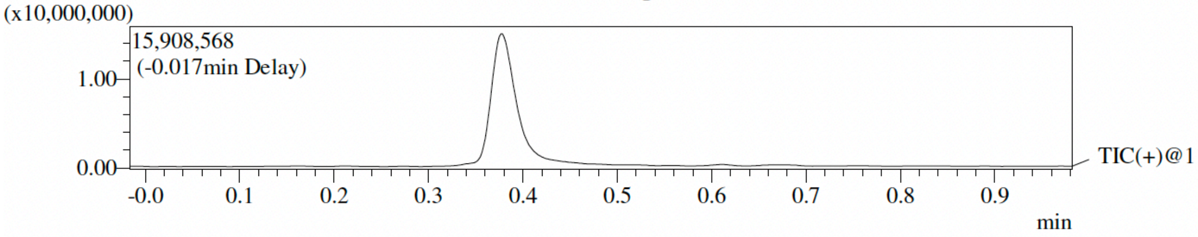

==================================

Integration Result

==================================

PDA Ch1 220nm

Peak#	Ret. Time	Height	Height%	USP Width	Area	Area%
1	0.375	1424835	99.610	0.025	1358521	99.724
2	0.438	5575	0.390	0.018	3760	0.276

PDA Ch2 254nm

Peak#	Ret. Time	Height	Height%	USP Width	Area	Area%
1	0.375	1108752	100.000	0.025	1060524	100.000

PRIVATE & TOP CONFIDENTIAL

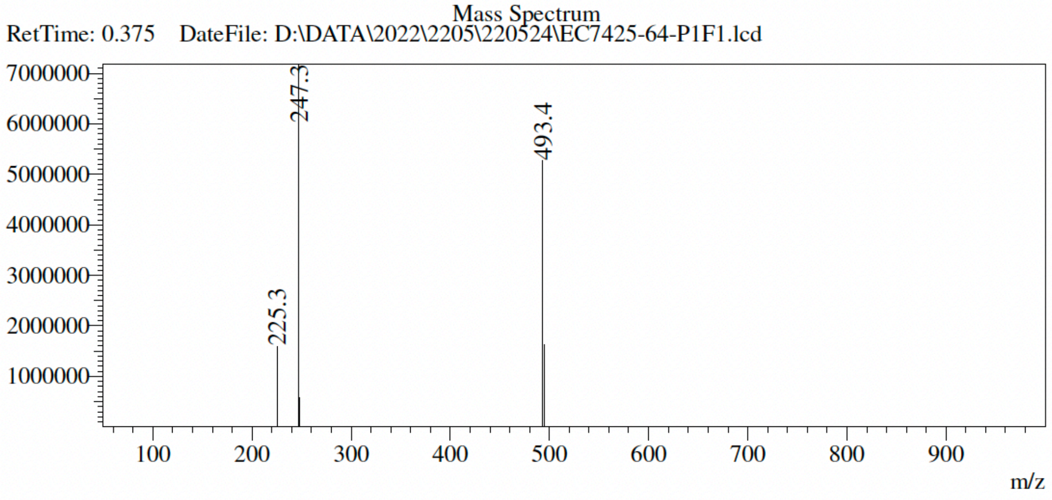

Mass Spec of PLN-74809

HPLC Report

==

Compound ID : compound 6
Sample ID : EC7425-64-P1F2
Injection Vol : 1ul
Location : vial40
Acq Method : D:\method\0-60AB_4min.lcm
Org DataFile : D:\DATA\2022\2205\220524\EC7425-64-P1F2.lcd
Injection Date : 5/24/2022 10:28:54 AM
Instrument : CAS-CD-HPLC-N

Chromatogram

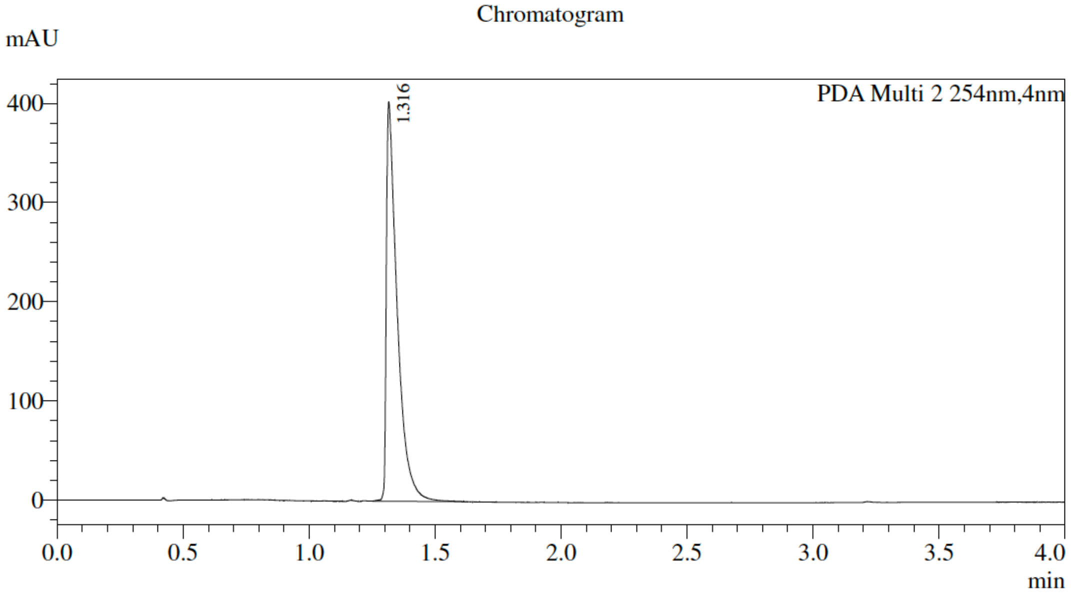

Integration Result

PDA Ch2 254nm

Peak#	Ret. Time	USP Width	Height	Height%	Area	Area%
1	1.316	0.078	402876	100.000	1206852	100.000
Total			402876	100.000	1206852	100.000

PRIVATE & TOP CONFIDENTIAL

HPLC Report

=====================================

Compound ID	: compound 6
Sample ID	: EC7425-64-P1F2
Injection Vol	: 1ul
Location	: vial40
Acq Method	: D:\method\0-60AB_4min.lcm
Org DataFile	: D:\DATA\2022\2205\220524\EC7425-64-P1F2.lcd
Injection Date	: 5/24/2022 10:28:54 AM
Instrument	: CAS-CD-HPLC-N

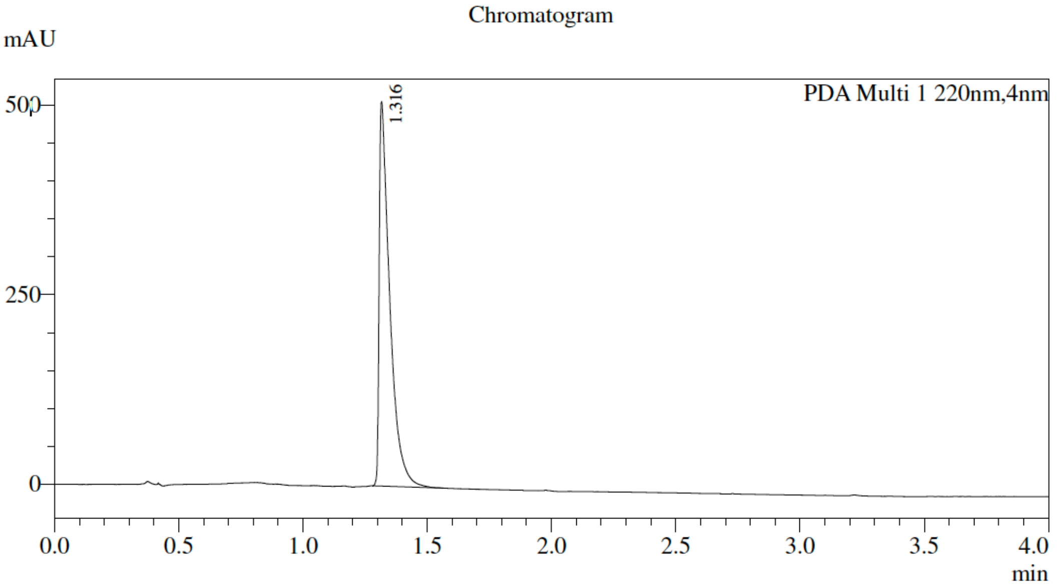

Integration Result

PDA Ch1 220nm

Peak#	Ret. Time	USP Width	Height	Height%	Area	Area%
1	1.316	0.078	506894	100.000	1498596	100.000
Total			506894	100.000	1498596	100.000

HPLC trace of PLN-74809

PRIVATE & TOP CONFIDENTIAL

Chiral SFC Report

Compound ID : compound 6
Sample ID : EC7425-64-P1A2_A1
Injection Vol : 5ul
Location : vial56
Acq Method : D:\method\AD-3-EtOH(DEA)-40-3mL-35T.lcm
Org DateFile : D:\DATA\2022\202205\20220524\EC7425-64-P1A2_A1.lcd
Injection Date : 5/24/2022 1:47:05 PM
Instrument : CAS-CD-ANA-SFC-A(SHIMADZU LC-30ADsf)

Method details: "Column:Chiralpak AD-3 50x4.6mm I.D., 3um
Mobile phase: Phase A for CO2, and Phase B for EtOH(0.05%DEA);
Isocratic elution: EtOH (0.05% DEA) in CO2 for 40%
Flow rate: 3mL/min;Detector: PDA;
Column Temp: 35C;Back Pressure: 100Bar"

Chromatogram

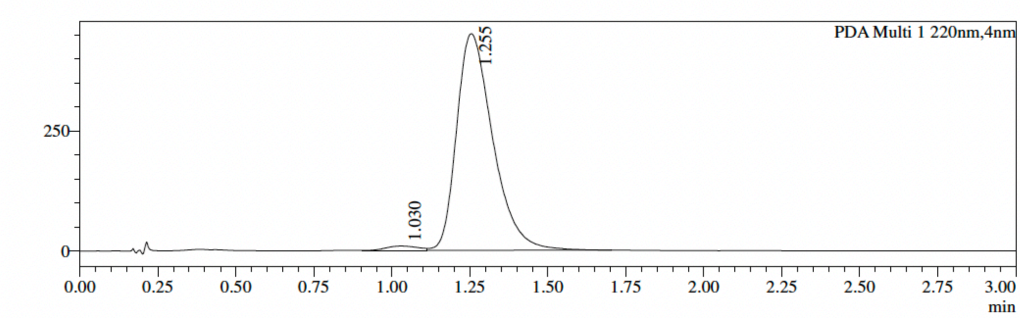

1 PDA Multi 1 / 220nm,4nm

MS Chromatogram

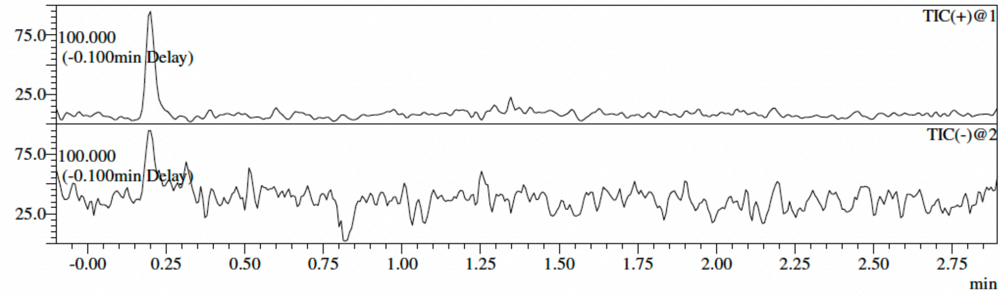

Integration Result

Peak Table

PDA Ch1 220nm

Peak#	Ret. Time	Height	Height%	USP Width	Area	Area%	Resolution(USP)
1	1.030	9249	2.013	0.209	64365	1.735	--
2	1.255	450095	97.987	0.209	3646163	98.265	1.075

Operator:______________

Date:_________________

PRIVATE & TOP CONFIDENTIAL

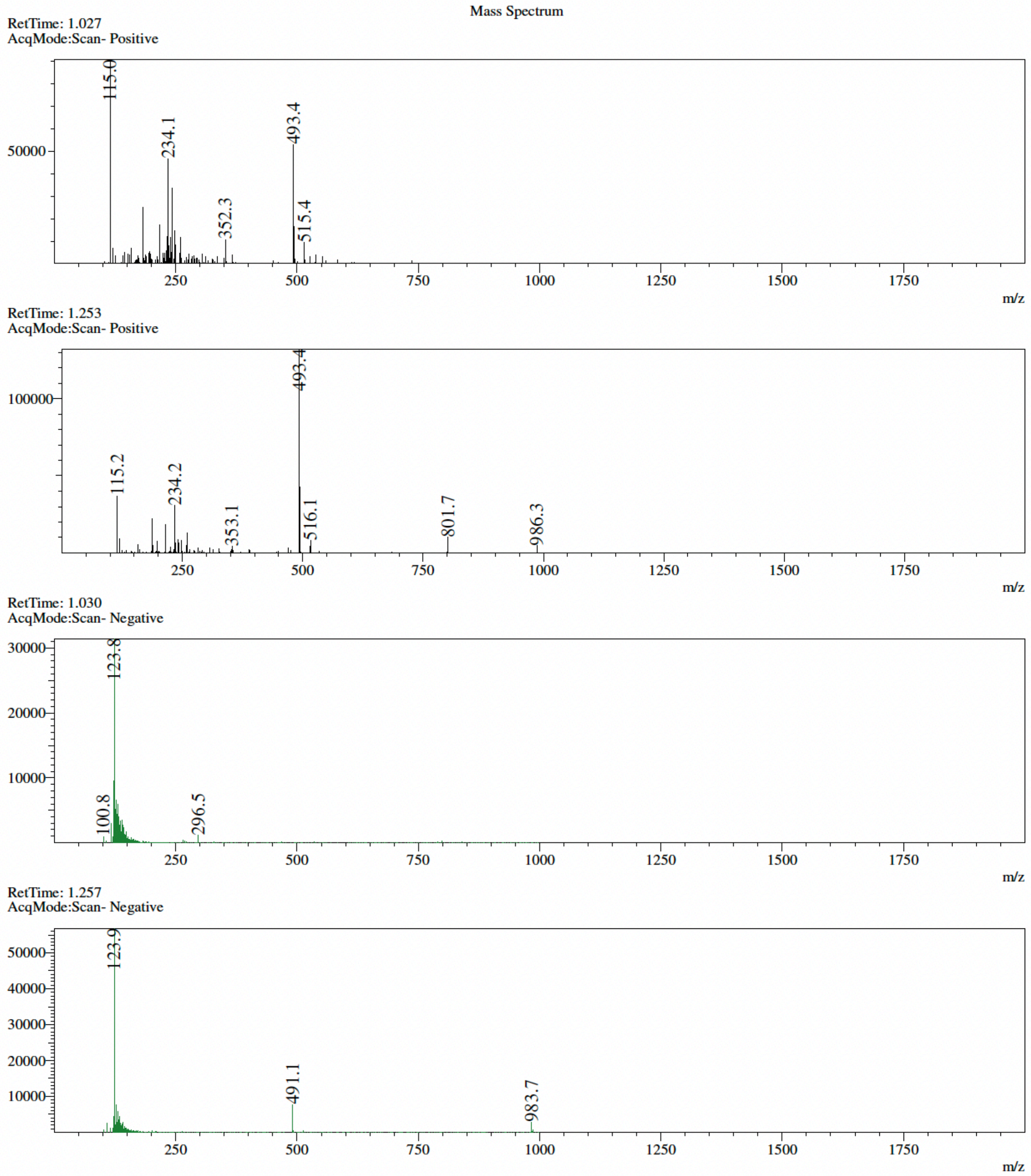

Chiral HPLC and Mass spec of PLN-74809
